# NorA Efflux Pump Inhibitors: Expanding SAR Knowledge of Pyrazolo[4,3‐*c*][1,2]benzothiazine 5,5‐Dioxide Derivatives

**DOI:** 10.1002/ardp.70000

**Published:** 2025-05-19

**Authors:** Giada Cernicchi, Alessandra Di Gregorio, Tommaso Felicetti, Elisa Rampacci, Giulia Casari, Tatiana Armeni, Brenda Romaldi, Ermelinda Zefaj, Fabrizio Passamonti, Serena Massari, Giuseppe Manfroni, Maria Letizia Barreca, Oriana Tabarrini, Carla Vignaroli, Stefano Sabatini

**Affiliations:** ^1^ Department of Pharmaceutical Sciences Università degli Studi di Perugia Perugia Italy; ^2^ Department of Life and Environmental Science Università Politecnica delle Marche Ancona Italy; ^3^ Department of Veterinary Medicine Università degli Studi di Perugia Perugia Italy; ^4^ Department of Specialized Clinical Sciences and Odontostomatology Università Politecnica delle Marche Torrette di Ancona Italy

**Keywords:** antibiotic resistance breakers, ciprofloxacin, efflux pump inhibitor, NorA, pyrazolobenzothiazine

## Abstract

Antimicrobial resistance (AMR) represents a significant global concern, driven by the overuse of antibiotics. One of the principal mechanisms contributing to AMR is the activity of microbial efflux pumps (EPs), which expel diverse antibiotics out of bacterial cells, thereby rendering them ineffective. Our research aimed to expand the range of molecular classes that inhibit the *Staphylococcus aureus* EP NorA. In this study, starting from the hit compound pyrazolo[4,3‐*c*][1,2]benzothiazine 5,5‐dioxide **1**, previously reported as a NorA efflux pump inhibitor (EPI), we undertook medicinal chemistry efforts, which involved the iterative combination of the design and synthesis of new analogues with data obtained through ethidium bromide efflux inhibition assays. Subsequent synergistic assays with ciprofloxacin (CPX) against the resistant strain SA‐1199B led to the identification of three potent compounds (**3**, **10**, and **19**). The evaluation of these compounds in combination with CPX against NorA‐overexpressing and NorA‐knockout strains (SA‐K2378 and SA‐K1902, respectively) confirmed that the observed synergy with CPX is dependent on the presence of NorA. Additionally, the combination of NorA EPIs with CPX reduced biofilm production in NorA‐overexpressing strains. These findings enhance our understanding of the structure–activity relationship of pyrazolobenzothiazine derivatives and support the use of EtBr efflux assays for rapid NorA inhibitors' identification.

## Graphical abstract

The pyrazolo[4,3‐*c*][1,2]benzothiazine 5,5‐dioxide class was investigated with the aim of enlarging the array of *Staphylococcus aureus* NorA efflux pump inhibitors. The ethidium bromide efflux assay was used as a first indication of NorA inhibition; then, the ability of compounds to synergize with ciprofloxacin (CPX) against both wild‐type and resistant *S. aureus* strains was evaluated. The best derivatives (e.g., **10**) also showed the inhibition of biofilm formation when combined with CPX.

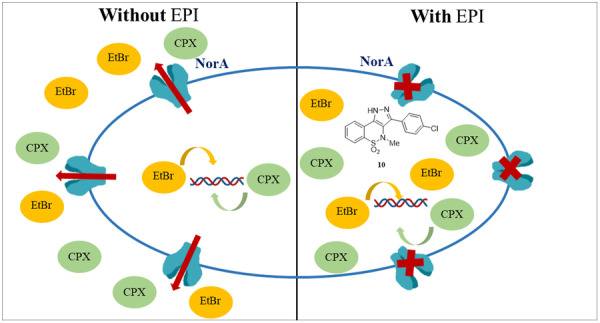

## 1 Introduction

Antimicrobial resistance (AMR) has now entered the public debate and is regarded as an urgent global public health concern to be addressed. The overuse and misuse of antibiotics have led to the development of survival mechanisms in microorganisms that enable them to resist antimicrobial agents [1]. A recent systematic analysis of AMR estimated that 7.7 million of deaths per year are associated with bacterial infections, with 4.95 million of these deaths related to AMR [2]. Accordingly, if this trend persists in the coming years, the forecast is for a worsening of the scenario, with 10 million deaths per year by 2050 [2, 3]. In light of this, AMR represents a significant and urgent global health concern, necessitating prompt and decisive action. To overcome resistance, two strategies can be used. The first is the discovery of new antimicrobial agents with innovative mechanisms of action [4]. The second is the development of antimicrobial resistance breakers (ARBs) [5]. These compounds are devoid of any antibacterial activity on their own and are used in combination with known antibiotics to which bacteria have become resistant. This allows the intracellular concentration of the antibiotics to be replenished, thereby restoring their efficacy [5]. Therefore, if pathogens develop resistance against novel antibacterial agents (first strategy), ARBs developed by the second approach are effectively designed to impede one or more resistance mechanisms exploited by microbes to evade antibiotic treatment.

Efflux pumps (EPs) are the initial mechanism by which bacteria prevent the intracellular accumulation of toxic compounds, including antimicrobial agents. Consequently, the development of EP inhibitors (EPIs) to be administered concurrently with a known antibiotic substrate represents an attractive strategy to overcome AMR [6]. EPs are transmembrane proteins classified based on both their structure and the energy utilized for the efflux, and can be grouped into different families [7, 8, 9]. The major facilitator superfamily (MFS) is the largest family of transporters present in all domains of life. It exploits a proton motive force as an energy source and comprises EPs that are capable of extruding a diverse range of structurally unrelated compounds, including various classes of antibacterials such as fluoroquinolones.

In this context, we have been engaged in the fight against AMR for years, developing EPIs against NorA, the most studied EP of *Staphylococcus aureus* [10, 11]. In recent times, significant advances have been made in the field of NorA EP research, particularly with regard to the three‐dimensional structure and mechanistic mode of action. Indeed, the two cryo‐EM structures of NorA in complex with antigen‐binding fragments that have been recently released represent a notable advancement in this regard [12, 13]. Conversely, the field of drug discovery has made minimal advancements, with no molecule having been advanced to preclinical studies and no crystals of NorA in complex with an inhibitor being documented in the literature. Moreover, the lack of biochemical and/or biophysical assays that can rapidly identify potential NorA inhibitors has significantly constrained research in this field. Therefore, investigation has primarily relied on phenotypic assays to determine the synergism of nonantibiotic molecules in combination with antibacterial NorA substrates (often fluoroquinolones) against *S. aureus* strains overexpressing *norA*. The subsequent validation of these potential NorA inhibitors identified through phenotypic screening represents a significant shortcoming in the search for NorA EPIs. Indeed, the lack of well‐defined assays to assess the selective inhibition of NorA represents a significant challenge in determining which of the reported inhibitors demonstrate a synergistic effect with antibacterial agents through the inhibition of NorA‐mediated efflux. In this regard, based on our experience in this field, we believe that the evaluation of NorA inhibition, indirectly assessed by monitoring the reduction of the efflux of the known NorA substrate, ethidium bromide (EtBr), represents a valid strategy for the rapid identification of preliminary NorA inhibitors and the validation of compounds that synergize with antibacterials as being real NorA EPIs. By leveraging data from the scientific literature on molecules identified as NorA EPIs through EtBr efflux inhibition tests on the *S. aureus* strain SA‐1199B overexpressing *norA*, we constructed and subsequently refined pharmacophore models based on a ligand‐based approach [14, 15]. This enabled the application of virtual screening and computer‐aided scaffold hopping strategies of the quinoline core, previously used by us to develop potent NorA EPIs [16, 17]. Nevertheless, the identification/design of new NorA EPIs based on the pharmacophore model is not without intrinsic limitations. Indeed, the identification of NorA inhibitors through the use of an existing pharmacophore model does not permit an update of the model itself. Consequently, the model is not subject to continuous optimization. Furthermore, although the model is technically viable, it is mainly based on data from a few molecular classes, primarily quinolines and indoles [10, 14]. It would therefore be optimal to identify novel NorA inhibitors with alternative scaffolds and characteristics using a conventional medicinal chemistry methodology. The results of these studies could then be used to extend the chemical space encompassed by the models, thereby enhancing their predictive capabilities.

**Figure 1:**
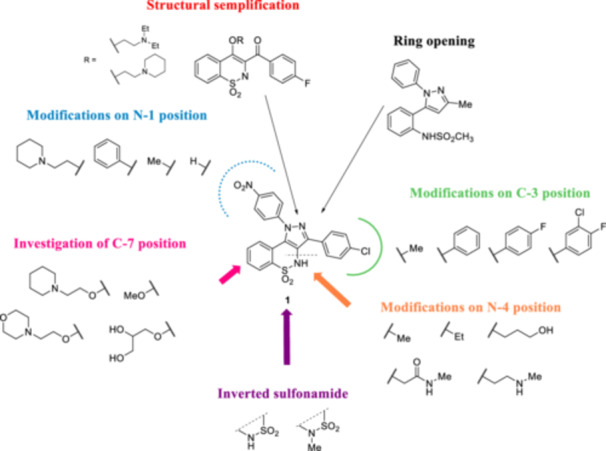
Modifications around the pyrazolobenzothiazine core designed starting from hit **1**.

In light of the aforementioned considerations, this study reports a novel series of analogues of compound **1**, previously identified by us as NorA EPI [18] and belonging to the less explored class of pyrazolo[4,3‐*c*] [1,2]benzothiazine‐5,5‐dioxide derivatives. It is noteworthy that the identification of compound **1** as NorA EPI was based on a computer‐aided approach that exploited the progenitor of the NorA pharmacophore models that have since been refined more recently. However, the recent implementations of the pharmacophore with literature data provided updated 3D pharmacophores that no longer considered compound **1** as a potential NorA EPI [14], likely due to the paucity of information surrounding the pyrazolo[4,3‐*c*]benzothiazine‐5,5‐dioxide class. Therefore, in this study, we report the design, synthesis, and NorA inhibition activity of 23 pyrazolobenzothiazine analogues (**2–24**—see Figure 1 and Table 1 for chemical structures) with the aim of exploring the structure–activity relationship (SAR) around this less explored class of derivatives. The EtBr efflux assay was initially utilized as the primary indicator of NorA inhibition, and subsequently, the ability of compounds to synergize with ciprofloxacin (CPX) against both wild‐type and resistant strains of *S. aureus* was evaluated.

## 2 Results and Discussion

### 2.1 Chemistry

**Scheme 1:**
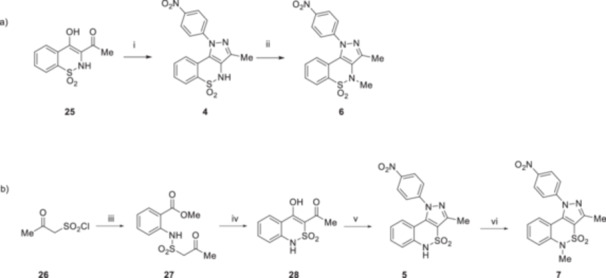
Synthesis of compounds **4–7**. Reagents and conditions: (a) (i) *p*‐nitro phenyl hydrazine, DMF, 100°C, 24 h, 50%; (ii) MeI, NaH, dry DMF, r.t, 4 h, 80%; (b) (iii) methyl anthranilate, Et_3_N, dry benzene, 0°C to 90°C, 90 min., 23% (iv) Na, EtOH, 70°C, 40 min, 68%; (v) *p*‐nitro phenyl hydrazine, EtOH, reflux, 24 h, 54%; and (vi) MeI, K_2_CO_3_, dry DMF, r.t, 4 h, 100%.

Derivatives **4** and **6** were synthesized as reported in Scheme 1a. Following a regioselective condensation previously reported by us [19, 20], compound **4** was re‐prepared by the reaction between compound **25** and the *p*‐nitro phenyl hydrazine using DMF as a solvent at 100°C. To further confirm the regioselectivity of this reaction, the molecular structure of **4** was also determined by performing a 2D NMR spectrum, which showed the diagnostic interaction observed between the H‐9 proton of the pyrazolobenzothiazine nucleus and the H‐2′ and H‐6′ protons of the N‐1 phenyl ring (Supporting Information S1: Figure S1). Compound **4** was then methylated using methyl iodide in the presence of sodium hydride as a base and dry DMF as the solvent to obtain compound **6** in good yield. Derivatives **5** and **7** were synthesized following the procedure reported in Scheme 1b. Chloro sulfonyl acetone **26**, prepared as reported in the literature [21], was reacted with methyl anthranilate in the presence of Et_3_N and using dry benzene as a solvent to obtain the intermediate **27**. Then, it was cyclized using sodium ethoxide, produced in situ, and EtOH as solvent, allowing for the synthesis of compound **28** in moderate yield. The subsequent formation of the pyrazolo benzothiazine core was performed by reacting intermediate **28** with *p*‐nitro phenyl hydrazine in EtOH to obtain the target compound **5**. To provide evidence of reaction regioselectivity, a 2D NMR spectrum was performed, which revealed the characteristic interaction between the H‐9 proton of the pyrazolobenzothiazine core of compound **5** and the H‐2′ and H‐6′ protons of the N‐1 phenyl ring (Supporting information S1: Figure S2). Compound **5** was subsequently *N*‐methylated using methyl iodide in the presence of K_2_CO_3_ and using dry DMF as a solvent, finally yielding the target compound **7**.

**Scheme 2:**
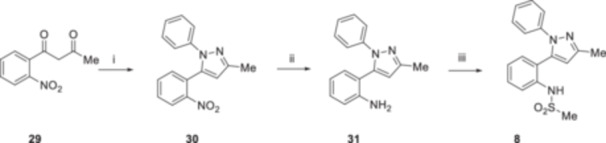
Synthesis of compound **8**. Reagents and Conditions: (i) phenyl hydrazine, EtOH, reflux, 12 h, 31%; (ii) Ni/Raney, EtOH, NH_2_NH_2_ x H_2_O, r.t., 90 min, 90%; and (iii) methane sulfonyl chloride, dry Pyr/dry DCM (1:1), r.t., 1 h, 17%.

Derivative **8** was synthesized according to the procedure reported in Scheme 2. Starting from compound **29**, the formation of the pyrazole core was performed using phenyl hydrazine in EtOH, leading to the intermediate **30**. Subsequently, the reduction of the nitro group was carried out using Raney/Ni as a catalyst in the presence of hydrazine monohydrate and using EtOH as a solvent to obtain the amino derivative **31** in good yield. At this point, we verified the regioselectivity of the preceding cyclization reaction by conducting a 2D NMR experiment, with the objective of exploiting the potential couplings in space of the two protons of the amino group. As expected, the characteristic interactions between the NH_2_ protons and the protons of the N‐1 phenyl ring were observed, thereby indirectly confirming the regioselectivity of the cyclization reaction that resulted in the formation of **30** (Supporting Information S1: Figure S3). Compound **31** was then reacted with methane sulfonyl chloride in a mixture of dry pyridine and dry DCM to obtain the target compound **8**.

**Scheme 3:**
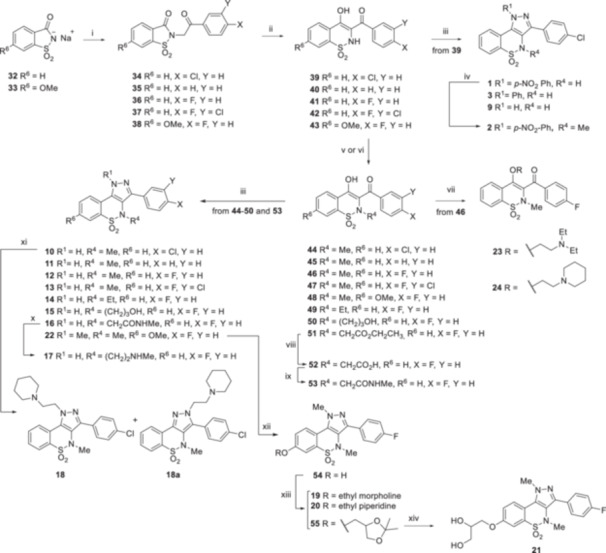
Synthesis of derivatives **2**, **3**, and **9**–**24**. Reagents and Conditions: (i) proper acetophenones, dry DMSO, 70°C, 40 min −3 h, 84%–100%; (ii) Na, EtOH, r.t. –60°C, 30 min −2 h, 64%−68%; (iii) methylhydrazine or hydrazine monohydrate or phenyl hydrazine or *p‐*nitro phenyl hydrazine, ultrasound or stirring at 65°C, 20 min – 3 h, 11%–80%; (iv) MeI, K_2_CO_3_, dry DMF, r.t., 2 h, 80%; (v) MeI or EtI or ethylbromo acetate, sol aqueous NaOH 1 N/EtOH (1:4), r.t., 1 h – overnight, 50%–82%; (vi) 3‐chloropropan‐1‐ol, 60% NaH, dry DMF, r.t., 24 h, 15%; (vii) proper chloroethylamino chains, K_2_CO_3_, dry DMF, 80°C, 2 h, 30%; (viii) LiOH 1 N, THF, r.t., 1 h, 74%; (ix) methylamine, Et_3_N, TBTU, dry THF, r.t., 2 h, 77%; (x) LiAlH_4_, dry THF, reflux, 48 h, 18%; (xi) chloroethylpiperidine, 60% NaH, dry THF, 0°C to r.t., 2 h, 21%; (xii) 2 M BBr_3_ in DCM, dry CH_2_Cl_2_, 0°C to reflux, 1 h, 89%; (xiii) Cl‐alkyl derivatives, Cs_2_CO_3_, dry DMF, 80°C, 90 min −5 h, 20%–60%; and (xiv) HCl 9%, MeOH, reflux, 1 h, 45%.

Derivatives **2**, **3**, and **9**–**24** were synthesized as depicted in Scheme 3, following the procedure previously reported by us [18], with some modifications. Starting from the commercially available saccharin sodium salt **32** and 6‐methoxysaccharin sodium salt **33**, prepared according to the method described in the literature [22, 23], the nucleophilic substitution was performed using properly functionalized 2‐bromo acetophenones in dry DMSO at 70°C, obtaining the intermediates **34–37** (from **32**) and **38** (from **33**). The subsequent ring expansion of **34–38** was performed using sodium ethoxide, produced in situ, in EtOH to yield intermediates **39**–**43** in moderate yields. Subsequently, through regioselective condensation [18], compound **39** was reacted with *p*‐nitro phenyl hydrazine to obtain compound **1,** which was methylated at the *N*‐4 position using methyl iodide in the presence of K_2_CO_3_ in EtOH to obtain the target compound **2**. In parallel, derivative **39** reacted with phenyl hydrazine or hydrazine monohydrate to yield compound **3** or **9**, respectively.

Intermediates **39–43** were also alkylated at the *N*‐4 position using methyl iodide or ethyl iodide or ethyl 2‐chloroacetate in a mixture of NaOH 1 M aqueous solution and EtOH (1:4), yielding intermediates **44**–**49** and **51**, while intermediate **50** was obtained from the reaction of **41** with 3‐chloropropan‐1‐ol in the presence of sodium hydride 60%, using dry DMF as a solvent at room temperature. Starting from derivative **46**, compounds **23** and **24** were obtained by performing *O*‐alkylation at the C‐3 position using 2‐chloro‐*N*,*N*‐diethyethy‐1‐amine and 2‐chloro‐ethylpiperidine, respectively, K_2_CO_3_ as a base, and dry DMF as the solvent. Under basic conditions, compound **51** was hydrolyzed to obtain the acid derivative **52,** which was reacted with methylamine in the presence of Et_3_N, TBTU, and dry THF to afford amide derivative **53** in good yield. Intermediates **44**–**47**, **49**, **50**, and **53** were then cyclized in the presence of hydrazine monohydrate to obtain the target compounds **10–16**. Subsequently, the alkylation of derivative **10** using chloro‐ethylpiperidine in the presence of 60% sodium hydride resulted in the formation of a complex mixture of both regioisomers **18** and **18a**, with a prevalence of the N‐1 alkylated analogue (compound **18**). Following purification by chromatography, the structure of the compound **18** was confirmed by a 2D NMR experiment, which revealed a NOE cross‐peak between the H‐9 proton of the pyrazolobenzothiazine nucleus and the NCH_2_CH_2_ protons of the N‐1 ethyl‐piperidine chain (Supporting information S1: Figure S4). Despite the unsuccessful isolation of the regioisomer **18a** with high purity following chromatography, the 2D NMR experiment unambiguously demonstrated the interactions between the N‐1 ethyl‐piperidine chain and the protons of the C‐3 phenyl ring (data not shown). Compound **17** was obtained by reducing derivative **16** in the presence of LiAlH_4_ and using dry THF as a solvent.

In the case of the synthesis of compound **22**, in which methylhydrazine was reacted with intermediate **48**, the regioselectivity of the reaction was demonstrated by a 2D NMR spectrum. Notably, a relevant NOE cross‐peak was observed between the H‐9 proton of the pyrazolobenzothiazine nucleus and the N‐1 methyl group (Supporting information S1: Figure S5). Subsequently, compound **22** was demethylated using BBr_3_ in dry DCM, affording the intermediate **54**, in good yield, which was *O*‐alkylated using properly chloro‐alkyl chains in the presence of Cs_2_CO_3_ to yield target compounds **19** and **20** and intermediate **55,** which was refluxed in a solution of 9% HCl in methanol to yield the target compound **21** in moderate yield.

### 2.2 Design and Synthesis of Pyrazolo[4,3‐*c*][1,2]benzothiazine 5,5‐Dioxide Derivatives 2–24 and Their Evaluation for *S. aureus* NorA Efflux Pump Inhibition

#### 2.2.1 EtBr efflux assays

**Figure 2:**
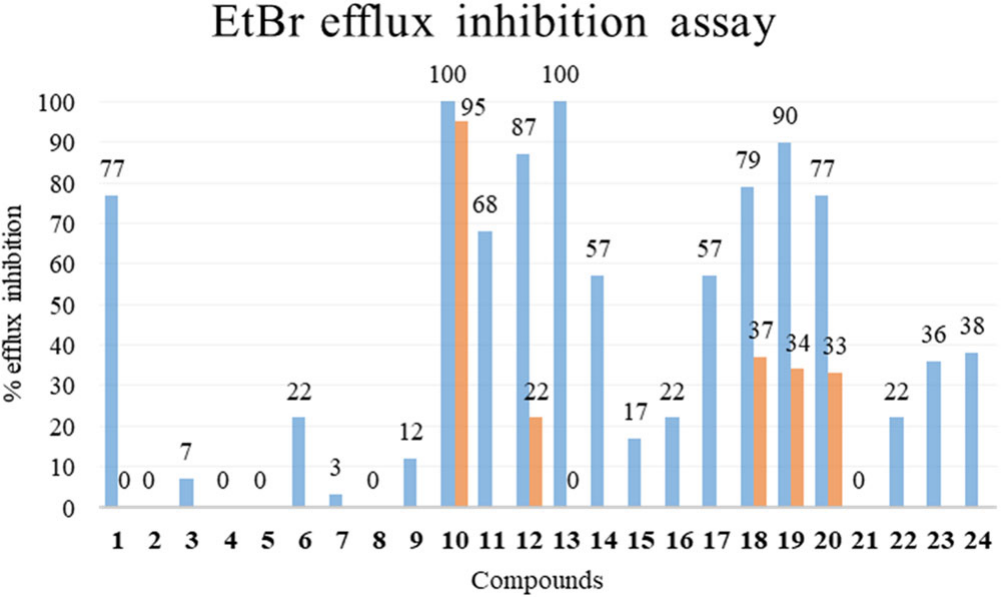
EtBr efflux inhibition (%) on SA‐1199B of compounds **1**–**24** at 50 µM (blue bars) and 10 µM (only for compounds **1**, **10**, **12**, **13,** and **18–20**) (orange bars).

EtBr efflux inhibition assays were conducted using the *S. aureus* SA‐1199B strain, which overexpresses the *norA* gene and has an A116E GrlA substitution. Each compound was tested at 50 µM (see Figure 2). The starting hit compound **1** was used as the internal standard for comparison with the previously reported data.

In the preceding study, in which compound **1** was identified through in silico prediction [18], the previous pharmacophore model indicated that all pyrazolobenzothiazine analogues with a methyl group at the N‐4 position were inactive. Moreover, predictions also suggested that only para‐nitro and para‐ or meta‐fluorine C‐1 phenyl derivatives would have shown NorA inhibition activity [18]. Consequently, all N‐4 methylated analogues and “naked” C‐1 phenyl derivatives were excluded from the testing process. To corroborate this prediction, here, we undertook the resynthesis of the N‐4 methyl analogue (i.e., derivative **2**) and the C‐4′ phenyl des‐nitro analogue (i.e., derivative **3**) of hit compound **1** and evaluated their ability to inhibit the EtBr efflux. As expected, compound **2** was unable to inhibit NorA efflux (Figure 2), demonstrating no impact on EtBr efflux during the assay. This outcome validates the hypothesis that a di‐substituted sulfonamide in the pyrazolobenzothiazine scaffold is detrimental for NorA EP inhibition. Similarly, derivative **3** showed only a modest inhibitory effect on EtBr efflux (7%), suggesting that the para‐nitro group may play a significant role in the NorA inhibition mechanism. Consequently, we undertook an investigation of the C‐3 position of the pyrazolobenzothiazine derivatives, while maintaining the *para*‐nitro phenyl at the C‐1 position and the mono‐substituted sulfonamide in the main scaffold, as both were considered to be essential for the retention of NorA inhibition. Consequently, compound **4** [19] was initially designed and, in order to extend the SAR, its close inverted analogue **5** was also considered. Subsequently, it was postulated that the replacement of the C‐3 para‐chloro phenyl moiety with a methyl group could significantly impact the role of the mono‐substituted sulfonamide of the scaffold. Therefore, synthesis of the two methylated analogues of **4** and **5** (i.e., **6** [19] and **7**, respectively) was also planned. The data obtained from EtBr efflux assays (Figure 2) clearly demonstrated the significant contribution of the C‐3 *para*‐chloro phenyl moiety present in the pyrazolobenzothiazine to the inhibition of NorA. It was observed that, irrespective of the inversion of the endocyclic sulfonamide and/or its methylation, compounds **4–7** were unable to inhibit EtBr efflux in the SA‐1199B strain. The sole exception was the N‐4 methylated derivative **6**, which showed only a modest EtBr efflux inhibition activity (22%). Subsequently, with the aim of introducing flexibility to the pyrazolobenzothiazine scaffold, we proceeded to open the six‐member 1,2‐thiazine ring, thereby obtaining derivative **8**. This derivative was unable to inhibit the EtBr efflux (Figure 2), which indicated that the intact pyrazolobenzothiazine core is essential for the imparting of NorA EP inhibitory activity. In light of the pivotal role of the pyrazolobenzothiazine core, we undertook a comprehensive re‐evaluation of the SAR around the initial hit **1**, which resulted in the complete removal of the N‐1 substituent. This decision was driven by the recognition that, if the N‐1 phenyl moiety was present, it must have the para‐nitro group. However, no information was acquired about the general role of the N‐1 substituent. This led to the synthesis of compound **9**. Concurrently, we postulated that the simultaneous presence of two free NH moieties in compound **9** could facilitate a significant resonance shift within the molecule. Consequently, we undertook re‐planning of the methylation of the N‐4 position, which resulted in the synthesis of compound **10**. Notably, compound **9** inhibited the EtBr efflux by only 12% (Figure 2), whereas the N‐4 methylated analogue **10** showed complete inhibition of EtBr efflux. This highlights two important aspects of SAR: (i) the N‐1 phenyl moiety on the pyrazolobenzothiazine core is not essential to inhibit NorA and (ii) N‐4 methylation is required if the pyrazole moiety is in an unsubstituted form. It is noteworthy that derivative **10** also retained 95% of the EtBr efflux inhibition when tested at 10 µM (Figure 2), suggesting a significant boost in potency with respect to the starting hit **1**, whose EtBr efflux inhibition was completely lost at 10 µM. In view of these encouraging results, we proceeded to design analogous compounds of derivative **10**, in which the para‐chlorine atom on the C‐3 phenyl ring was removed (compound **11**) or replaced with a fluorine atom (compound **12**). Furthermore, we explored the possibility of moving the chlorine atom to the meta position while inserting a fluorine atom in the para position (compound **13**) and considered alkylating the endocyclic sulfonamide with an ethyl group (compound **14**) instead of the methyl group present in **12**. The results indicated that the removal of the chlorine in compound **11** resulted in a reduction in EtBr efflux inhibition to a value below 70% (Figure 2), which we consider to be a crucial threshold for identifying compounds with potent NorA‐inhibitory activity. Conversely, the replacement of the chlorine atom with a fluorine atom yielded compound **12**, which retained the capacity to effectively inhibit EtBr efflux at a concentration of 50 µM. However, its inhibitory capacity was markedly diminished when tested at 10 µM (Figure 2), suggesting that a larger lipophilic atom is more favorable than a smaller one in this position. A direct comparison between the N‐4 methylated compound **12** and the N‐4 ethyl analogue **14** indicated that the methyl group is preferred over the ethyl group in this position. Then, the shift of the chlorine to the meta position and the simultaneous presence of the fluorine in the para position yielded compound **13**, which demonstrated a substantial retention of EtBr efflux inhibition at 50 µM, though this was markedly diminished at 10 µM (Figure 2).

To gain further insight into the role of the sulfonamide substituent, the methyl group of compound **12** was replaced with more polar chains, including hydroxypropyl, *N*‐methylacetamide, and *N*‐methylethylamine chains, resulting in the synthesis of compounds **15–17**, respectively. In all three cases, EtBr efflux inhibition was negatively influenced, indicating that longer and more polar chains than a methyl group are not well tolerated at the N‐4 position of the pyrazolobenzothiazine scaffold.

It is important to note that the presence of an alkylamino chain was found to be essential for significantly enhancing the activity of NorA EPIs in the quinoline and quinazoline series [17, 24]. This observation was corroborated by the pharmacophore model, which indicated that a positive charge was among the four essential features for NorA inhibitors [14]. Accordingly, the plan was to maintain the methyl in the N‐4 position while introducing an alkylamino chain at the N‐1 position (derivative **18**) and at the C‐7 position through an ether bond (derivatives **19** and **20**). The introduction of an aminoalkyl chain in the N‐4 position was not considered a viable option due to the inactivity observed in derivative **17**. It is noteworthy that the introduction of the alkylamino chain yielded pyrazolobenzothiazine analogues with potent EtBr efflux inhibition activity, consistently exceeding 70% (Figure 2). This suggests that both the N‐1 and C‐7 positions may be utilized for the incorporation of this chain. The most promising derivative was compound **19**, which showed a terminal morpholine moiety and demonstrated a 90% reduction in EtBr efflux at 50 µM, while retaining 33% inhibition at 10 µM. To ascertain whether the observed effect was attributable to the presence of the protonable chain and not merely to polarity, we also planned the synthesis of derivative **21**, which proved to be completely inactive. This result confirmed the central role of the protonable charge. Compound **22**, which served as an intermediate in the synthesis of C‐7 O‐alkylated derivatives, was also evaluated. This was done in consideration of the positive impact that was previously observed on introduction of the ‐OMe group on the quinoline core [25]. However, the results indicated that compound **22** did not show EtBr efflux inhibition. This finding indirectly suggests that the SAR observed around the quinoline core cannot be translated to the pyrazolobenzothiazine scaffold. As a final modification, we investigated the importance of the pyrazole moiety by maintaining intact the presence of both the alkylamino chain and methyl on endocyclic sulfonamide. The resulting benzothiazine derivatives **23** and **24** demonstrated a lack of efficacy in reducing EtBr efflux, thereby underscoring the indispensable role of the pyrazole moiety.

#### 2.2.2 MIC Determination and Synergistic Assays With CPX Against SA‐1199B and SA‐1199

The EtBr efflux assays yielded preliminary insights into the pyrazolobenzothiazine core, delineating the optimal regions for identifying potent inhibitors. Nevertheless, the final objective of a NorA inhibitor is to achieve a synergistic effect with an antibacterial agent against bacterial strains that overexpress NorA. In addition, it is essential to observe the synergistic effect at nonantibacterial concentrations of our compounds in order to ascertain their efficacy as EPIs.

To corroborate the findings of the EtBr efflux assays, we undertook an evaluation of all synthesized derivatives with regard to their minimum inhibitory concentration (MIC) and their capacity to exert a synergistic effect with CPX against the SA‐1199B strain (Table 1). After excluding any antibacterial activity that could interfere with the EP inhibition (MIC ≥ 50 µg/mL for all but one pyrazolobenzothiazines—compound **3** ‐ MIC = 25 µg/mL), we proceeded to assay the synergistic activity of CPX in combination with EPIs tested at a fixed concentration of 12.5 µg/mL (Table 1). It is noteworthy that the same trend as that observed in the EtBr efflux inhibition assay was also observed for the synergistic activity of all derivatives, with the exception of compound **3**, which demonstrated a notable synergistic effect, reducing the CPX MIC by eightfold while not inhibiting EtBr efflux. This discrepancy may be attributed to the residual antibacterial activity of compound **3** when tested at the fixed concentration of 12.5 µg/mL, which corresponds to only half of its MIC. However, these data also indirectly suggest that EtBr efflux assays are not affected by the presence of antibacterial compounds. This is exemplified by compound **3**, which, at 50 µM (20.39 µg/mL), has a significant antibacterial effect (Table 1), and yet, this did not translate into EtBr efflux inhibition. This indicates that EtBr is an excellent preliminary assay to discover NorA EPIs.

**Table 1:** EtBr efflux inhibition assay, MIC evaluation, and synergism with CPX against the *Staphylococcus aureus* SA‐1199B strain for compounds **2–24** and the reference compound **1**.

		*Staphylococcus aureus* SA‐1199B			
		Synergism			
Compd.	Structure	MIC (µg/mL)	EPI conc. (µg/mL)	CPX MIC (µg/mL)	N° fold CPX MIC reduction
**2** [18]	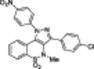	> 50	12.5 (< 1/4 MIC)	10	0
**3** [18]	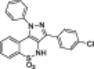	25	**12.5 (1/2 MIC)**	**1.2**	**8**
			**6.25 (1/4 MIC)**	**2.5**	**4**
			**3.12 (1/8 MIC)**	**5**	**2**
**4** [19]	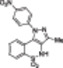	> 50	12.5 (< 1/4 MIC)	10	0
**5**	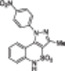	> 50	12.5 (< 1/4 MIC)	10	0
**6** [19]	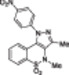	> 50	12.5 (< 1/4 MIC)	10	0
**7**	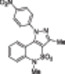	> 50	12.5 (< 1/4 MIC)	10	0
**8**	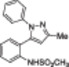	> 50	12.5 (< 1/4 MIC)	5	2
**9**	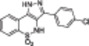	> 50	12.5 (< 1/4 MIC)	5	2
**10**	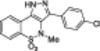	> 50	**12.5 (**< **1/4 MIC)**	**2.5**	**4**
			**6.25 (**< **1/8 MIC)**	**1.2**	**8**
			**3.12 (**< **1/16 MIC)**	**2.5**	**4**
**11**	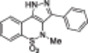	> 50	12.5 (< 1/4 MIC)	10	0
**12**	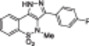	> 50	**12.5 (**< **1/4 MIC)**	**2.5**	**4**
			**6.25 (**< **1/8 MIC)**	**2.5**	**4**
			**3.12 (**< **1/16 MIC)**	**5**	**2**
**13**	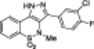	> 50	**12.5 (**< **1/4 MIC)**	**2.5**	**4**
			**6.25 (**< **1/8 MIC)**	**2.5**	**4**
			**3.12 (**< **1/16 MIC)**	**5**	**2**
**14**	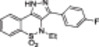	> 50	12.5 (< 1/4 MIC)	5	2
**15**	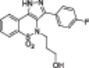	> 50	12.5 (< 1/4 MIC)	5	2
**16**	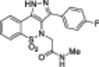	> 50	12.5 (< 1/4 MIC)	5	2
**17**	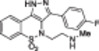	> 50	12.5 (< 1/4 MIC)	10	0
**18**	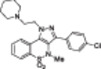	> 50	**12.5 (**< **1/4 MIC)**	**2.5**	**4**
			**6.25 (**< **1/8 MIC)**	**5**	**2**
**19**	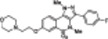	> 50	**12.5 (**< **1/4 MIC)**	**2.5**	**4**
			**6.25 (**< **1/8 MIC)**	**2.5**	**4**
			**3.12 (**< **1/16 MIC)**	**5**	**2**
**20**	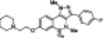	> 50	**12.5 (**< **1/4 MIC)**	**1.2**	**8**
			**6.25 (**< **1/8 MIC)**	**5**	**2**
**21**	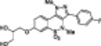	> 50	12.5 (< 1/4 MIC)	10	0
**22**	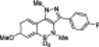	> 50	12.5 (< 1/4 MIC)	5	2
**23**	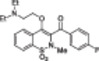	> 50	12.5 (< 1/4 MIC)	5	2
**24**	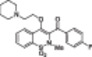	> 50	12.5 (< 1/4 MIC)	5	2
**1** [18]	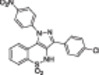	50	12.5 (1/4 MIC)	0.63	16
			6.25 (1/8 MIC)	1.25	8
			3.12 (1/16 MIC)	5	2
CPX		10	nt	nt	nt

*Note:* The values in bold are those of compounds that demonstrated a fourfold reduction in CPX MIC at 12.5 µg/mL and thus were evaluated for synergistic activity at lower concentrations with CPX.

Abbreviation: NT, not tested.

Based on the results obtained from the synergistic assays of compounds at 12.5 µg/mL in combination with CPX, the most promising derivatives (**3**, **10**, **12**, **13**, **18**, **19**, and **20**) were selected for further evaluation of their synergistic effect at lower concentrations (6.25 and 3.12 µg/mL) against the SA‐1199B strain (Table 1). For purposes of comparison, compound **1** was included as a reference. It is noteworthy that, at a concentration of 6.25 µg/mL, compounds **3**, **10**, **12**, **13**, and **19** demonstrated a considerable synergistic effect with CPX, resulting in a reduction of its MIC by at least fourfold. However, in accordance with the loss of EtBr efflux inhibition at a lower concentration of 3.12 µg/mL, none of the compounds could synergize with CPX, with the exception of derivative **10**.

Indeed, pyrazolobenzothiazine derivative **10** at 3.13 µg/mL still demonstrated the ability to reduce the CPX MIC by fourfold, which was in accordance with the ability of **10** to retain EtBr efflux inhibition by 95% also at 10 µM (3.46 µg/mL).

At this stage, compounds **3**, **10**, **12**, **13**, **18**, **19**, and **20** were also evaluated individually (MIC assessment) and in combination at a concentration of 3.13 µg/mL with CPX (synergistic assays) against the wild‐type *S. aureus* SA‐1199 strain. The compounds showed MIC values exceeding 50 µg/mL, and as expected, they did not show any synergistic effect with CPX. This finding aligns with their mechanism of action, which involves the inhibition of NorA, which is only basally expressed in the wild‐type SA‐1199 strain.

### 2.3 Checkerboard Assays of the Combination EPIs/CPX Against *S. aureus* K1902 and K2378

To further confirm the involvement of NorA inhibition in the mechanism of action of compounds **10** and **19** (the most promising derivatives in terms of EtBr efflux inhibition and synergistic activity with CPX against SA‐1199B), MIC evaluation and checkerboard assays were conducted against both the *S. aureus* SA‐K1902 (NorA‐knock out, Δ*norA*) and SA‐K2378 (overexpressing *norA* gene) strains (Table 2). Compound **3** was included in the analysis, since it demonstrated a notable synergistic effect with CPX against SA‐1199B, despite occurring at a concentration that was in close proximity to its MIC value. Checkerboard assays were performed using a scalar concentration of EPIs (ranging from ¼ MIC to lower concentrations) in combination with a scalar concentration of CPX. As expected, none of the compounds demonstrated a notable synergistic effect with CPX against SA‐K1902 at all tested concentrations (Table 2), reinforcing the observation that, in the absence of NorA, compounds could not show a synergistic effect with CPX. Conversely, interesting results were obtained against SA‐K2378, which overexpresses the *norA* gene (Table 2). Derivatives **3** and **10** demonstrated the capacity to reduce the CPX MIC by fourfold up to a concentration of 0.8 µg/mL (equivalent to 1/32 and 1/16 MIC, respectively). Derivative **19** yielded even more promising results, demonstrating the capacity to synergize with CPX at the lower tested concentration of 0.2 µg/mL (1/128 MIC), reducing the CPX MIC by fourfold. Taken together, these results demonstrate that pyrazolobenzothiazine derivatives show a potent synergistic effect with CPX only against the SA‐K2378 strain that overexpresses the *norA* gene. This corroborates the hypothesis that their activity is strictly related to NorA EP inhibition.

**Table 2:** MIC evaluation and checkerboard assay against *S. aureus* SA‐K1902 and SA‐K2378 strains for compounds **3, 10,** and **19** and the reference compound **1**.

	SA‐K1902 (*norA*‐)				SA‐K2378 (*norA* ++)			
Compd.	MIC (µg/mL)	Conc of EPI (µg/mL)	CPX MIC (µg/mL)	N° CPX MIC reduction	MIC (µg/mL)	Conc of EPI (µg/mL)	CPX MIC (µg/mL)	N° CPX MIC reduction
**3**	> 50	12.5	0.08	2	25	**6.25**	**0.08**	**16**
		6.25	0.08	2		**3.13**	**0.16**	**8**
		3.13	0.08	2		**1.6**	**0.16**	**8**
		1.6	0.08	2		**0.8**	**0.3**	**4**
		0.8	0.08	2		0.4	0.6	2
		0.4	0.08	2		0.2	0.6	2
		0.2	0.08	2				
**10**	12.5	3.13	0.08	2	12.5	**3.13**	**0.08**	**16**
		1.6	0.08	2		**1.6**	**0.16**	**8**
		0.8	0.08	2		**0.8**	**0.3**	**4**
		0.4	0.08	2		0.4	0.6	2
		0.2	0.08	2		0.2	0.6	2
**19**	> 50	12.5	0.15	0	25			
		6.25	0.08	2		**6.25**	**0.16**	**8**
		3.13	0.08	2		**3.13**	**0.3**	**4**
		1.6	0.08	2		**1.6**	**0.3**	**4**
		0.8	0.08	2		**0.8**	**0.3**	**4**
		0.4	0.08	2		**0.4**	**0.3**	**4**
		0.2	0.08	2		**0.2**	**0.3**	**4**
**1** [18]	12.5	3.13	0.15	0	12.5	**3.13**	**0.3**	**4**
		1.6	0.15	0		**1.6**	**0.3**	**4**
		0.8	0.15	0		0.8	0.6	2
CPX	—	—	0.15	—	—	—	1.25	—

*Note:* The bold font indicates the concentrations of the compounds that resulted in a minimum fourfold decrease in the CPX MIC.

The only remaining uncertainty concerns compound **3**. While it demonstrated a synergistic effect with CPX only against the *norA*‐overexpressing strain (SA‐K2378—Table 2), initial observations indicated no inhibition of EtBr efflux against SA‐1199B (Table 1). Furthermore, against this same strain, a synergistic effect with CPX was observed at concentrations approaching its MIC (Table 1). At this stage, we reserve the right to consider a particular mechanism for compound **3**, which may differ from that of the classic NorA inhibitors.

### 2.4 Inhibition of Biofilm Production

**Figure 3:**
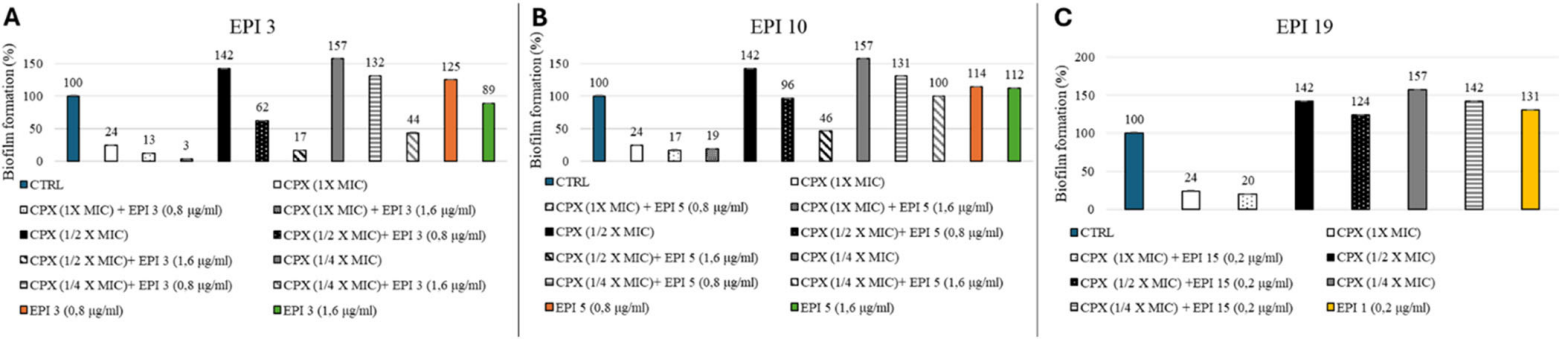
The % of biofilm formation of *Staphylococcus aureus* SA‐K2378, compared with the control (100%), during growth in the presence of CPX and/or EPIs **3**, (A) **10** (B), and **19** (C). The biofilm formation during growth in free agent medium was the control. Data represent the mean ± SD of an experiment conducted in biological triplicate.

The formation of biofilms is a significant contributor to the development of AMR [1, 26, 27]. This is due to the fact that biofilms provide a protective environment for bacteria, thereby rendering them more resilient to the effects of common antibacterial agents [28, 29, 30]. Over the years, several investigations have been conducted to study the role of EPs in the various stages of microbial biofilm formation [31, 32]. However, a definitive answer regarding the potential role of EPs in this microbial pathway remains elusive. While some studies have reported a negative effect of certain EPs on biofilm formation, others have highlighted the promising role of EPIs in limiting biofilm formation and enhancing the efficacy of antibiotics under biofilm conditions [33]. Accordingly, the variation of biofilm production in the presence of CPX alone and in the presence of EPIs (**3**, **10**, and **19**) was evaluated in SA‐K2378 (*norA* ++). In particular, the biofilm in the SA‐K2378 was quantified following treatment with CPX alone (at MIC = 1.25 µg/mL, ½ MIC = 0.6 µg/mL, or ¼ MIC = 0.3 µg/mL), with EPIs alone at different concentrations, or with CPX combined with EPIs, as illustrated in Figure 3. The concentrations of EPIs used in these experiments were 0.8 and 1.6 µg/mL for compounds **3** and **10**, respectively, and 0.2 µg/mL for compound **19**. The selection of these concentrations was based on the observation of the respective synergistic effects with CPX observed in the previous checkerboard assays.

In general, the use of CPX at the MIC value (1.25 µg/mL) resulted in a notable reduction in biofilm formation in comparison to the untreated control. However, it should be noted that the inhibition of biofilm formation never reached 100%. At lower concentrations of CPX, the formation of biofilms was instead observed to increase in comparison to the control. On the other hand, it is noteworthy that the combinations of EPIs with CPX at subinhibitory concentrations translated into a positive reduction in biofilm formation. The combination of compounds **3** and **10** at 1.6 µg/mL with CPX at ½ MIC yielded the most favorable outcomes, with a reduction in biofilm formation of 83% and 54%, respectively. Notably, CPX alone at this concentration resulted in an increase of 42% in biofilm formation, indicating that a combination of EPIs and CPX has the potential to counteract biofilm formation. Notably, compound **3** at 1.6 µg/mL showed a positive synergistic effect also when combined with CPX tested at ¼ of its MIC, whereas compound **10** at the same concentration only resulted in an untreated control‐like condition, thereby limiting the production of biofilm in response to CPX. In contrast, no positive effects were observed when compound **19** was combined with CPX. This lack of synergism may be attributed to the lower concentration of compound **19** used in comparison to other compounds. Furthermore, it is notable that when EPIs **3**, **10**, and **19** were tested individually, a slight increase in biofilm production was observed (with the exception of **3** at 1.6 µg/mL), although this was less pronounced than that observed for CPX at subinhibitory concentrations. This phenomenon can be considered as the bacterium's response to the presence of NorA EPIs, which results in increased biofilm production. It is possible that the bacterium is able to circumvent the inhibitory effects of NorA, despite the fact that NorA is known to play a role in biofilm formation [34, 35, 36]. One potential mechanism for this is the overexpression of different EPs. However, given that EPIs should not be administered alone, the effect to be considered is that in combination with CPX, which significantly led to a reduction in biofilm formation, thereby underscoring the potential of this combination. As compounds are NorA EPIs, no synergistic effects were observed in combination with CPX in the biofilm formation of SA‐K1902 (Δ*norA*) compared with the untreated control (data not shown).

### 2.5 Cytotoxicity Assays

The reference compound (**1**) and pyrazolobenzothiazine derivatives **3** and **10** were subjected to an MTT assay to assess their cytotoxicity against two human cell lines (A549 and MCF7) (Table 3). In general, the compounds showed no significant cytotoxic effects against both cell lines at a concentration of 12.5 µg/mL. It is noteworthy that this concentration is higher than that required to synergize with CPX against the resistant strain SA‐1199B. The lowest degree of cytotoxicity was observed for compound **3**, with a CC_50_ value exceeding 100 µg/mL against both cell lines. In contrast, compound **10** showed a slightly higher degree of cytotoxicity, with a 50% reduction in cell viability observed at concentrations close to 25 µg/mL in both cell lines.

**Table 3:** Cytotoxicity evaluation of EPIs on MCF7 and A549 cell lines.

	Cytotoxicity (% cell viability)							
	Concentration (μg/mL)							
	A549				MCF7			
Compounds	100	50	25	12.5	100	50	25	12.5
**1**	8 ± 1	32 ± 5	76 ± 1	90 ± 16	43 ± 3	50 ± 3	71 ± 3	81 ± 6
**3**	34 ± 10	101 ± 24	99 ± 20	94 ± 24	66 ± 7	88 ± 4	84 ± 4	97 ± 10
**10**	7 ± 2	7 ± 0	45 ± 3	97 ± 2	6 ± 0	7 ± 1	26 ± 2	66 ± 5

## 3 Conclusions

Microbial EPs play a pivotal role in the emergence of nonspecific resistance to commonly used antimicrobials. Consequently, the inhibition of EPs represents a promising strategy to counteract AMR through the use of nonantibiotic molecules in combination with antibiotic substrates. Of the various EPs studied in *S. aureus*, NorA is the best characterized and it is responsible for extruding a range of harmful agents, including fluoroquinolones. In this study, we sought to expand the range of molecular classes showing NorA inhibition activity starting from the hit compound **1**, which had previously been identified by us as a NorA EPI. Initially, we conducted a comprehensive investigation into the SAR of the pyrazolobenzothiazine class, which has been less extensively studied, with the objective of gaining comprehensive insights into the capacity of these novel analogues to inhibit EtBr efflux in SA‐1199B. This approach led to the identification of new pyrazolobenzothiazine compounds that demonstrated significant synergistic activity with CPX against SA‐1199B. Furthermore, a linear correlation was identified between EtBr efflux inhibition and synergistic activity. Only compound **3** was capable of showing strong synergistic activity with CPX, and yet, it lacked the capacity to inhibit EtBr. This is likely attributable to its relatively low MIC value, which renders it an unpromising EPI or a different mechanism of action from the canonical NorA inhibition. However, compound **3** and the most promising EPIs **10** and **19** were evaluated in combination with CPX against the pair strains SA‐K2378 (overexpressing *norA*) and SA‐K1902 (Δ*norA*), indicating that the mechanism of action is dependent on the presence of the NorA EP. It is noteworthy that the synergistic effect of the most potent NorA EPIs with CPX also resulted in a reduction in biofilm production by SA‐K2378.

In conclusion, new insights around the SAR of the pyrazolobenzothiazine derivatives were added and novel analogues were identified as promising NorA EPIs. New results will yield valuable data for the refinement of the 3D pharmacophore models for NorA EPIs. Indeed, this study confirmed the suitability of EtBr efflux assays for the rapid identification of NorA inhibitors. Consequently, the acquisition of additional data from this assay on new classes of analogues will be beneficial for the improvement of previously developed pharmacophore models for NorA inhibitors, thus extending the limited number of classes of derivatives.

## 4 Experimental

### 4.1 Chemistry

#### 4.1.1 General

All starting materials, reagents, and solvents were purchased from common commercial suppliers and were used as such, unless otherwise indicated. Organic solutions were dried in anhydrous Na_2_SO_4_ and concentrated with a rotary evaporator at low pressure. All reactions were routinely checked by thin‐layer chromatography (TLC) on silica gel 60_F254_ (Merck) and visualized by using UV or iodine. Flash chromatography separations were carried out on Merck silica gel 60 (mesh 230–400). Yields were of purified products and were not optimized. Bruker AC‐200 was used to record ^1^H NMR spectra at 200 MHz. Bruker Avance DRX‐400 and DRX‐600 (Bruker Corporation, Massachusetts, USA) were used to record ^1^H NMR and ^13^C NMR spectra at 400 and 101 MHz, and at 600 and 150 MHz, respectively. Chemical shifts are given in ppm (δ) relative to TMS. Spectra were acquired at 298 K, unless otherwise indicated. Data processing was performed using standard Bruker software TopSpin (Vers. 4.1.4) and the spectral data are consistent with the assigned structures. Detection mass was based on electrospray ionization (ESI) in positive polarity using an Agilent 1290 Infinity System equipped with an MS detector Agilent 6540UHD Accurate Mass Q‐TOF.

Compounds **15**, **16**, **17,** and **22** were ≥ 95% pure, as determined by LC/MS using an Agilent 1290 Infinity System machine equipped with a DAD detector from 190 to 640 nm. The purity was revealed at 254 nm using a Phenomenex AERIS Widepore C4, 4.6 mm, 100 mm (6.6 lm) with flow rate: 0.5 mL/min; acquisition time: 10 min; gradient: acetonitrile in water containing 0.1% of formic acid (0%–100% in 10 min); and oven temperature, 30 C. Peak retention time (tR) is given in minutes.

Compounds **5**, **7**, **8**–**14**, **18**–**21**, **23**, and **24** were ≥ 95% pure, as determined by HPLC analysis using the Jasco LC‐4000 instrument equipped with a UV–Visible Diode Array Jasco MD‐4015 and a C18 Column (Gemini, Phenomenex) 110 Å, 3 μm, 100 × 2 mm. The flow rate was 0.50 mL/min. Method A: acquisition time = 10 min with a gradient consisting of acetonitrile (ACN) and water containing 0.1% formic acid, with a linear increase of ACN from 20% to 100% over 10 min. Method B: acquisition time = 15 min with a gradient consisting of ACN and water containing 0.1% formic acid, with a linear increase of ACN from 20% to 100% over 15 min. The purity was revealed at 254 nm, and the methods used have been specified for each compound. Chromatograms were analyzed using ChromNAV 2.0 Chromatography Data System software, and the peak retention time (tR) is given in minutes.

In the ^13^C NMR experiments, we encountered some difficulties in observing the quaternary carbon signals in pyrazolobenzothiazine (tricyclic) compounds with the aromatic moiety at the C‐3 position. No improvement was observed when the temperature was increased up to 323 K. For one compound (compound **10**), the experimental conditions were varied, and all the carbon signals of the molecule were observed, although not optimally. However, the experiment required the use of 15 mg of compound, a delay time of 4 s, and 8000 scans (12 h). Consequently, these less common conditions were not repeated for all compounds, as their structures had already been confirmed by ^1^H NMR and HRMS experiments.

Target compounds **2**, **3**, **4**, and **6** were re‐synthesized according to the procedures already reported by us previously [18, 19]. The ^1^H NMR and ^13^C NMR spectra are consistent with those that have been previously reported.

The InChI codes of the investigated compounds, together with some biological activity data, are provided as Supporting information.

#### 4.1.2 General Procedure (A) for the Synthesis of Compounds **34–38**

Under a N_2_ atmosphere, to a solution of compound **32** or **33** (2.0 eq) in dry DMSO (15 mL), proper acetophenone (1.0 eq) was added dropwise and the reaction was stirred at 70°C for 40 min–3 h. The mixture was poured into ice and water and the formed precipitate was filtered to obtain target compounds **34**–**38**.

#### 4.1.3 General Procedure (B) for the Synthesis of Compounds **39–43**

To a solution of metallic Na (2.5 eq) in EtOH (15 mLx mmol), compounds **34–38** (1 eq) were added. The mixture was warmed at 60°C for 30 min–3 h. The mixture was poured into ice water, modifying the pH to 5 with 2 N HCl to obtain a precipitate that was filtered, yielding target compounds **39**–**43**.

#### 4.1.4 General Procedure (C) for the Synthesis of Compounds **44–49** and **51**

To a mixture of NaOH/EtOH (1:4), compounds **39–43** (1.0 eq) were added and after 30 min, MeI or EtI or bromo ethylacetate (3.0 eq) was added dropwise. The reaction was stirred for 1 h—overnight at room temperature. It was poured into ice water, modifying the pH to 4 with 2 N HCl to obtain a precipitate that was filtered, yielding compounds **44–49** and **51**.

#### 4.1.5 General Procedure (D) for the Synthesis of Compounds **9**, **10**, **11**, **13**, **14**, **15**, **16,** and **22**

Compounds **39**, **44**–**50**, and **53** (1.0 eq) and hydrazine monohydrate or methyl hydrazine (1.0 eq) were reacted, and the reaction was stirred at 65°C for 50 min–5 h. The mixture was poured into ice water, modifying the pH to 3 with 2 N HCl to obtain a precipitate that was filtered. After purification by a flash chromatography column, compounds **9–16** and **22** were obtained.

#### 4.1.6 Characterization of the Synthesized Compounds

Methyl 2‐{[(2‐oxopropyl)sulfonyl]amino}benzoate (**27**): Under a N_2_ atmosphere, to a solution of **26** (4.89 g, 31.25 mmol) in dry benzene (20 mL), methyl anthranilate (4.04 mL, 31.25 mmol) and Et_3_N (6.56 mL, 46.87 mmol) were added at 0°C. The reaction mixture was stirred at 90°C for 1 h and 30 min. The mixture was poured into ice and water and extracted with EtOAc. The organic layer was washed with brine, dried with Na_2_SO_4_, and the solvent was concentrated under vacuum to obtain a yellow solid. After purification by flash column chromatography eluting with Pet/EtOAc 90/10, intermediate **27** was obtained as a yellow solid (23% yield). ^1^HNMR (CDCl_3_, 200 MHz) *δ*_H_: 2.33 (3H, s, NHSO_2_CH_2_CO*CH*_*3*_), 3.90 (3H, s, OCH_3_), 4.05 (2H, s, NHSO_2_ *CH*_*2*_COCH_3_), 7.10 (1H, dt, *J* = 1.2 and 7.4 Hz, Ar‐H), 7.49 (1H, dt, *J* = 1.6 and 7.3 Hz, Ar‐H), 7.69 (1H, dd, *J* = 1.1 and 8.4 Hz, Ar‐H), 7.80 (1H, dd, *J* = 1.6 and 7.9 Hz), 10.61 (1H, bs, NH).

1‐(4‐Hydroxy‐2,2‐dioxido‐1*H*‐2,1‐benzothiazin‐3‐yl)ethenone (**28**): Compound **27** (1.00 g, 3.69 mmol) was added to a solution of sodium ethoxide (1.00 g, 14.76 mmol) in EtOH (15 mL). The mixture was warmed at 70°C and after 40 min, the reaction was completed. The mixture was poured into ice water, modifying the pH to 5 with 2 N HCl. The formed precipitate was filtered to obtain intermediate **28** as a white solid (68% yield). ^1^HNMR (DMSO‐*d*_*6*_, 200 MHz) *δ*_H_: 2.68 (3H, s, COCH_3_), 7.10 (1H, dd, *J* = 1.1 and 8.4 Hz, Ar‐H), 7.49 (1H, dt, *J* = 1.6 and 7.3 Hz, Ar‐H), 7.69(1H, dt, *J* = 1.2 and 7.4 Hz, Ar‐H), 7.80 (1H, dd, *J* = 1.6 and 7.9 Hz), 12.21 (1H, bs, NH), 16.41 (1H, bs, OH).

3‐Methyl‐5‐(2‐nitrophenyl)‐1‐phenyl‐1*H*‐pyrazole (**30**): To a solution of compound **29** (5.00 g, 24.15 mmol) in EtOH (150 mL), phenyl hydrazine hydrochloride (3.84 g, 26.56 mmol) was added, and the reaction was stirred at reflux for 12 h. The mixture was poured into ice water to obtain a precipitate that was filtered. After purification by chromatography column eluting with CH_2_Cl_2_/Et_2_O 99/1, compound **30** was obtained as a white solid (31% yield). ^1^HNMR (CDCl_3_, 200 MHz) *δ*_H_: 2.49 (3H, s, CH_3_), 6.34 (1H, s, Ar‐H), 7.24–7.32 (5H, m, Ar‐H), 7.42 (1H, dd, *J* = 1.6 and 7.4 Hz, Ar‐H), 7.56–7.65 (2H, m, Ar‐H), 7.94 (1H, dd, *J* = 1.7 and 7.7 Hz, Ar‐H).

2‐(3‐Methyl‐1‐phenyl‐1*H*‐pyrazol‐5‐yl)aniline (**31**): To a solution of **30** (2.0 g, 7.17 mmol) in EtOH (20 mL), Raney‐Ni (10 mmol %) was added and then hydrazine monohydrate (10 mL, 206.4 mmol) was added dropwise. The mixture was stirred at room temperature for 1 h and 30 min. After filtration by celite, the filtrate was concentrated under vacuum and then it was poured into ice and water to obtain a yellow precipitate that was filtered. After purification by flash chromatography eluting with CHCl_3_/MeOH 98/2, compound **31** was obtained as a yellow solid (90% yield).^1^HNMR (CDCl_3_, 200 MHz) *δ*_H_: 2.42 (3H, s, CH_3_), 3.80 (2H, s, NH_2_), 6.32 (1H, s, Ar‐H), 6.68–6.72 (2H, m, Ar‐H), 6.93 (1H, d, *J* = 8.0 Hz, Ar‐H), 7.15–7.33 (6H, m, Ar‐H).

(4‐Chlorophenyl)(4‐hydroxy‐1,1‐dioxido‐2*H*‐1,2‐benzothiazin‐3‐yl)methanone (**39**): General procedure (B): starting from **34** (time = 30 min), compound **39** was obtained as a yellow solid (1.00 g, 68% yield). ^1^HNMR (DMSO‐*d*_*6*_, 400 MHz) *δ*_H_: 7.66 (2H, d, *J* = 8.6 Hz, Ar‐H), 7.87–7.93 (3H, m, Ar‐H), 7.98 (2H, d, *J* = 8.6 Hz, Ar‐H), 8.14–8.17 (1H, m, Ar‐H), 9.89 (1H, bs, NH), 15.32 (1H, s, OH).

(4‐Hydroxy‐1,1‐dioxido‐2H‐1,2‐benzothiazin‐3‐yl)(phenyl)methanone (**40**): General procedure (B): starting from **35** (time = 2 h), compound **40** was obtained as a yellow solid (1.38 g, 86% yield). ^1^HNMR (DMSO‐*d*_*6*_, 400 MHz) *δ*_H_: 7.60–7.71 (3H, m, Ar‐H), 7.92–7.97 (3H, m, Ar‐H), 8.02 (2H, d, *J* = 7.3 Hz, Ar‐H), 8.20–8.22 (1H, m, Ar‐H), 9.92 (1H, s, NH), 15.29 (1H, bs, OH).

(4‐Fluorophenyl)(4‐hydroxy‐1,1‐dioxido‐2*H*‐1,2‐benzothiazin‐3‐yl)methanone (**41**): General procedure (B): starting from **36** (time = 2 h), compound **41** was obtained as a yellow solid (1.23 g, 64% yield). ^1^HNMR (DMSO‐*d*_*6*_, 200 MHz) δ_H_: 7.42 (2H, t, *J* = 8.9 Hz, Ar‐H), 7.87–7.89 (3H, m, Ar‐H), 8.01‐8.15 (3H, m, Ar‐H), 9.90 (1H, s, NH), 15.30 (1H, bs, OH).

(3‐Chloro‐4‐fluorophenyl)(4‐hydroxy‐1,1‐dioxido‐2*H*‐1,2‐benzothiazin‐3‐yl)methanone (**42**): General procedure (B): starting from **37** (time = 20 min), compound **42** was obtained as a yellow solid (1.13 g, 77% yield). ^1^HNMR (DMSO‐*d*_*6*_, 200 MHz) *δ*_H_: 7.55 (1H, s, Ar‐H), 7.75–8.00 (4H, m, Ar‐H), 8.09–8.19 (2H, m, Ar‐H), 9.88 (1H, bs, NH), 15.27 (1H, bs, OH).

(4‐Fluorophenyl)(4‐hydroxy‐7‐methoxy‐1,1‐dioxido‐2*H*‐1,2‐benzothiazin‐3‐yl)methanone (**43**): General procedure (B): starting from **38** (time = 30 min), compound **43** was obtained as a yellow solid (1.00 g, 68% yield). ^1^HNMR (DMSO‐*d*_*6*_, 200 MHz) *δ*_H_: 3.99 (3H, s, OCH_3_), 7.36–7.52 (4H, m, Ar‐H), 8.07–8.23 (3H, m, Ar‐H), 9.91 (1H, s, NH), 15.33 (1H, bs, OH).

(4‐Chlorophenyl)(4‐hydroxy‐2‐methyl‐1,1‐dioxido‐2*H*‐1,2‐benzothiazin‐3‐yl)methanone (**44**): General procedure (C): starting from **39** and using MeI (time = 12 h), compound **44** was obtained as a yellow solid (1.03 g, 56% yield). ^1^HNMR (CDCl_3_, 200 MHz) *δ*_H_: 2.64 (3H, s, NCH_3_), 7.46 (2H, d, *J* = 8.7 Hz, Ar‐H), 7.74–7.80 (2H, m, Ar‐H), 7.86–7.91 (1H, m, Ar‐H), 8.06–8.18 (3H, m, Ar‐H), 15.48 (1H, bs, OH).

(4‐Hydroxy‐2‐methyl‐1,1‐dioxido‐3,4‐dihydro‐2*H*‐1,2‐benzothiazin‐3‐yl)(phenyl)methanone (**45**): General procedure (C): starting from **40** and using MeI (time = 5 h), compound **45** was obtained as a yellow solid (0.90 g, 68% yield). ^1^HNMR (DMSO‐*d*_*6*_, 400 MHz) *δ*_H_: 2.64 (3H, s, NCH_3_), 7.62–7.66 (2H, m, Ar‐H), 7.7–7.74 (1H, m, Ar‐H), 7.99–8.00 (3H, m, Ar‐H), 8.07 (2H, d, *J* = 7.2 Hz, Ar‐H), 8.20–8.23 (1H, m, Ar‐H), 15.52 (1H, bs, OH).

(4‐Fluorophenyl)(4‐hydroxy‐2‐methyl‐1,1‐dioxido‐2*H*‐1,2‐benzothiazin‐3‐yl)methanone (**46**): General procedure (C): starting from **41** and using MeI (time = 3 h), compound **46** was obtained as a yellow solid (1.50 g, 82% yield). ^1^HNMR (DMSO‐*d*_*6*_, 200 MHz) *δ*_H_: 2.66 (3H, s, NCH_3_), 7.50 (2H, t, *J* = 8.9 Hz, Ar‐H), 7.96–8.06 (3H, m, Ar‐H), 8.13–8.24 (3H, m, Ar‐H), 15.51 (1H, bs, OH).

(3‐Chloro‐4‐fluorophenyl)(4‐hydroxy‐2‐methyl‐1,1‐dioxido‐2H‐benzo[e][1,2]thiazin‐3‐yl)methanone (**47**): General procedure (C): starting from **42** and using MeI (time = 5 h), compound **47** was obtained as a yellow solid (1.19 g, 66% yield). ^1^HNMR (DMSO‐*d*_*6*_, 200 MHz) *δ*_H_: 2.59 (3H, s, NCH_3_), 7.64 (1H, dt, *J* = 2.2 and 9.0 Hz, Ar‐H), 7.84–8.14 (6H, Ar‐H), 15.56 (1H, bs, OH).

(4‐Fluorophenyl)(4‐hydroxy‐7‐methoxy‐2‐methyl‐1,1‐dioxido‐2*H*‐1,2‐benzothiazin‐3‐yl)methanone (**48**): General procedure (C): starting from **43** and using MeI (time = 2 h), compound **48** was obtained as a yellow solid (1.87 g, 80% yield). ^1^HNMR (DMSO‐*d*_*6*_, 200 MHz) *δ*_H_: 2.68 (3H, s, NCH_3_), 3.94 (3H, s, OCH_3_), 7.49–7.54 (4H, m, Ar‐H), 8.12–8.20 (3H, m, Ar‐H), 15.55 (1H, bs, OH).

(2‐Ethyl‐4‐hydroxy‐1,1‐dioxido‐2*H*‐1,2‐benzothiazin‐3‐yl)(4‐fluorophenyl)methanone (**49**): General procedure (C): starting from **41** and using EtI (time = 3 h, compound **49** was obtained as a yellow solid (0.50 g, 50% yield). ^1^HNMR (DMSO‐*d*_*6*_, 400 MHz) *δ*_H_: 0.37 (3H, t, *J* = 7.04 Hz NCH_2_ *CH*_*3*_), 3.09 (2H, q, *J* = 6.9 Hz, N*CH*_*2*_CH_3_), 7.41–7.46 (2H, m, Ar‐H), 7.91–7.93 (3H, m, Ar‐H), 8.07–8.10 (2H, m, Ar‐H), 8.14–8.15 (1H, m, Ar‐H), 15.06 (1H, s, OH).

(4‐Fluorophenyl)[4‐hydroxy‐2‐(3‐hydroxypropyl)‐1,1‐dioxido‐2*H*‐1,2‐benzothiazin‐3‐yl]methanone (**50**): In a 3‐neck round‐bottom flask, under N_2_ conditions, to a suspension of 60% NaH (0.05 g, 1.41 mmol) in dry DMF (20 mL), compound **41** was added dropwise. After 30 min of stirring, 3‐chloropropan‐1‐ol (0.15 mL, 1.8 mmol) was added and the reaction was stirred at room temperature for 24 h. The mixture was poured into ice and water, modifying the pH to 4 with 2 N HCl to obtain a precipitate that was filtered. Compound **50** was obtained as a yellow solid (15% yield). ^1^HNMR (Acetone‐*d*_*6*_, 400 MHz) *δ*_H_: 1.16–1.20 (2H, m, NCH_2_ *CH*_*2*_CH_2_OH), 3.13–3.16 (2H, m, N*CH*_*2*_CH_2_CH_2_OH), 3.21–3.24 (2H, m, NCH_*2*_CH_2_ *CH*_*2*_OH), 3.34 (1H, t, *J* = 4.9 Hz, NCH_*2*_CH_2_CH_2_O*H*), 7.37 (2H, t, *J* = 6.8 Hz, Ar‐H), 7.92–7.99 (3H, m, Ar‐H), 8.16–8.25 (3H, m, Ar‐H), 15.69 (1H, s, OH).

Ethyl [3‐(4‐fluorophenyl)‐5,5‐dioxidopyrazolo[4,3‐*c*][1,2]benzothiazin‐4(1*H*)‐yl]acetate (**51**): General procedure (C): starting from **41** and using ethyl bromo acetate (time = 1 h), compound **51** was obtained as a yellow solid (0.50 g, 44% yield). ^1^HNMR (DMSO‐*d*_*6*_, 400 MHz) *δ*_H_: 0.78 (3H, t, *J* = 7.1 Hz, OCH_2_ *CH*_*3*_), 3.67 (2H, q, *J* = 7.1 Hz, O*CH*_*2*_CH_3_), 3.91 (2H, s, N*CH*_*2*_), 7.43 (2H, t, *J* = 7.2 Hz, Ar‐H), 7.87–7.89 (3H, m, Ar‐H), 8.00–8.14 (3H, m, Ar‐H), 14.86 (1H, bs, OH).

[3‐(4‐Fluorophenyl)‐5,5‐dioxidopyrazolo[4,3‐*c*][1,2]benzothiazin‐4(1*H*)‐yl]acetic acid (**52**): To a solution of **51** (0.78 g, 1.91 mmol) in THF (48 mL), 1 N LiOH aqueous sol. (9.6 mL, 9.59 mmol) was added and the mixture was stirred at room temperature for 1 h. Then, the reaction was concentrated under reduced pressure and the crude was poured into ice and water to obtain a precipitate that was filtered. Compound **52** was obtained as a yellow solid (74% yield). ^1^HNMR (DMSO‐*d*_*6*_, 400 MHz) *δ*_H_: 3.30 (2H, s, N*CH*_*2*_), 7.41 (2H, t, *J* = 8.7 Hz, Ar‐H), 7.85–7.87 (3H, m, Ar‐H), 8.02–8.04 (2H, m, Ar‐H), 8.11–8.13 (1H, m, Ar‐H), 15.07 (1H, bs, COO*H*).

2‐[3‐(4‐Fluorobenzoyl)‐4‐hydroxy‐1,1‐dioxido‐2*H*‐1,2‐benzothiazin‐2‐yl]‐*N*‐methylacetamide (**53**): Under a N_2_ atmosphere, to a solution of **52** (0.40 g, 0.0011 mmol) in dry THF (20 mL), Et_3_N (0.3 mL, 0.0021 mmol) and TBTU (0.81 g, 0.0025 mmol) were added. After 5 min of stirring, methylamine (2 M solution in THF) (1.2 mL, 0.0023 mmol) was added dropwise, and the reaction was stirred at room temperature for 2 h. The mixture was poured into ice and water, modifying the pH to 5 with 2 N HCl. The formed precipitate was filtered to obtain compound **53** as a yellow solid (77% yield). ^1^HNMR (DMSO‐*d*_*6*_, 400 MHz) *δ*_H_: 2.23 (3H, bs, N*CH*_*3*_), 3.70 (2H, bs, N*CH*_*2*_), 7.44 (2H, t, *J* = 8.3 Hz, Ar‐H), 7.67–7.69 (1H, m, Ar‐H), 7.83–7.87 (3H, m, Ar‐H), 8.06–8.10 (2H, m, Ar‐H), 15.00 (1H, bs, OH).

3‐(4‐Fluorophenyl)‐1,4‐dimethyl‐1,4‐dihydropyrazolo[4,3‐*c*][1,2]benzothiazin‐7‐ol 5,5‐dioxide (**54**): Under a N_2_ atmosphere, to a solution of compound **22** (0.10 g, 0.27 mmol) in dry DCM (4 mL), BBr_3_ (solution 1 M in DCM) (0.90 mL, 0.90 mmol) was added dropwise at 0°C and the reaction mixture was stirred at reflux for 1 h. Methanol (3 mL) was added to quench BBr_3_ and then the mixture was poured into ice/water, modifying the pH to 5 with HCl 2 N. Then, the extraction was performed with DCM (x3), the organic layer was washed with brine, and then dried with Na_2_SO_4_. After filtration, the solvent was concentrated under reduced pressure to obtain compound **54** as a yellow solid (89% yield). ^1^HNMR (DMSO‐*d*_*6*_, 200 MHz) *δ*_H_: 2.83 (3H, s, SO_2_NCH_3_), 4.01 (3H, s, NCH_3_), 7.26–7.43 (4H, m, Ar‐H), 7.92–7.95 (3H, m. Ar‐H), 10.99 (1H, s, OH).

7‐[(2,2‐Dimethyl‐1,3‐dioxolan‐4‐yl)methoxy]‐3‐(4‐fluorophenyl)‐1,4‐dimethyl‐1,4‐dihydropyrazolo[4,3‐*c*][1,2]benzothiazine 5,5‐dioxide (**55**): Under a N_2_ atmosphere, to a solution of **54** (0.10 g, 0.28 mmol) in dry DMF (2 mL), Cs_2_CO_3_ (0.27 g, 0.84 mmol) and (2,2‐dimethyl‐1,3‐dioxolan‐4‐yl)methyl 4‐methylbenzenesulfonate (0.12 g, 0.42 mmol) were added. The reaction was stirred at 80°C for 3 h. The mixture was poured into ice water and extracted with EtOAc (x3), the organic layer was washed with brine, and dried with Na_2_SO_4_. The solvent was concentrated under reduced pressure to obtain a yellow oil. After purification with flash chromatography eluting with CHCl_3_/Acetone 98/2, the target compound **55** was obtained as a yellow solid (60% yield). ^1^HNMR (Acetone‐ *d*_*6*_, 400 MHz) *δ*_H_: 1.35 (3H, s, CH_3_), 1.40 (3H, s, CH_3_), 2.91 (3H, s, SO_2_NCH_3_), 3.94–3.96 (1H, m, CH_2_ x ^1^/_2_), 4.19–4.22 (1H, m, CH_2_ x ^1^/_2_), 4.28–4.30 (5H, m, NCH_3_ and OCH_2_), 4.52–4.58 (1H, m, CH), 7.29 (2H, t, *J* = 8.8 Hz, Ar‐H), 7.45–7.51 (1H, m, Ar‐H), 7.56 (1H, d, *J* = 2.5 Hz, Ar‐H), 8.03–8.11 (3H, m, Ar‐H).

3‐Methyl‐1‐(4‐nitrophenyl)‐1,5‐dihydropyrazolo[4,3‐*c*][2,1]benzothiazine 4,4‐dioxide (**5**): To a solution of compound **28** (0.50 g, 2.09 mmol) in EtOH (15 mL), *p*‐nitro phenyl hydrazine hydrochloride (0.79 g, 4.18 mmol) was added and the reaction was stirred at reflux for 24 h. The mixture was poured into ice water, modifying the pH to 3 with 2 N HCl to obtain a precipitate that was filtered. After purification by flash chromatography column eluting with CHCl_3_/MeOH 99/1, compound **5** was obtained as a white solid (54% yield, mp 247.4°C–250.6°C). ^1^HNMR (DMSO‐*d*_*6*_, 200 MHz) *δ*_H_: 2.53 (3H, s, CH_3_), 7.01–7.04 (2H, m, Ar‐H), 7.30 (1H, d, *J* = 8.0 Hz, Ar‐H), 7.43–7.51 (1H, m, Ar‐H), 7.89 (2H, d, *J* = 8.8 Hz, Ar‐H), 8.46 (2H, d, *J* = 9.1 Hz, Ar‐H), 11.5 (1H, bs, NH). ^13^CNMR (DMSO‐*d*_*6*_, 101 MHz), *δ*_C_: 12.50 (*C*H_3_), 112.88 (C3a), 117.38 (C8), 119.85(C9), 123.37 (C9a), 124.90 (C6), 125.69 (C2′ and C6′), 128.22 (C3′ and C5′), 131.53 (C7), 137.81 (C9b), 139.07 (C4a), 143.86 (C1′), 144.59 (*C*NO_2_), 148.09 (*C*CH_3_) ppm. HPLC: Method A; tR = 6.98 min. HRMS (ESI) *m/z* calcd. for C_16_H_12_FN_4_O_4_S [M + H]^+^ 357.0652; found 357.0658.

3,5‐Dimethyl‐1‐(4‐nitrophenyl)‐1,5‐dihydropyrazolo[4,3‐*c*][2,1]benzothiazine 4,4‐dioxide (**7**): Under a N_2_ atmosphere, to a solution of **5** (0.10 g, 0.28 mmol) in dry DMF (3 mL), K_2_CO_3_ (0.07 g, 0.56 mmol) was added and after 30 min of stirring, MeI (0.04 mL, 0.56 mmol) was added; then, the mixture was stirred at room temperature for 4 h. The reaction was poured into ice and water and the formed precipitate was filtered to obtain a yellow solid. After purification by flash chromatography column eluting with CHCl_3_/MeOH 97/3, compound **7** was obtained as a yellow solid (100% yield, mp 216.9°C–219.2°C). ^1^HNMR (CDCl_3_, 200 MHz) *δ*_H_: 2.65 (3H, s, CH_3_), 3.53 (3H, s, NCH_3_), 7.02–7.13 (2H, m, Ar‐H), 7.35 (1H, d, *J* = 8.1 Hz, Ar‐H), 7.47–7.55 (1H, m, Ar‐H), 7.68 (2H, d, *J* = 9.0 Hz, Ar‐H), 8.40 (2H, d, *J* = 9.0 Hz, Ar‐H). ^13^CNMR (DMSO‐*d*_*6*_, 101 MHz), *δ*_C_: 12.45 (*C*H_3_), 31.96 (N*C*H_3_), 115.00 (C3a), 116.93 (C6), 120.69 (C8), 124.40 (C9a), 125.49 (C9), 125.69 (C2′ and C6′), 127.93 (C3′ and C5′), 131.85 (C9b), 138.88 (C7), 139.71 (C1′), 144.71 (C5a), 144.31 (*C*NO_2_), 148.01 (*C*CH_3_) ppm. HPLC: Method A; tR = 7.87 min. HRMS (ESI) *m/z* calcd. for C_17_H_14_N_4_O_4_S [M + H]^+^ 371.0809 found 371.0814.

*N*‐[2‐(3‐Methyl‐1‐phenyl‐1*H*‐pyrazol‐5‐yl)phenyl]methanesulfonamide (**8**): Under aN_2_ atmosphere, to a solution of **31** (0.20 g, 0.80 mmol) in a mixture of Pyr/CH_2_Cl_2_ (1:1) (8 mL), methane sulfonyl chloride (0.07 mL, 1.00 mmol) was added, and the mixture was stirred at room temperature for 1 h. The mixture was poured into ice and water and extracted with CH_2_Cl_2_. The organic phase was washed with brine, dried with Na_2_SO_4_, and evaporated to dryness, yielding a yellow solid. After crystallization by cyclohexane, compound **8** was obtained as a white solid (17% yield, mp 184.3°C–186.8°C). ^1^HNMR (CDCl_3_, 400 MHz) *δ*_H_: 2.46 (3H, s, CH_3_), 2.62 (3H, s, CH_3_), 6.27–6.29 (2H, m, Ar‐H and NH), 7.15 (1H, dt, *J* = 1.0 and 6.6 Hz, Ar‐H), 7.21–7.31 (6H, m, Ar‐H), 7.38–7.43 (1H, m, Ar‐H), 7.60 (1H, d, *J* = 8.1 Hz, Ar‐H). ^13^CNMR (CDCl_3,_ 101 MHz) *δ*_C_ 13.61 (*C*H_3_), 39.13 (SO_2_ *C*H_3_), 109.71 (Ar‐C), 118.59 (Ar‐C), 121.29 (Ar‐C), 123.55 (C2′ and C6′), 124.44 (Ar‐C), 127.31 (Ar‐C), 129.25 (C3′ and C5′), 130.68 (Ar‐C), 131.66 (Ar‐C), 135.12 (*C*NHSO_2_), 138.20 (Ar‐C), 139.48 (C5), 150.45 (*C*CH_3_) ppm. HPLC Method A; tR = 6.60 min. HRMS (ESI) *m/z* calcd. for C_17_H_17_N_3_O_2_S [M + H]^+^ 328.1114 found 328.1120.

3‐(4‐Chlorophenyl)‐1,4‐dihydropyrazolo[4,3‐*c*][1,2]benzothiazine 5,5‐dioxide (**9**): General procedure (D): starting from **39** and using hydrazine monohydrate (time = 2 h), after purification by flash chromatography column eluting with Cy/EtOAc 7/3, compound **4** was obtained as a yellow solid (0.15 g, 61% yield, mp 156.3°C–159.1°C). ^1^HNMR (DMSO‐*d*_*6*_, 323 K, 400 MHz) *δ*_H_: 7.84–8.15 (8H, m, Ar‐H), 10.28 (1H, bs, NH), 13.94 (^1^/_2_H, s, NH x ^1^/_2_), 14.18 (^1^/_2_H, s NH x ^1^/_2_). ^13^CNMR (DMSO‐*d*_*6*_, 323 K, 101 MHz) *δ*_C_: 123.13 (Ar‐C), 123.99 (Ar‐C), 127.85 (Ar‐C), 128.11 (Ar‐C), 128.44 (Ar‐C), 129.25 (Ar‐C), 129.33 (Ar‐C), 129.66 (Ar‐C), 129.67 (Ar‐C), 133.36 (Ar‐C), 133.67 (Ar‐C), 134.45 (*C*Cl), 141.91 (C3) ppm. HPLC Method B; tR = 6.80 min. HRMS (ESI) *m/z* calcd. for C_15_H_10_ClN_3_O_2_S [M + H]^+^ 332.0255 found 332.0264.

3‐(4‐Chlorophenyl)‐4‐methyl‐1,4‐dihydropyrazolo[4,3‐*c*][1,2]benzothiazine 5,5‐dioxide (**10**): General procedure (D): starting from **44** and using hydrazine monohydrate (time = 2 h), after purification by flash chromatography column eluting with CHCl_3_/MeOH 98/2, compound **10** was obtained as a yellow solid (0.25 g, 72% yield, mp 259.5°C–261.0°C). ^1^HNMR (DMSO‐*d*_*6*_, 323 K, 400 MHz) δ_H_: 2.89 (3H, s, NCH_3_), 7.66 (2H, d, *J* = 8.5 Hz, Ar‐H), 7.72 (1H, t, *J* = 9.6 Hz, Ar‐H), 7.89 (1H, t, *J* = 7.6 Hz, Ar‐H), 7.94‐7.99 (3H, m, Ar‐H), 8.06 (1H, d, *J* = 7.8 Hz, Ar‐H), 14.07 (1H, bs, NH). ^13^CNMR (DMSO‐*d*_*6*_, 101 MHz) δ_C_: 39.84 (*C*H_3_), 122.06 (Ar‐C), 124.10 (Ar‐C), 125.18 (Ar‐C), 126.91 (Ar‐C), 127.63 (C2′ and C6′), 128.64 (Ar‐C), 129.78 (Ar‐C), 129.89 (C3′ and C5′), 130.59 (Ar‐C), 133.59 (Ar‐C), 134.02 (Ar‐C), 136.86 (Ar‐C), 137.68 (C3) ppm. HPLC Method B; tR = 7.95 min. HRMS (ESI) *m/z* calcd. for C_16_H_12_ClN_3_O_2_S [M + H]^+^ 346.0412 found 346.0414.

4‐Methyl‐3‐phenyl‐1,4‐dihydropyrazolo[4,3‐*c*][1,2]benzothiazine 5,5‐dioxide (**11**): General procedure (D): starting from **45** and using hydrazine monohydrate (time = 3 h), after purification by flash chromatography column eluting with CHCl_3_/MeOH 99/1, compound **11** was obtained as a yellow solid (0.12 g, 47% yield, mp 224.0°C–225.5°C). ^1^HNMR (DMSO‐*d*_*6*_, 323 K, 400 MHz) δ_H_: 2.91 (3H, s, NCH_3_), 7.45–7.48 (1H, m, Ar‐H), 7.56–7.60 (2H, m, Ar‐H), 7.71 (1H, dt, *J* = 1.2 and 7.7 Hz, Ar‐H), 7.87 (1H, dt, *J* = 1.2 and 7.6 Hz, Ar‐H), 7.93–7.95 (3H, m, Ar‐H), 8.06 (1H, d, *J* = 7.2 Hz, Ar‐H), 14.15 (1H, bs, NH – observable only with the spectrum recorded at 298 K). ^13^CNMR (DMSO‐*d*_*6*_, 323 K, 101 MHz) δ_C_: 34.94 (*C*H_3_), 121.99 (Ar‐C), 124.11 (Ar‐C), 125.06 (Ar‐C), 126.28 (Ar‐C), 129.26 (Ar‐C), 129.66 (Ar‐C), 129.85 (Ar‐C), 131.08 (Ar‐C), 133.63 (Ar‐C), 133.90 (Ar‐C) ppm. HPLC Method B; tR 7.16 min. HRMS (ESI) *m/z* calcd. for C_16_H_13_N_3_O_2_S [M + H]^+^312.0801 found 312.0806.

3‐(4‐Fluorophenyl)‐4‐methyl‐1,4‐dihydropyrazolo[4,3‐*c*][1,2]benzothiazine 5,5‐dioxide (**12**): In a one‐neck round‐bottom flask, a mixture of compound **46** (0.4 g, 1.10 mmol) in hydrazine monohydrate (0.3 mL, 5.50 mmol) was irradiated with ultrasound for 20 min at 65°C. The reaction was concentrated under reduced pressure and then poured into ice water, modifying the pH to 2 with 2 N HCl. The formed precipitate was filtered to obtain an orange residue. After purification by flash chromatography eluting with CHCl_3_/MeOH 99/1, compound **12** was obtained as a yellow solid (55% yield, mp 244.9°C–247.6°C). ^1^HNMR (DMSO‐*d*_*6*_, 200 MHz) δ_H_: 2.80 (3H, s, NCH_3_), 7.32–7.40 (2H, m, Ar‐H), 7.60–7.67 (1H, m, Ar‐H), 7.76–7.99 (5H, m, Ar‐H), 13.00 (1H, bs NH). ^13^CNMR (DMSO‐*d*_*6*_, 101 MHz) δ_C_: 28.54 (*C*H_3_), 116.70 (d, *J* = 21.8 Hz, C3′ and C5′), 121.88 (Ar‐C), 124.12 (Ar‐C), 125.12 (Ar‐C), 128.37 (d, *J* = 8.1 Hz, C2′ and C6′), 129.93 (Ar‐C), 130.97 (Ar‐C), 133.93 (Ar‐C), 162.66 (*C*F, d, *J* = 247.5 Hz) ppm. HPLC Method A; tR = 7.28 min HRMS (ESI) *m/z* calcd. for C_16_H_12_FN_3_O_2_S [M + H]^+^ 330.0707 found 330.0714.

3‐(3‐Chloro‐4‐fluorophenyl)‐4‐methyl‐1,4‐dihydropyrazolo[4,3‐*c*][1,2]benzothiazine 5,5‐dioxide (**13**): General procedure (D): starting from **47** and using hydrazine monohydrate (time = 3 h), after purification by chromatography column eluting with CHCl_3_/MeOH 99/1, compound **13** was obtained as a yellow solid (0.08 g, 11% yield, mp 259.1°C–261.3°C). ^1^HNMR (DMSO‐*d*_*6*_, 323 K, 400 MHz) δ_H_: 2.88 (3H, s, NCH_3_), 7.54–7.72 (2H, m, Ar‐H), 7.80–7.83 (1H, m, Ar‐H), 7.89‐8.04 (H, m, Ar‐H), 13.97 (^1^/_2_H, s, ^1^/_2_ NH), 14.27 (^1^/_2_H, s, ^1^/_2_ NH). ^13^CNMR (DMSO‐*d*_*6*_, 323 K, 150 MHz) δ_C_: 118.40 (d, *J* = 21.6 Hz, C5′), 120.96 (d, *J* = 18.2 Hz, *C*Cl), 122.27 (Ar‐C), 124.18 (Ar‐C), 125.23 (Ar‐C), 126.94 (d, *J* = 7.5 Hz, C6′), 127.96 (Ar‐C), 130.13 (Ar‐C), 134.02 (Ar‐C), 157.70 (d, *J* = 247.5 Hz, *C*F) ppm. HPLC Method A; tR = 6.57 min. HRMS (ESI) *m/z* calcd. for C_16_H_11_ClFN_3_O_2_S [M + H]^+^ 364.0318 found 364.0323.

4‐Ethyl‐3‐(4‐fluorophenyl)‐1,4‐dihydropyrazolo[4,3‐*c*][1,2]benzothiazine 5,5‐dioxide (**14**): General procedure (D): starting from **48** and using hydrazine monohydrate (time = 50 min), after purification by flash chromatography column eluting with CHCl_3_/Acetone 98/2, compound **14** was obtained as a yellow solid (0.28 g, 40% yield, mp 283.6°C–285.3°C). ^1^HNMR (DMSO‐*d*_*6*_, 323 K, 400 MHz) δ_H_: 0.59–0.72 (3H, m, NCH_2_ *CH*_*3*_), 3.43–3.51 (2H, m, N*CH*_*2*_CH_3_), 7.34–7.52 (2H, m, Ar‐H), 7.70–7.77 (1H, m, Ar‐H), 7.84‐8.12 (5H, m, Ar‐H), 14.00 (^1^/_2_H, s, ^1^/_2_ NH), 14.32 (^1^/_2_H, s ^1^/_2_ NH). ^13^CNMR (DMSO‐*d*_*6*_, 323 K, 101 MHz) δ_C_: 11.74 (NCH_2_ *C*H_3_, major tautomer) 47.95 (N*C*H_2_CH_3_, major tautomer), 116.81 (d, *J* = 22.2 Hz – major tautomer, C3′ and C5′), 123.84 (Ar‐C), 124.19 (2 Ar‐C), 128.53 (Ar‐C), 128.74 (d, *J* = 9.1 Hz, C2′ and C6′,), 129.63 (Ar‐C), 130.20 (Ar‐C), 133.67 (Ar‐C), 134.25 (Ar‐C), 134.69 (Ar‐C) ppm. HPLC Method A; tR = 7.58 min. HRMS (ESI) *m/z* calcd. for C_17_H_14_FN_3_O_2_S [M + H]^+^ 344.0864 found 344.0867.

3‐[3‐(4‐Fluorophenyl)‐5,5‐dioxidopyrazolo[4,3‐*c*][1,2]benzothiazin‐4(1*H*)‐yl]propan‐1‐ol (**15**): General procedure (D): starting from **50** and using hydrazine monohydrate (time = 2 h), after purification by flash chromatography column eluting with CHCl_3_/MeOH 93/7, compound **15** was obtained as a yellow solid (0.32 g, 30% yield, mp 207.5°C–210.7°C). ^1^HNMR (Acetone‐*d*_*6*_, 400 MHz) δ_H_: 1.26‐1.33 (2H, m, NCH_2_ *CH*_*2*_CH_2_OH), 3.19 (2H, q, *J* = 5.8 Hz, NCH_2_CH_2_ *CH*_*2*_OH), 3.37 (1H, t. *J* = 5.2 Hz, NCH_2_CH_2_CH_2_ *OH*), 3.53–3.56 (2H, m, N*CH*_*2*_CH_2_CH_2_OH), 7.32–7.34 (2H, m, Ar‐H), 7.66–7.70 (1H, m, Ar‐H), 7.81‐7.83 (1H, m, Ar‐H), 7.86‐7.99 (3H, m, Ar‐H), 8.07 (1H, d, *J* = 7.7 Hz, Ar‐H), 12.96 (1H, bs, NH). ^13^CNMR (Acetone, 150 MHz) δ_C_: 50.02 (N*C*H_2_), 58.48 (NCH_2_ *C*H_2_CH_2_OH), 58.62 (OH*C*H_2_), 116.00 (Ar‐C), 119.76 (Ar‐C), 126.69 (Ar‐C), 123.73 (d, *J* = 4.5 Hz, C1′), 123.75 (Ar‐C), 128.58 (d, *J* = 9.0 Hz, C2′ and C6′), 129.14 (Ar‐C), 132.85 (Ar‐C), 162.90 (d, *J* = 247.8 Hz, *C*F) ppm. HRMS (ESI) *m/z* calcd. for C_18_H_16_FN_3_O_3_S [M + H]^+^ 374.0969 found 374.0977.

2‐[3‐(4‐Fluorophenyl)‐5,5‐dioxidopyrazolo[4,3‐*c*][1,2]benzothiazin‐4(1*H*)‐yl]‐*N*‐methylacetamide (**16**): General procedure (D): starting from **53** and using hydrazine monohydrate (time = 5 h), after purification by flash chromatography column eluting with CHCl_3_/MeOH 97/3, compound **16** was obtained as a yellow solid (0.20 g, 23% yield, mp 223.8°C–225.5°C). ^1^HNMR (DMSO‐*d*_*6*_, 323 K, 600 MHz) *δ*_H_: 2.24 (3H, s, N*CH*_*3*_), 4.03–4.09 (2H, m, N*CH*_*2*_), 7.37–7.38 (1H, m, Ar‐H), 7.42–745 (1H, m, Ar‐H), 7.60–7.64 (1H, m, Ar‐H), 7.66–7.67 (1H, m, CONH), 7.78–7.99 (4H, m, Ar‐H), 8.00–8.11 (1H, m, Ar‐H), 13.10 (^1^/_2_H, s, ^1^/_2_ x NH), 14.04 (^1^/_2_H, s, ^1^/_2_ x NH). ^13^CNMR (DMSO‐*d*_*6*_, 323 K, 150 MHz) *δ*_C_: 25.68 (*C*H_3_), 53.42 (N*C*H_2_CO, major tautomer), 116.68 (d, *J* = 21.0 Hz – major tautomer, C3′ and C5′), 119.87 (Ar‐C, major tautomer), 123.06 (Ar‐C, major tautomer), 123.76 (Ar‐C, major tautomer), 124.45 (Ar‐C, major tautomer), 128.61 (Ar‐C, major tautomer), 128.99 (Ar‐C, major tautomer), 129.06 (Ar‐C), 131.64 (Ar‐C), 132.79 (d, *J* = 12.0 Hz ‐ major tautomer, C2′ and C6′), 134.64 (Ar‐C), 140.11 (Ar‐C), 141.87 (Ar‐C), 162.77 (d, *J* = 244.5 Hz, *C*F), 166.66 (*C* = O) ppm. HRMS (ESI) *m/z* [M + H]^+^ calcd. for C_18_H_15_FN_4_O_3_S [M + H]^+^ 387.0922 found 387.0927.

2‐[3‐(4‐Fluorophenyl)‐5,5‐dioxidopyrazolo[4,3‐*c*][1,2]benzothiazin‐4(1*H*)‐yl]‐*N*‐methylethanamine (**17**): Under a N_2_ atmosphere, in a 3‐neck round‐bottom flask, to a solution of compound **16** (0.2 g, 0.52 mmol) in dry THF (5 mL), LiAlH_4_ (0.16 g, 4.22 mmol) was added, and the reaction was stirred at reflux for 48 h. The mixture was quenched with EtOAc and MeOH then it was poured into ice and water to obtain a precipitate that was filtered. After purification by flash chromatography column eluting with CHCl_3_/MeOH 97/3, compound **17** was obtained as a white solid (18% yield, mp 188.7°C–190.3°C). ^1^HNMR (DMSO‐*d*_*6*_, 323 K, 400 MHz) *δ*_H_: 1.77 (3H, s, N*CH*_*3*_), 2.06 (2H, t, *J* = 6.9 Hz, *CH*_*2*_N), 3.36 (2H, t, *J* = 6.9 Hz, N*CH*_*2*_), 7.39 (2H, t, *J* = 8.8 Hz, Ar‐H), 7.65 (1H, dt, *J* = 0.9 and 7.8 Hz, Ar‐H), 7.80 (1H, dt, *J* = 1.1 and 7.7 Hz, Ar‐H), 7.86–7.89 (3H, m, Ar‐H), 7.99 (1H, d, *J* = 7.2 Hz, Ar‐H). ^13^CNMR (DMSO‐*d*_*6*_, 323 K, 101 MHz) *δ*_C_: 35.32 (N*C*H_3_), 47.28 (N*C*H_2_), 51.38 (CH_3_NH*C*H_2_), 116.63 (d, *J* = 22.2 Hz, C3′ and C5′), 119.22 (Ar‐C), 123.80 (Ar‐C), 124.05 (Ar‐C), 125.73 (Ar‐C), 126.42 (Ar‐C), 128.75 (d, *J* = 9.1 Hz, C2′ and C6′), 129.89 (Ar‐C), 133.01 (Ar‐C), 133.64 (Ar‐C), 162.64 (*C*F, d, *J* = 248.5 Hz) ppm. HRMS (ESI) *m/z* calcd. for C_18_H_17_FN_4_O_2_S [M + H]^+^ 373.1129 found 373.1134.

3‐(4‐Chlorophenyl)‐4‐methyl‐1‐(2‐piperidin‐1‐ylethyl)‐1,4‐dihydropyrazolo[4,3‐*c*][1,2]benzothiazine 5,5‐dioxide (**18**): In a 3‐neck round‐bottom flask, to a suspension of 60% NaH (0.07 g, 1.85 mmol) in dry THF (10 mL), compound **10** (0.16 g, 0.46 mmol) was added dropwise and after 15 min of stirring, 2‐chloro ethylpiperidine (0.26 g, 1.39 mmol) was added. The reaction was stirred at room temperature for 2 h. The mixture was poured into ice and water and extracted with EtOAc. The organic phase was washed with brine and dried with Na_2_SO_4_. The solvent was evaporated to dryness under vacuum to obtain an orange solid. After purification by flash chromatography column eluting with CHCl_3_/acetone 98/2, compound **18** was obtained as a yellow solid (21% yield, mp 150.2°C–153.0°C). ^1^HNMR (CDCl_3_, 400 MHz,), *δ*_H_: 1.37–1.39 (2H, m, piperidine CH_2_), 1.53–1.55 (4H, m, piperidine CH_2_ x2), 2.46–2.48 (4H, m, piperidine NCH_2_ x2), 2.84 (3H, s, NCH_3_), 3.01–3.03 (2H, m, NCH_2_ *CH*_*2*_N), 4.60–4.62 (2H, m, N*CH*_*2*_CH_2_N), 7.38 (2H, d, *J* = 8.6 Hz, Ar‐H), 7.55–7.59 (1H, m, Ar‐H), 7.70–7.73 (1H, m, Ar‐H), 7.91 (2H, d, *J* = 8.5 Hz, Ar‐H), 7.99 (2H, dd, *J* = 1.0 and 6.9 Hz, Ar‐H). ^13^CNMR (CDCl_3_, 101 MHz), *δ*_C_: 23.66 (piperidine *C*H_2_), 25.34 (piperidine *C*H_2_ x2), 29.71 (N*C*H_3_), 38.73 (NC*H*_2_), 54.94 (piperidine N*C*H_2_ x2), 57.77 (pyrazole N*C*H_2_), 122.92 (Ar‐C), 124.44 (Ar‐C), 124.55 (Ar‐C), 126.12 (Ar‐C), 129.07 (Ar‐C), 129.21 (Ar‐C), 129.74 (Ar‐C), 130.95 (Ar‐C), 131.05 (Ar‐C), 133.04 (Ar‐C), 134.46 (Ar‐C), 142.56 (C3) ppm. HPLC Method A; tR = 5.22 min. HRMS (ESI) *m/z* calcd. for C_23_H_25_ClN_4_O_2_S [M + H]^+^ 457.1460 found 457.1467.

3‐(4‐Chlorophenyl)‐4‐methyl‐2‐(2‐piperidin‐1‐ylethyl)‐2,4‐dihydropyrazolo[4,3‐c][1,2]benzothiazine 5,5‐dioxide (**18a**): Yellow solid, 5% yield. ^1^HNMR (CDCl_3_, 400 MHz,), *δ*_H_: 1.31–1.37 (2H, m, piperidine CH_2_), 1.43–1.47 (4H, m, piperidine CH_2_ x2), 2.26–2.31 (4H, m, piperidine CH_2_ x2), 2.74 (3H, s, NCH_3_), 2.75 (2H, t, *J* = 7.0 Hz, NCH_2_ *CH*_*2*_N), 4.15 (2H, t, *J* = 7.0 Hz, N*CH*_*2*_CH_2_N), 7.45 (2H, d, *J* = 8.4 Hz, Ar‐H), 7.48 (1H, dt, *J* = 1.2 and 7.7 Hz, Ar‐H), 7.56 (2H, d, *J* = 8.4 Hz, Ar‐H), 7.62 (1H, dt, *J* = 1.2 and 7.7 Hz, Ar‐H), 7.87 (1H, dd, *J* = 1.0 and 7.9 Hz, Ar‐H), 8.00 (1H, dd, *J* = 1.0 and 7.9 Hz, Ar‐H).

3‐(4‐Fluorophenyl)‐1,4‐dimethyl‐7‐(2‐morpholin‐4‐ylethoxy)‐1,4‐dihydropyrazolo[4,3‐*c*][1,2]benzothiazine 5,5‐dioxide (**19**): Under a N_2_ atmosphere, to a solution of **54** (0.10 g, 0.28 mmol) in dry DMF (2 mL), Cs_2_CO_3_ (0.32 g, 0.98 mmol) and 4‐(2‐chloroethyl) morpholine (0.05 g, 0.33 mmol) were added. The reaction was stirred at 80°C for 1 h and 30 min. The mixture was poured into ice water and the formed precipitate was filtered to obtain a yellow solid. After purification with flash chromatography eluting with CHCl_3_/Acetone 8/2, the target compound **19** was obtained as a yellow solid (38% yield, mp 174.2°C–176.8°C). ^1^HNMR (Acetone‐*d*_*6*_, 400 MHz) *δ*_H_: 2.54–2.60 (4H, m, morpholine NCH_2_ x2), 2.83–2.84 (2H, m, OCH_2_ *CH*_*2*_N), 2.91 (3H, s, NCH_3_), 3.63 (4H, t, *J* = 4.4 Hz, morpholine OCH_2_ x2), 4.28 (3H, s, NCH_3_), 4.37 (2H, t, *J* = 5.5 Hz, O*CH*_*2*_CH_2_N), 7.28 (2H, t, *J* = 8.9 Hz, Ar‐H), 7.45 (1H, dd, *J* = 2.6 and 8.8 Hz, Ar‐H), 7.54 (1H, d, *J* = 2.6 Hz, Ar‐H), 8.04–8.11 (3H, m, Ar‐H). ^13^CNMR (Acetone‐*d*_*6*_, 101 MHz) *δ*_C_ 38.51 (SO_2_N*C*H_3_), 39.50 (N*C*H_3_), 54.01 (morpholine N*C*H_2_ x2), 57.26 (N*C*H_2_), 66.54 (O*C*H_2_), 66.79 (morpholine O*C*H_2_ x2), 110.83 (Ar‐C), 115.60 (d, *J* = 21.9 Hz, C3′ and C5′), 117.70 (Ar‐C), 119.74 (Ar‐C), 126.56 (Ar‐C), 127.94 (d, *J* = 8.3 Hz, C2′ and C6′), 128.28 (Ar‐C), 130.68 (Ar‐C), 132.46 (Ar‐C), 140.97 (C3), 159.47 (C7), 162.72 (d, *J* = 246.6 Hz, *C*F) ppm. HPLC Method A; tR = 4.25 min. HRMS (ESI) *m/z* calcd. for C_23_H_25_FN_4_O_4_S [M + H]^+^ 473.1654 found 473.1652.

3‐(4‐Fluorophenyl)‐1,4‐dimethyl‐7‐(2‐piperidin‐1‐ylethoxy)‐1,4‐dihydropyrazolo[4,3‐*c*][1,2]benzothiazine 5,5‐dioxide (**20**): Under a N_2_ atmosphere, in a 3‐neck round‐bottom flask, to a solution of **54** (0.10 g, 0.28 mmol) in dry DMF (2 mL), Cs_2_CO_3_ (0.32 g, 0.98 mmol) and 4‐(2‐chloroethyl)piperidine (0.06 g, 0.33 mmol) were added. The reaction was stirred at 80°C for 5 h. The mixture was poured into ice water and the formed precipitate was filtered to obtain a yellow solid. After purification with flash chromatography eluting with CHCl_3_/MeOH 98/2, the target compound **20** was obtained as a yellow solid (20% yield, mp 158.0°C–160.0°C). ^1^HNMR (CDCl_3_, 400 MHz) *δ*_H_: 1.40–1.41 (2H, m, piperidine CH_2_), 1.55–1.57 (4H, m, piperidine CH_2_ x2), 2.46–2.47 (4H, m, piperidine NCH_2_ x2), 2.77 (2H, t, *J* = 8.0 Hz, OCH_2_ *CH*_*2*_N), 2.85 (3H, s, NCH_3_), 4.15–4.18 (5H, m, NCH_3_ and O*CH*_*2*_CH_2_N), 7.08 (2H, t, *J* = 8.0 Hz, Ar‐H), 7.22–7.23 (1H, m, Ar‐H), 7.50 (1H, d, *J* = 4.0 Hz, Ar‐H), 7.62 (1H, d, *J* = 8.0 Hz, Ar‐H), 7.19–7.95 (2H, m, Ar‐H). ^13^CNMR (CDCl_3_, 101 MHz) *δ*_C_: 24.09 (piperidine *C*H_2_), 25.85 (piperidine *C*H_2_ x2), 38.83 (SO_2_N*C*H_3_), 39.98 (N*C*H_3_), 55.07 (piperidine N*C*H_2_ x2), 57.64 (N*C*H_2_), 66.80 (O*C*H_2_), 111.04 (Ar‐C), 115.83 (d, *J* = 21.2 Hz, C3′ and C5′), 117.29 (Ar‐C), 120.11 (Ar‐C), 121.44 (Ar‐C), 125.36 (Ar‐C), 127.57 (Ar‐C), 128.24 (d, *J* = 8.1 Hz, C2′ and C6′), 130.60 (Ar‐C), 132.40 (Ar‐C), 142.07 (C3), 159.30 (C7), 162.87 (d, *J* = 247.5 Hz, *C*F) ppm. HPLC Method A; tR = 4.5 min. HRMS (ESI) *m/z* calcd. for C_24_H_2_FN_4_O_3_S [M + H]^+^ 471.1861 found 471.1862.

3‐{[3‐(4‐Fluorophenyl)‐1,4‐dimethyl‐5,5‐dioxido‐1,4‐dihydropyrazolo[4,3‐*c*][1,2]benzothiazin‐7‐yl]oxy}propane‐1,2‐diol (**21**): Compound **55** (0.17 g, 0.36 mmol) was dissolved in a mixture of HCl 9% in H_2_O (3 mL) and MeOH (6 mL) and the reaction was warmed to reflux for 1 h. The mixture was poured into ice water, modifying the pH to 4 with sol. sat. NaHCO_3_ to obtain a precipitate that was filtered. After purification by flash chromatography column eluting with CHCl_3_/MeOH 95/5, the target compound **21** was obtained as a yellow solid (45% yield, mp 180.2°C–182.5°C). ^1^HNMR (Acetone‐ *d*_*6*_, 200 MHz) *δ*_H_: 2.71 (3H, s, SO_2_NCH_3_), 3.66 (2H, t, *J* = 5.5 Hz, OCH_2_), 3.81–3.88 (1H, m, CH), 3.99–4.31 (5H, m, NCH_3_ and OCH_2_), 7.23 (2H, t, *J* = 8.8 Hz, Ar‐H), 7.36–7.49 (2H, m, Ar‐H), 7.98–8.07 (3H, m, Ar‐H). ^13^CNMR (Acetone‐ *d*_*6*_, 150 MHz) *δ*_H_: 38.93 (SO_2_N*C*H_3_), 39.91 (N*C*H_3_), 63.40 (CH*C*H_2_OH), 70.77 (O*C*H_2_), 70.87 (*C*HOH), 111.49 (Ar‐C), 116.00 (d, *J* = 21.2 Hz, C3′ and C5′), 117.46 (Ar‐C), 119.96 (Ar‐C), 121.88 (Ar‐C), 127.00 (Ar‐C), 128.38 (d, *J* = 7.7 Hz, C2′ and C6′), 128.72 (d, *J* = 3.0 Hz, C1′), 131.12 (Ar‐C), 132.90 (Ar‐C), 141.43 (C3), 160.13 (C7), 163.01 (d, *J* = 246.9 Hz, *C*F) ppm. HPLC Method A; tR = 6.57 min. HRMS (ESI) *m/z* calcd. for C_20_H_20_FN_3_O_5_S [M + H]^+^ 434.1181 found 434.1181.

3‐(4‐Fluorophenyl)‐7‐methoxy‐1,4‐dimethyl‐1,4‐dihydropyrazolo[4,3‐*c*][1,2]benzothiazine 5,5‐dioxide (**22**): General procedure (D): starting from **48** and using methyl hydrazine monohydrate (time = 1 h), after purification by flash chromatography column eluting with Cy/EtOAc 6/4, compound **22** was obtained as a yellow solid (0.12 g, 80% yield, mp 218.0°C–220.0°C). ^1^HNMR (DMSO‐*d*_*6*_, 323 K, 400 MHz) *δ*_H_: 2.90 (3H, s, SO_2_NHCH_3_), 4.00 (3H, s, OCH_3_), 4.28 (3H, s, NCH_3_), 7.36–7.42 (2H, m, Ar‐H), 7.50–7.54 (2H, m, Ar‐H), 8.00–8.05 (2H, m, Ar‐H), 8.09 (1H, d, *J* = 0.8.6 Hz, Ar‐H). ^13^CNMR (DMSO‐*d*_*6*_, 323 K, 101 MHz) δ_C_ 39.43 (SO_2_N*C*H_3_), 40.68 (N*C*H_3_), 56.54 (O*C*H_3_), 110.45 (Ar‐C), 116.48 (d, *J* = 22.2 Hz, C3′ and C5′), 116.84 (Ar‐C), 120.14 (Ar‐C), 121.22 (Ar‐C), 127.48 (Ar‐C), 128.10 (Ar‐C), 128.19 (d, *J* = 8.0 Hz, C2′ and C6′), 130.84 (Ar‐C), 132.08 (Ar‐C), 140.82 (C3), 160.27 (C7), 162.46 (d, *J* = 246.4 Hz, *C*F) ppm. HRMS (ESI) *m/z* calcd. for C_18_H_16_FN_3_O_3_S [M + H]^+^ 374.0969 found 374.0969.

{4‐[2‐(Diethylamino)ethoxy]‐2‐methyl‐1,1‐dioxido‐2*H*‐1,2‐benzothiazin‐3‐yl}(4‐fluorophenyl)methanone (**23**): Under a N_2_ atmosphere, to a solution of **46** (0.30 g, 0.90 mmol) in dry DMF (6 mL), K_2_CO_3_ (0.48 g, 3.6 mmol) and 2‐chloro‐*N*,*N*‐diethylethan‐1‐amine hydrochloride (0.47 g, 2.7 mmol) were added and the mixture was stirred at 80°C for 2 h. The reaction mixture was poured into ice water and extracted with EtOAc (x3); the organic layer was washed with brine and dried with Na_2_SO_4_. The solvent was concentrated under reduced pressure to obtain a brown oil. After purification by flash chromatography column eluting with CHCl_3_/MeOH 98/2, compound **23** was obtained as a white solid (30% yield, mp 192.3°C–194.7°C). ^1^HNMR (DMSO‐*d*_*6*_, 323 K, 600 MHz) *δ*_H_: 0.77 (6H, t, *J* = 9.9 Hz, NCH_2_ *CH*_*3*_ x2), 2.27–2.29 (4H, m, N*CH*_*2*_CH_3_ x2), 2.35–2.36 (2H, m, OCH_2_ *CH*_*2*_N), 2.92 (3H, s, NCH_3_), 3.65–3.66 (2H, m, O*CH*_*2*_CH_2_N), 7.45 (2H, t, *J* = 8.3 Hz, Ar‐H), 7.82 (1H, t, *J* = 7.6 Hz, Ar‐H), 7.89 (1H, t, *J* = 7.5 Hz, Ar‐H), 7.95 (2H, t, *J* = 7.7 Hz, Ar‐H), 8.07–8.09 (2H, m, Ar‐H). ^13^CNMR (DMSO‐*d*_*6*_, 150 MHz) δ_C_ 11.92 (NCH_2_ *C*H_3_ x2), 29.47 (SO_2_N*C*H_3_), 47.09 (N*C*H_2_CH_3_ x2), 51.10 (N*C*H_2_), 51.90 (O*C*H_2_), 113.98 (Ar‐C), 116.48 (d, *J* = 22.2 Hz, C3′ and C5′), 122.91 (Ar‐C), 125.92 (Ar‐C), 128.00 (Ar‐C), 129.09 (Ar‐C), 130.82 (Ar‐C), 131.72 (Ar‐C), 132.77 (d, *J* = 10.0 Hz, C2′ and C6′), 133.38 (Ar‐C), 134.21 (Ar‐C), 145.86 (C‐O), 166.01 (d, *J* = 254.2 Hz, *C*F), 188.28 (*C* = O) ppm. HPLC Method B; tR 4.23 min. HRMS (ESI) *m/z* calcd. for C_22_H_25_FN_2_O_4_S [M + H]^+^ 433.1592 found 433.1595.

(4‐Fluorophenyl)[2‐methyl‐1,1‐dioxido‐4‐(2‐piperidin‐1‐ylethoxy)‐2*H*‐1,2‐benzothiazin‐3‐yl]methanone (**24**): Following the same procedure as that reported for the synthesis of compound **23** but using 1‐(2‐chloroethyl) piperidine hydrochloride (0.26 g, 1.39 mmol), after purification by flash chromatography column eluting with CHCl_3_/MeOH 98/2, compound **24** was obtained as a white solid (30% yield, mp 183.2°C–185.5°C).^1^HNMR (DMSO‐*d*_*6*_, 323 K, 600 MHz) *δ*_H_: 1.24–1.36 (6H, m, piperidine CH_2_ x3), 2.11–2.15 (4H, m, piperidine CH_2_ x2), 2.28 (2H, t, *J* = 5.3 Hz, OCH_2_ *CH*_*2*_N), 2.92 (3H, s, NCH_3_), 3.73 (2H, t, *J* = 5.4 Hz, O*CH*_*2*_CH_2_N), 7.45 (2H, t, *J* = 6.9 Hz, Ar‐H), 7.82 (1H, dt, *J* = 1.1 and 7.6 Hz, Ar‐H), 7.90 (1H, dt, *J* = 1.2 and 7.5 Hz, Ar‐H), 7.94–7.98 (2H, m, Ar‐H), 8.07–8.09 (2H, m, Ar‐H). ^13^CNMR (DMSO‐*d*_*6*_, 323 K, 150 MHz) *δ*_C_: 24.17 (piperidine *C*H_2_), 25.70 (piperidine *C*H_2_ x2), 35.56 (SO_2_N*C*H_3_), 54.35 (piperidine N*C*H_2_ x2), 57.73 (N*C*H_2_), 71.95 (O*C*H_2_), 116.47 (d, *J* = 22.2 Hz, C3′ and C5′), 122.88 (Ar‐C), 126.05 (Ar‐C), 129.06 (Ar‐C), 129.12 (Ar‐C), 131.70 (Ar‐C), 132.81 (d, *J* = 10.1 Hz, C2′ and C6′), 133.32 (Ar‐C), 133.59 (Ar‐C), 134.21 (Ar‐C), 145.82 (C‐O), 165.99 (d, *J* = 254.52 Hz, *C*F), 188.17 (C═O) ppm. HPLC Method B; tR = 4.11 min. HRMS (ESI) *m/z* calcd. for C_23_H_25_FN_2_O_4_S [M + H]^+^ 445.1592 found 445.1594.

### 4.2 Bacterial Strains

In this study, the *S. aureus* strains SA‐1199B (overexpressing *norA*) and its isogenic parent SA‐1199 (wild type) [37], the pair SA‐K1902 (*norA*‐depleted) and SA‐K2378 (complemented with a pCU1 plasmid harboring the *norA* promoting and coding region from SA‐1199B), were used to test EPI activity as described in a previous study [38]. Furthermore, the *S. aureus* ATCC 29213 was used as the reference strain in MIC determination experiments.

### 4.3 EtBr Efflux Assay

The cells were grown overnight in cation‐supplemented Mueller–Hinton broth (CAMHB) without any additives at 35°C. The organisms were subsequently diluted into CAMHB to obtain an optical density at 600 nm (OD_600_) of 0.7–0.8. Subsequently, the cells were pelleted and resuspended at an OD_600_ of 0.8 in 0.5 mL aliquots of CAMHB containing EtBr and carbonyl cyanide m‐chlorophenylhydrazone (CCCP) to “load” the cells with EtBr (final concentrations of 25 μM for EtBr and 100 μM for CCCP). Following 20 min of incubation at room temperature, the cells were pelleted and resuspended in 1 mL of fresh CAMHB. Immediately thereafter, 200 μL aliquots were transferred into the wells of white 96‐well plates, which either lacked or contained 50 or 10 μM of each test compound. Fluorescence was monitored continuously using a microplate reader (Infinite M200 Pro, TECAN) at excitation and emission wavelengths of 485 and 645 nm, respectively, for a period of 5 min. The experiments were conducted in triplicate, with two technical replicates per biological replicate. The efflux activity of the SA‐1199B strain was expressed as a percentage of fluorescence decrease over a 5‐min time course. The inhibition of this efflux by the test compounds was determined using the following equation: ([efflux in the absence] ‐ [efflux in the presence of test [compound])/[efflux in the absence of test compound] × 100, which yielded the percent efflux inhibition observed.

### 4.4 MIC Determination

The MIC determination of CPX and EPI compounds was performed using the broth microdilution method, according to CLSI guidelines [39]. CPX MIC was interpreted using CLSI breakpoints [40]. MIC of CPX was also determined for SA‐1199 and 1199B in association with fixed concentrations of EPIs ranging from 1/2 to 1/16 MIC of each compound.

### 4.5 Checkerboard Assays

Checkerboard assays were carried out using SA‐K1902 and SA‐K2378 strains according to standard procedures [41]. CPX concentrations ranged from 0.01 to 10 µg/mL and EPIs ranged from 0.2 to 12.5 µg/mL. The combinations leading to a ≥ fourfold reduction of the CPX MIC were considered synergistic.

### 4.6 Biofilm Formation Inhibition

The biofilm production by SA‐K1902 and SA‐K2378 strains was analyzed in flat 96‐well microtiter plates as previously described [34], with some modifications. The overnight *S. aureus* cultures were diluted to reach 0.1 optical density (650 nm) in TSB supplemented with 1% glucose and the bacteria were incubated in the absence or presence of CPX and EPIs alone or in association. The tested concentrations of CPX were MIC, ½ MIC, and ¼ MIC; EPI concentrations were those showing synergy with CPX in the checkerboard test. The microplates were incubated at 37°C for 24 h in static conditions. After incubation, the biofilms were washed three times with distilled water, dried at 60°C for 1 h, and stained with 0,1% crystal violet for 10 min. After staining, the crystal violet was removed and the wells were washed three times with water, dried at 37°C for 30 min, and the bound crystal violet was solubilized with 96% ethanol in shaking conditions. At the end of the destaining, the absorbance (570 nm) of the solutions in each well was measured using the FLUOstar Omega plate reader (BMG‐LABTECH, Germany) to quantify the biofilm produced in each condition compared with the control (biofilm produced by the strain without any compound/antibiotic). Biofilm assays were performed in triplicate for each condition.

### 4.7 Cytotoxicity Assays

The MTT assay was performed on two human ATCC cell lines, that is, A459 (CCL‐185) and MCF7 (HTB‐22), to test the potential cytotoxic effect of the compounds, after 72 h exposure, as previously described [42]. Each compound was tested at concentrations ranging from 12.5 to 100 μg/mL. Untreated cells were considered as controls. The percentage of viable cells was calculated as follows: % Cell viability = 100 × treated well absorbance/untreated control well absorbance. All assays were performed once in technical quadruplicate.

## Conflicts of Interest

The authors declare no conflicts of interest.

## Supporting information

Supporting_Information.

ArchPharm_SupplMat_InChI.

## Data Availability

The data that support the findings of this study are available in the Supporting Information of this article.
