# Supplementary material for: NorA Efflux Pump Inhibitors: Expanding SAR Knowledge of Pyrazolo[4,3‐c][1,2]benzothiazine 5,5‐Dioxide Derivatives
Source: Arch Pharm (Weinheim). 2025 May 19;358(5):e70000. doi: 10.1002/ardp.70000 (PMC12089509; doi:10.1002/ardp.70000)
Supplement: Supplementary file 1 — Supporting_Information. [file ARDP-358-e70000-s001.docx]

**NorA Efflux Pump Inhibitors: Expanding SAR Knowledge of Pyrazolo[4,3-*c*][1,2]benzothiazine 5,5-dioxide Derivatives**

**Authors**

Giada Cernicchi^1^, Alessandra Di Gregorio^2^, Tommaso Felicetti^1,*^, Elisa Rampacci^3^, Giulia Casari^4^, Tatiana Armeni^4^, Brenda Romaldi^4^, Ermelinda Zefaj^1^, Fabrizio Passamonti^3^, Serena Massari^1^, Giuseppe Manfroni^1^, Maria Letizia Barreca^1^, Oriana Tabarrini^1^, Carla Vignaroli^2,*^, Stefano Sabatini^1^

**Affiliations**

^1^Department of Pharmaceutical Sciences, Università degli Studi di Perugia, via del Liceo 1, 06123, Perugia, Italy

^2^Department of Life and Environmental Science, Università Politecnica delle Marche, via Brecce Bianche, 60131, Ancona, Italy

^3^Department of Veterinary Medicine, Università degli Studi di Perugia, via San Costanzo 4, 06126, Perugia, Italy

^4^Dipartimento di Scienze Cliniche Specialistiche ed Odontostomatologiche-Sez. Biochimica, Biologia e Fisica, Università Politecnica delle Marche, 60131 Ancona, Italy

*Corresponding author; E-mail addresses: tommaso.felicetti@unipg.it (T. Felicetti), c.vignaroli@univpm.it (C. Vignaroli).

^||^The authors contributed equally to this work.

**Supplementary material**

Contents Pages

**Figures S1-S5.** 2D ^1^H NMR NOESY S1-S3

**Figures S6-S99.** ^1^H NMR and ^13^C NMR spectra S4-S52

**Figures S100-S118.** HPLC chromatograms S53-S62


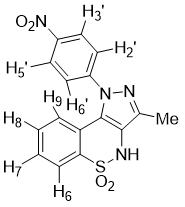


**
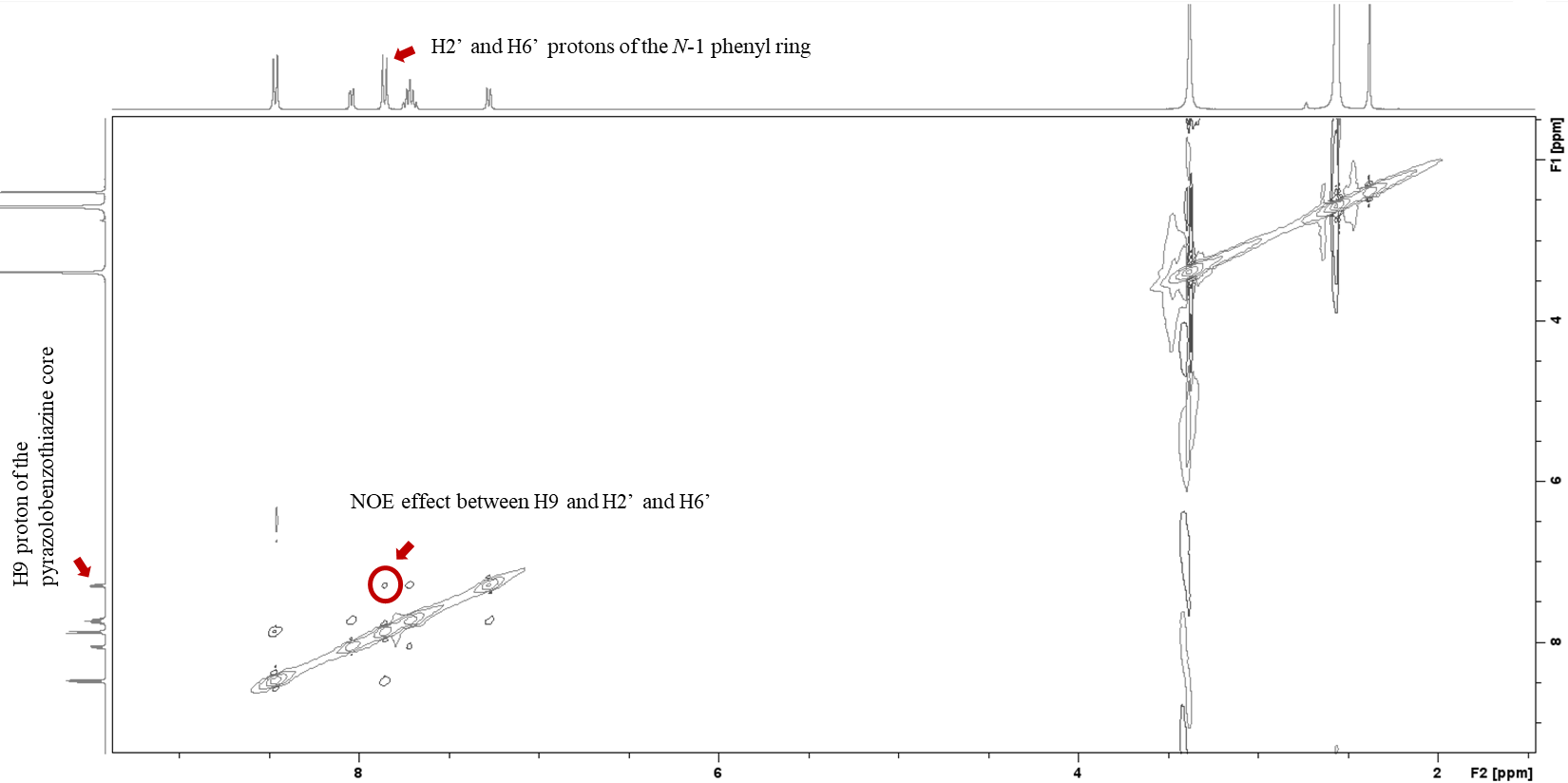
**

**Figure S1.** NOESY (400 MHz, DMSO-*_d6_*) spectrum of **4.** The red arrows indicate the protons that were involved in the interaction, while the red circle provides evidence of the NOE effect.


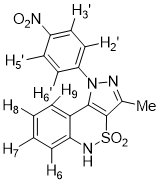


**
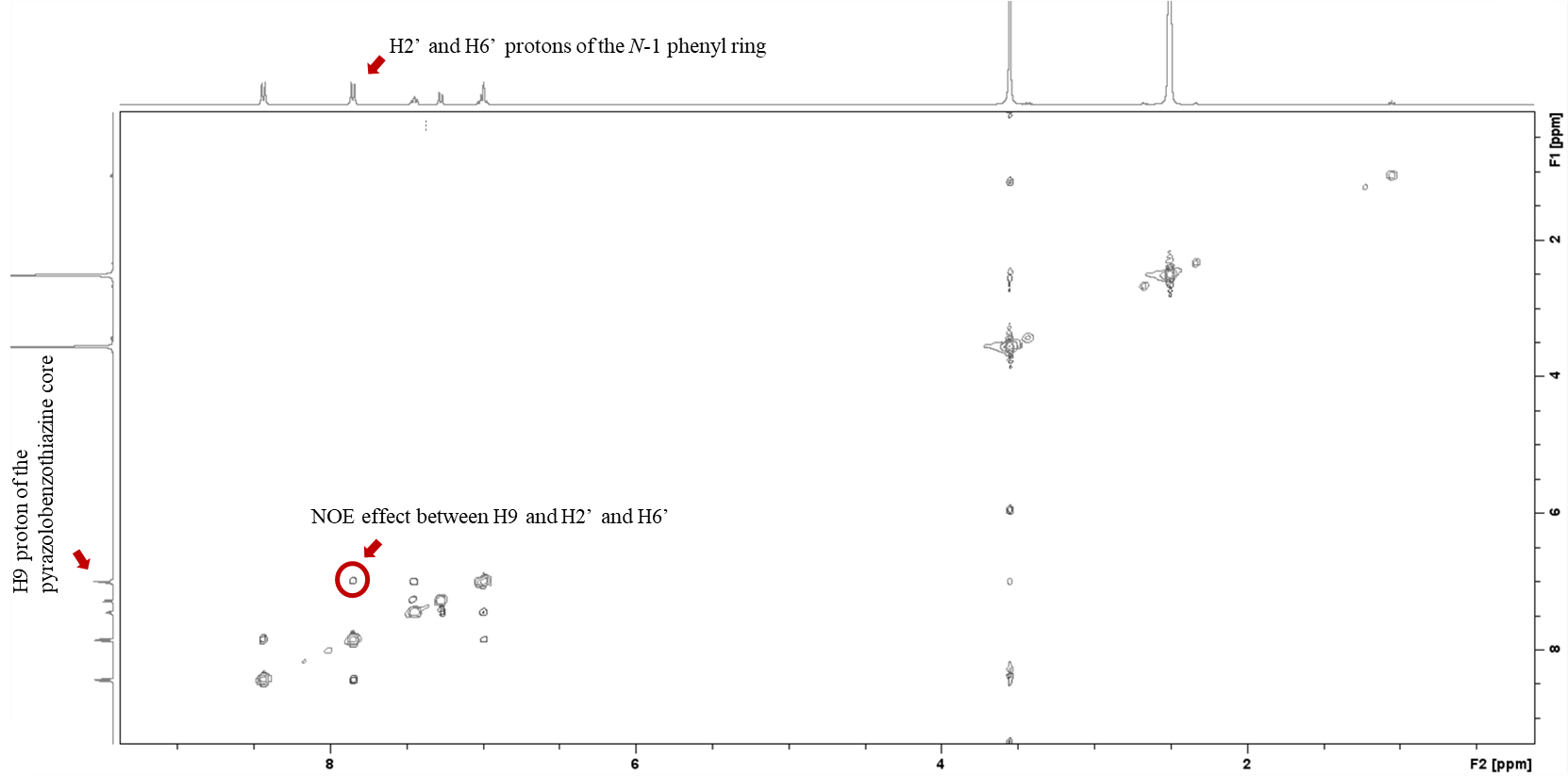
**

**Figure S2.** NOESY (400 MHz, DMSO-*_d6_*) spectrum of **5.** The red arrows indicate the protons that were involved in the interaction, while the red circle provides evidence of the NOE effect.

**
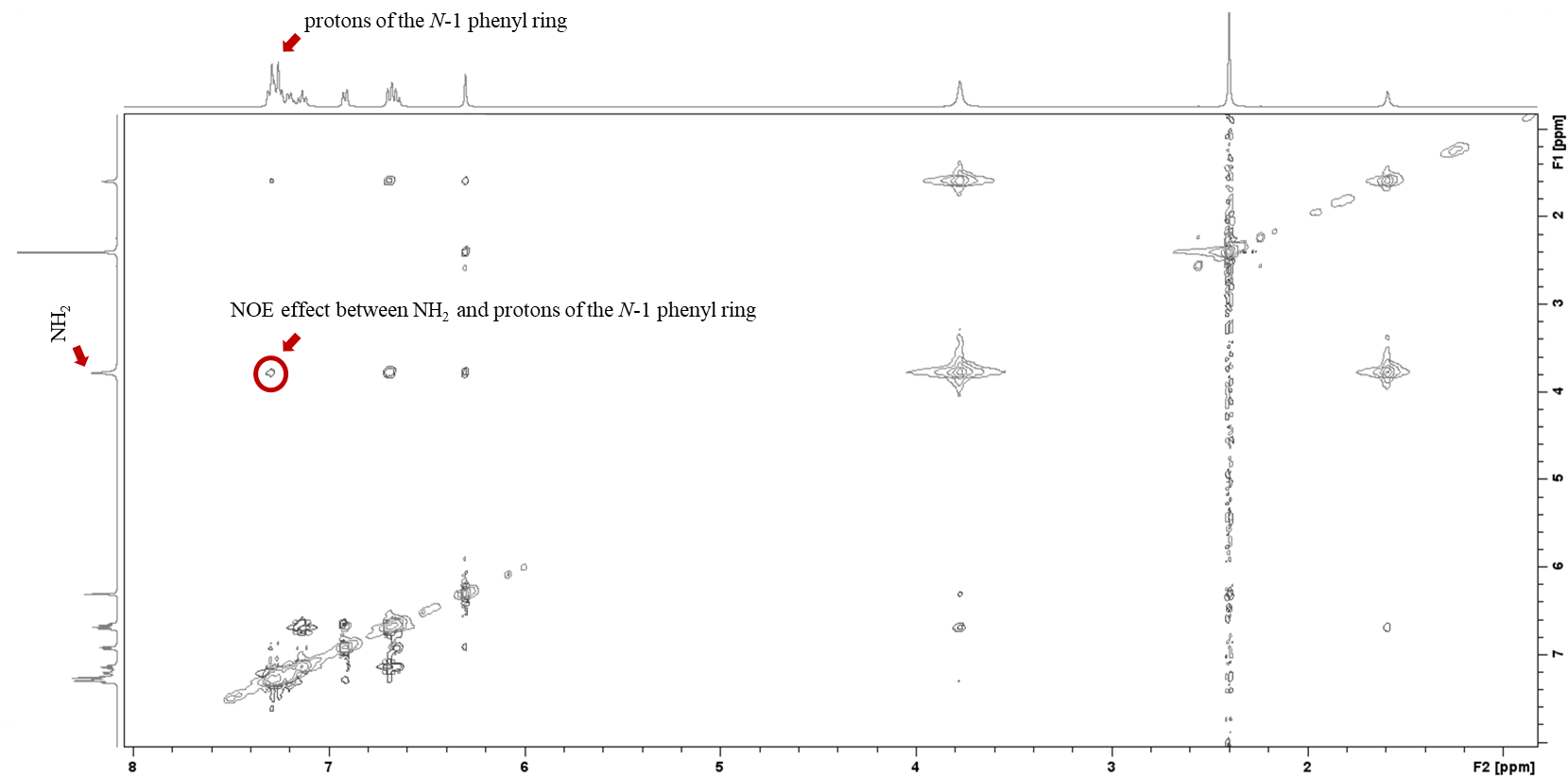
**

**Figure S3.** NOESY (400 MHz, CDCl_3_) spectrum intermediate **31.** The red arrows indicate the **
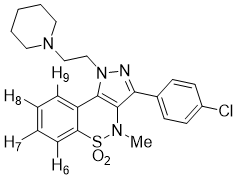
**that were involved in the interaction, while the red circle provides evidence of the NOE effect.

**
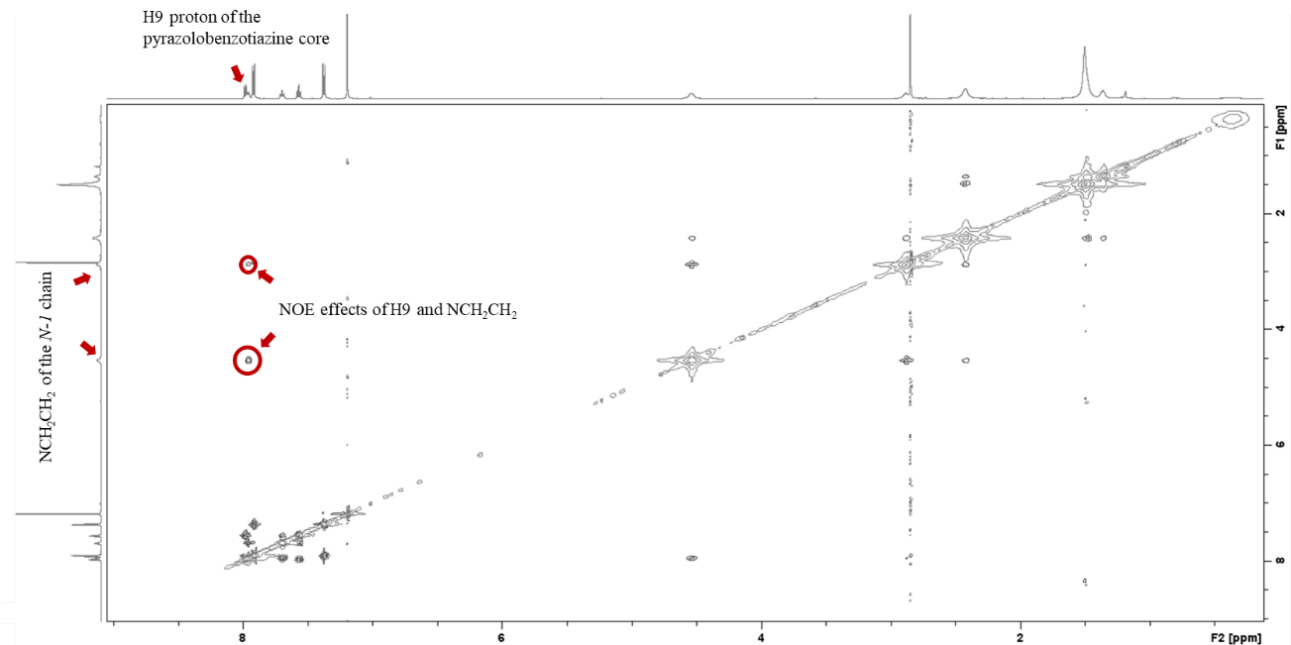
**

**Figure S4.** NOESY (400 MHz, CDCl_3_) spectrum of **18.** The red arrows indicate the protons that were involved in the interaction, while the red circle provides evidence of the NOE effect.


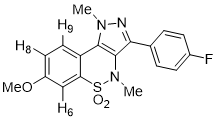


**
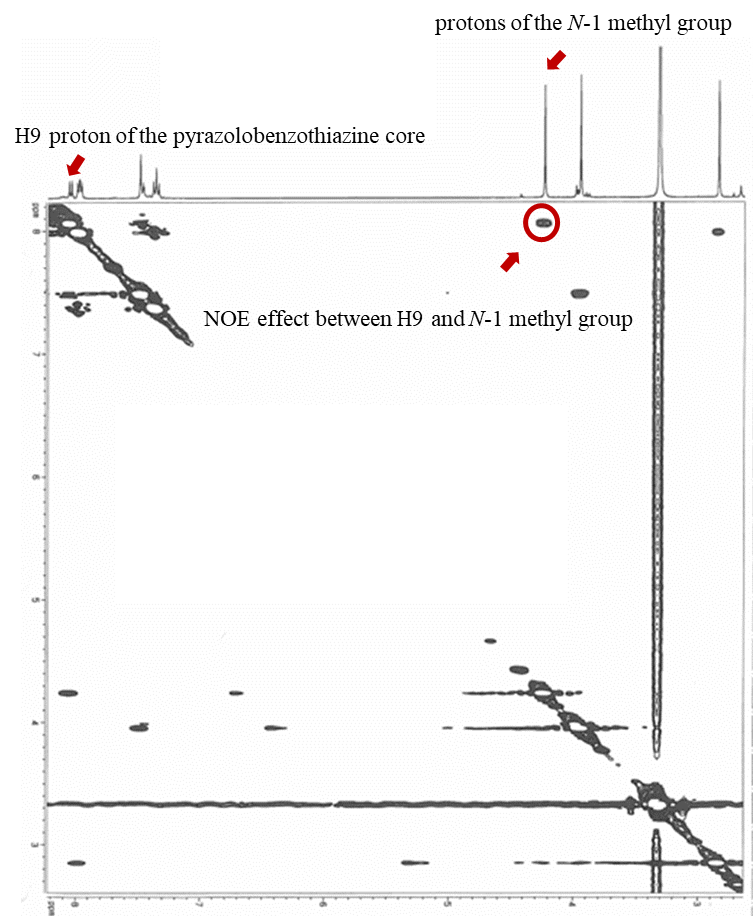
**

**Figure S5.** NOESY (400 MHz, DMSO-*d*_6_) spectrum of **22.** The red arrows indicate the protons that were involved in the interaction, while the red circle provides evidence of the NOE effect.


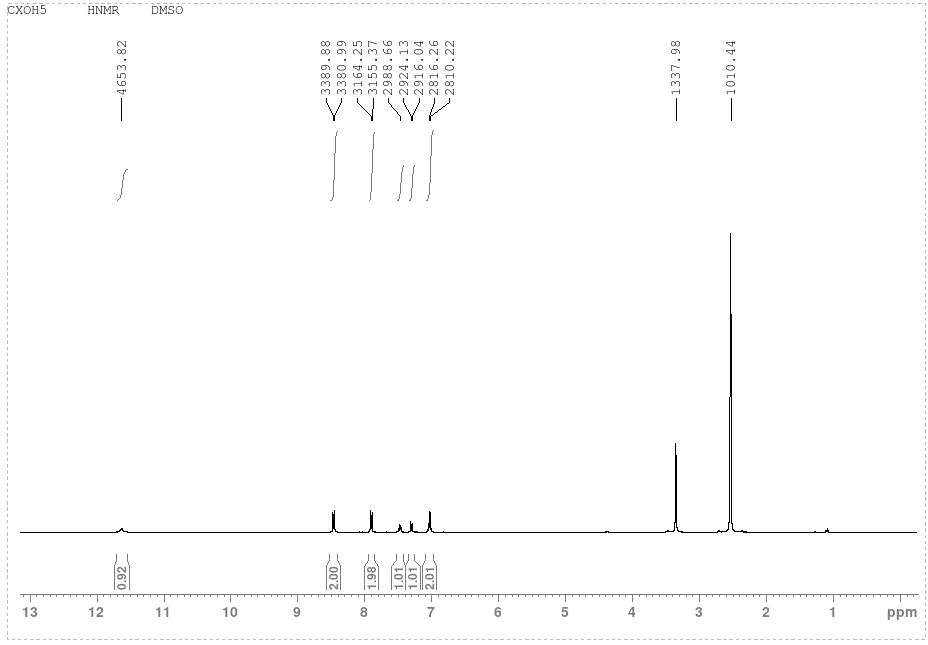


**Figure S6.** ^1^H NMR (200 MHz, DMSO-*d_6_*) spectrum of **5**.


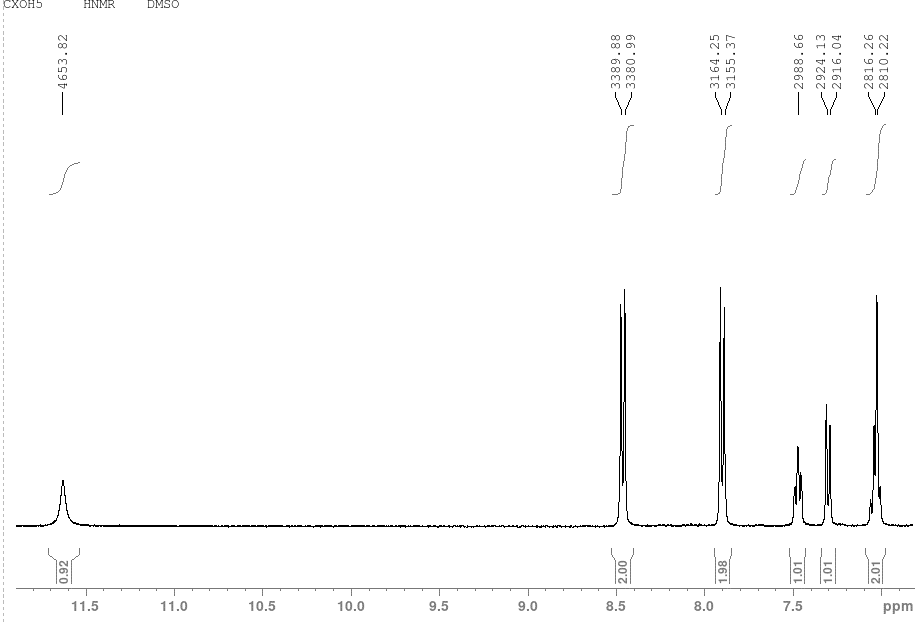


**Figure S7.** Zoom of the 7-12 ppm range of the ^1^H NMR spectrum of **5**.


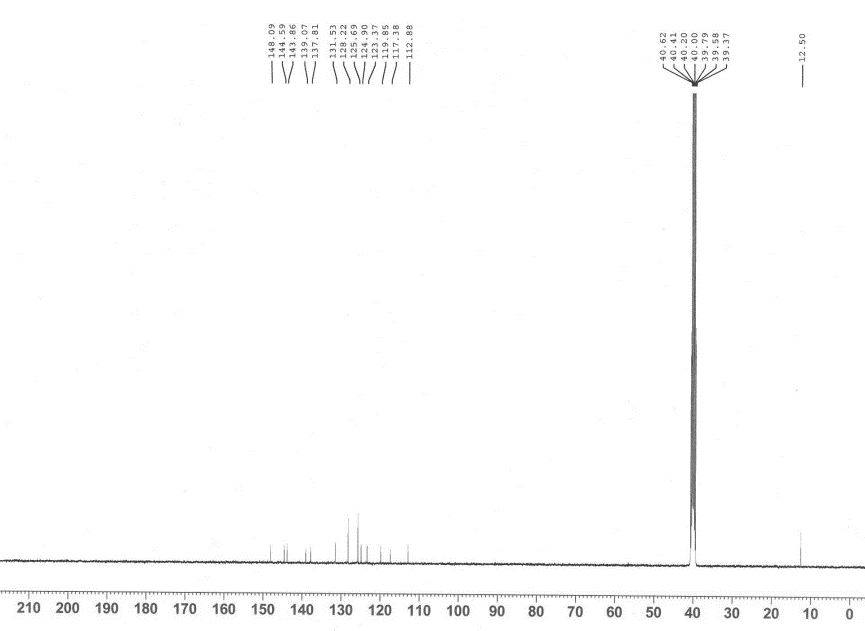


**Figure S8.** ^13^C NMR (101 MHz, DMSO-*d_6_*) spectrum of **5**.


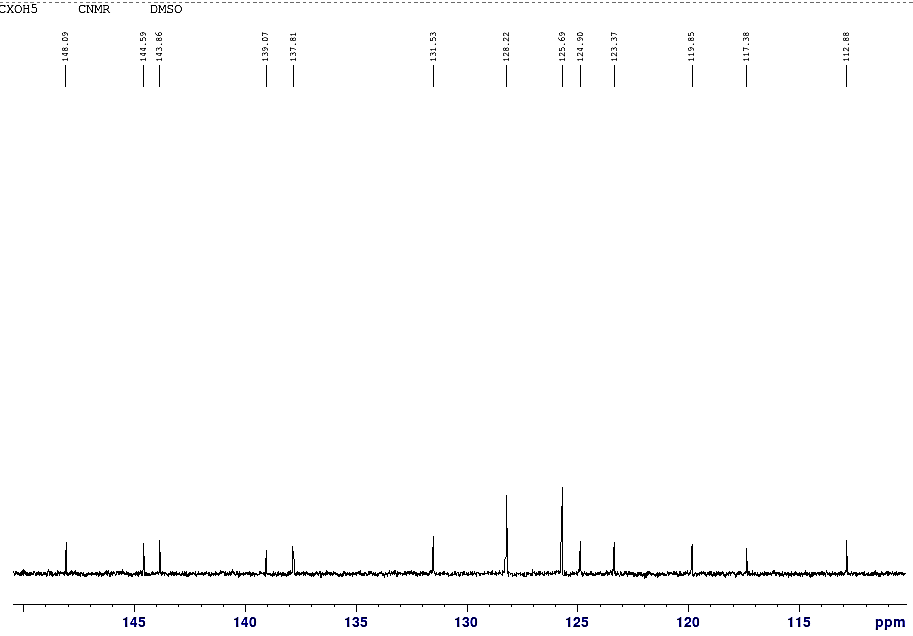


**Figure S9.** Zoom of the 110-150 ppm range of the ^13^C NMR spectrum of **5**.


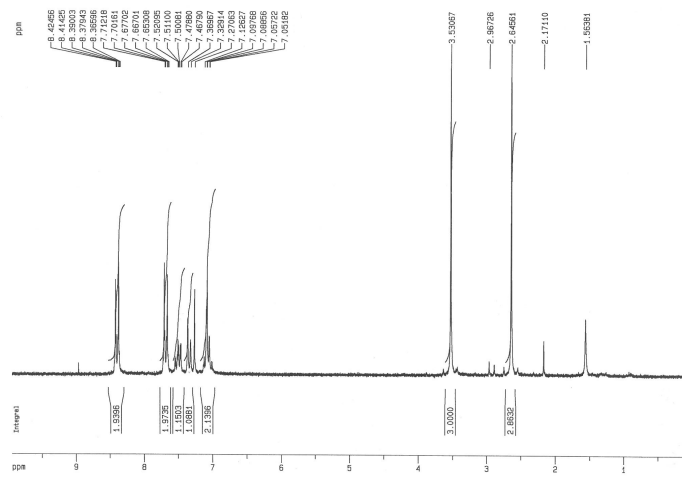


**Figure S10.** ^1^H NMR (200 MHz, CDCl_3_) spectrum of **7.**

**
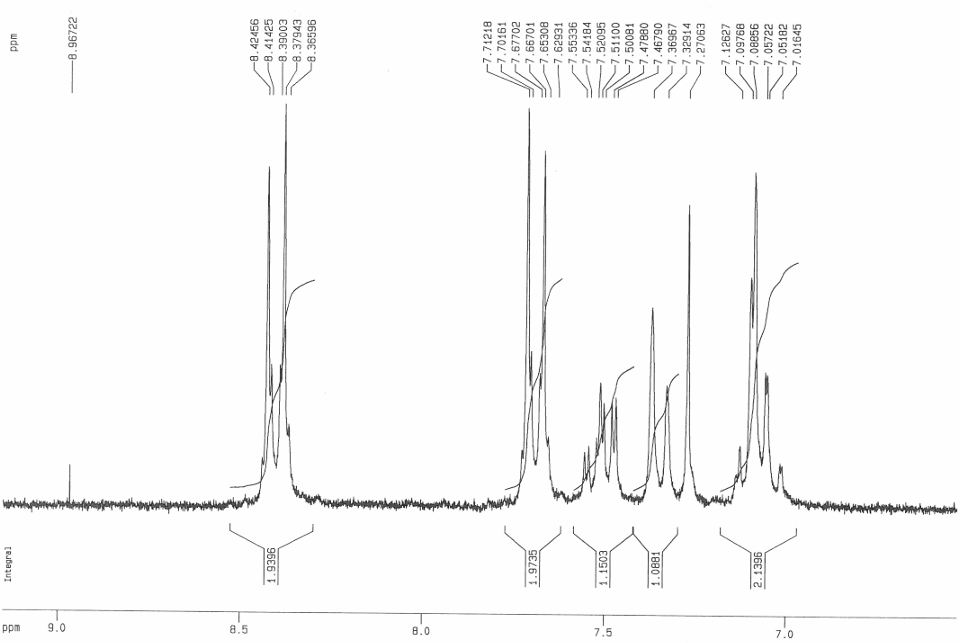
**

**Figure S11.** Zoom of the 6.5-9.2 ppm range of the ^1^H NMR spectrum of **7.**

**
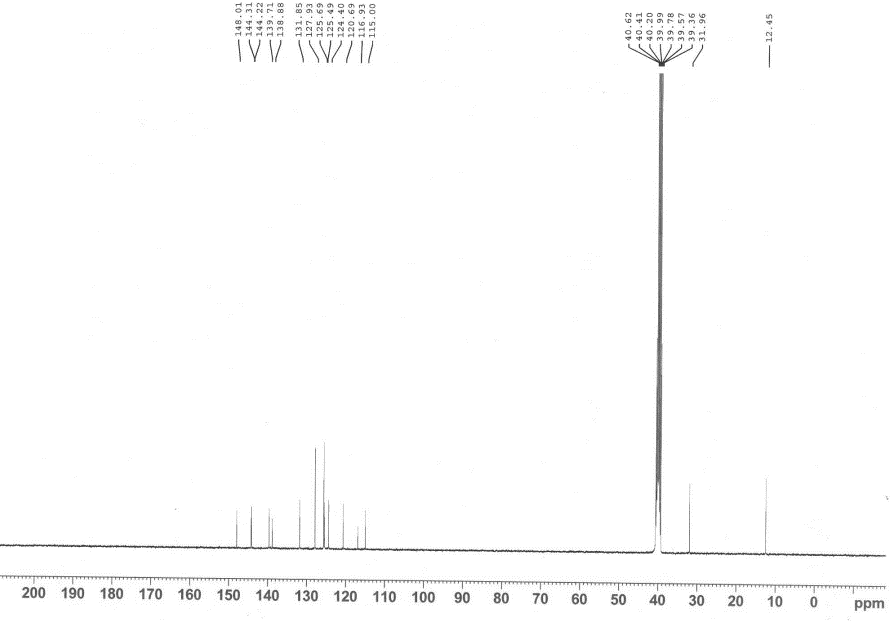
**

**Figure S12.** ^13^C NMR (101 MHz, CDCl_3_) spectrum of **7.**

**
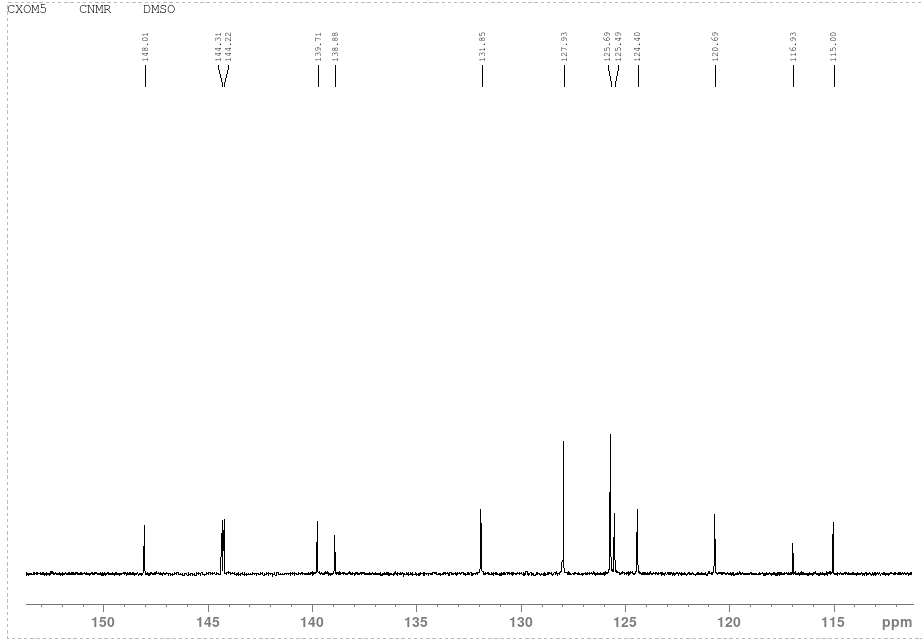
**

**Figure S13.** Zoom of the 110-155 ppm range of the ^13^C NMR spectrum of **7.**

**
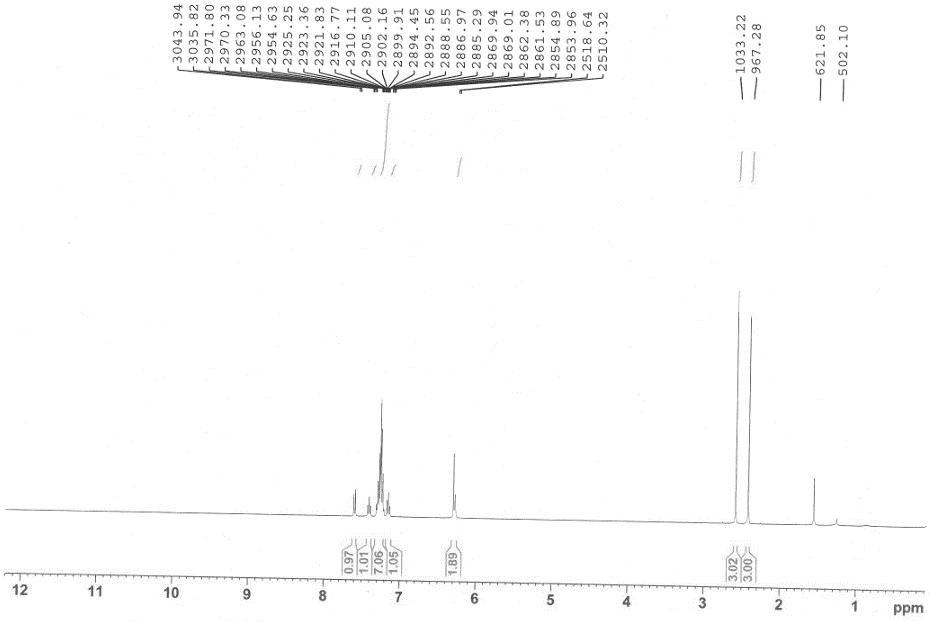
**

**Figure S14.** ^1^H NMR (400 MHz, CDCl_3_) spectrum of **8.**

**
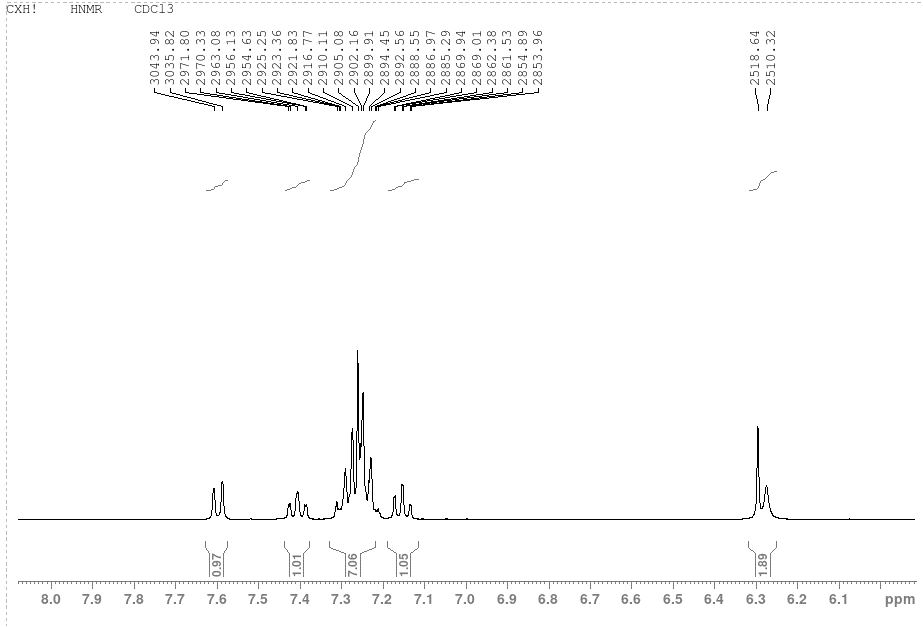
**

**Figure S15.** Zoom of the 5.9-8.1 ppm range of the ^1^H NMR spectrum of **8.**

**
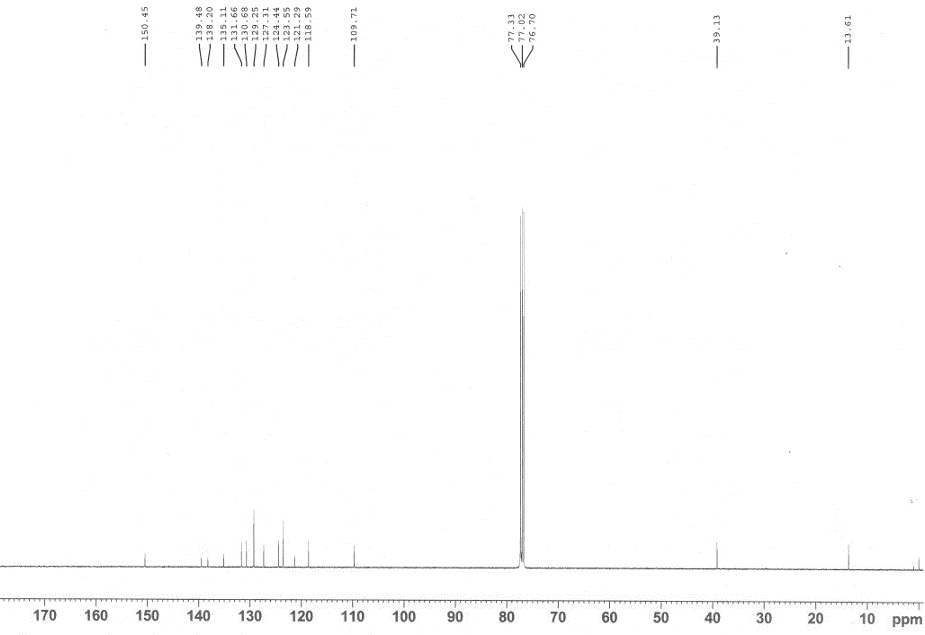
**

**Figure S16.** ^13^C NMR (101 MHz, CDCl_3_) spectrum of **8.**

**
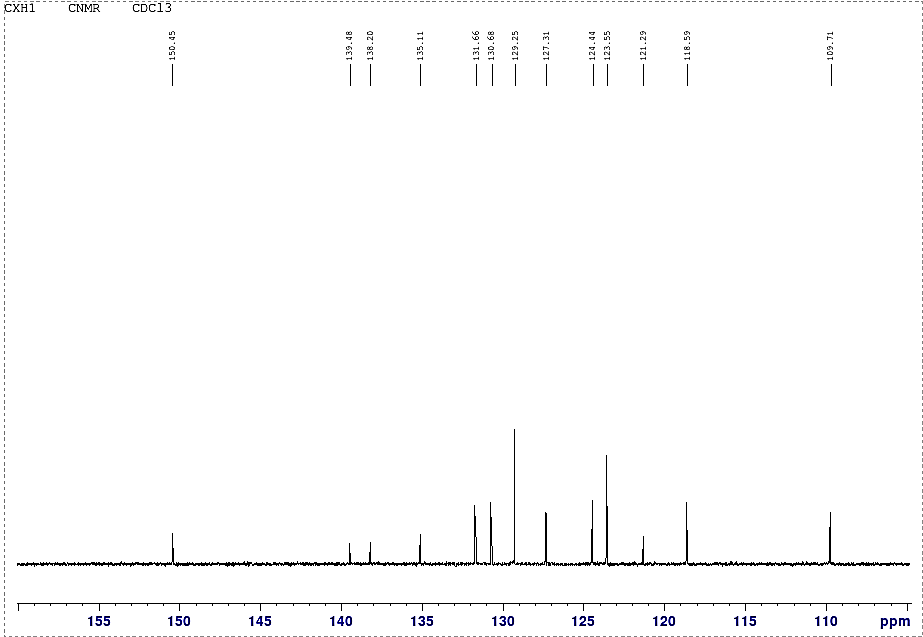
**

**Figure S17.** Zoom of the 105-160 ppm range of the ^13^C NMR spectrum of **8.**

**
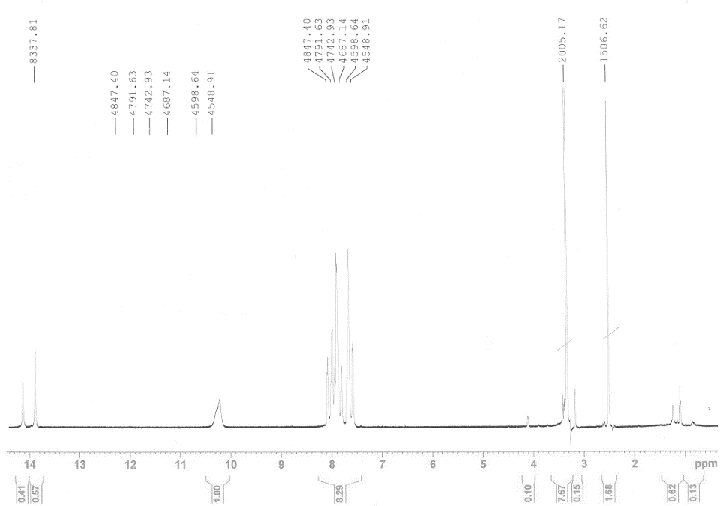
**

**Figure S18.** ^1^H NMR (400 MHz, DMSO-*d_6_*) spectrum of **9.**

**
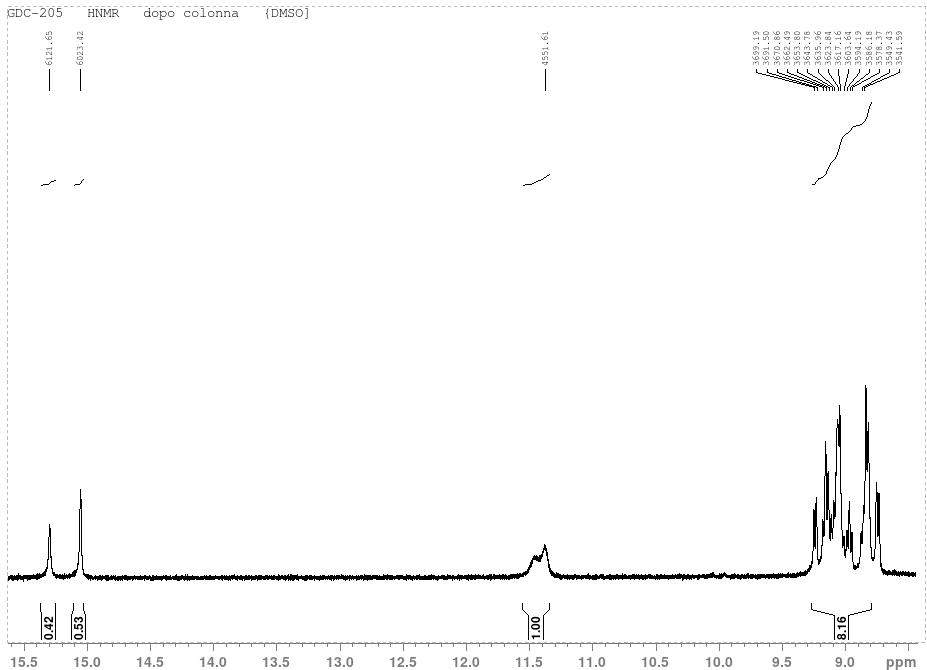
**

**Figure S19.** Zoom of the 8.5-15.5 ppm range of the ^1^H NMR spectrum of **9.**

**
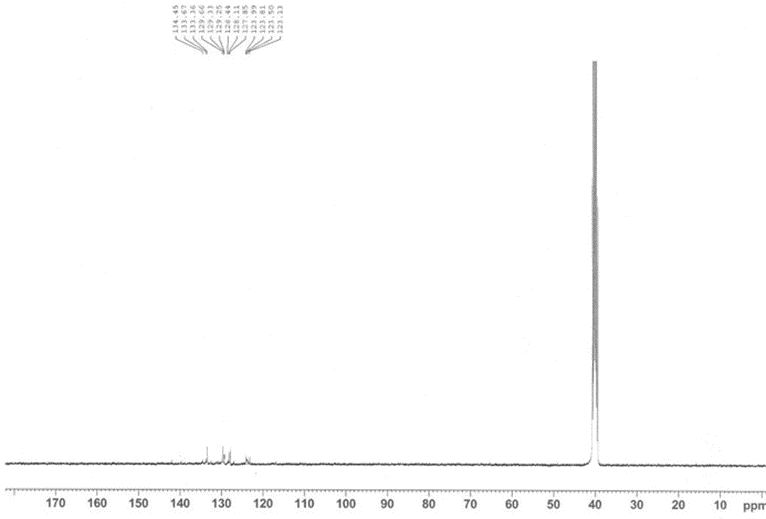
**

**Figure S20.** ^13^C NMR (101 MHz DMSO-*d_6_*) spectrum of **9.**

**
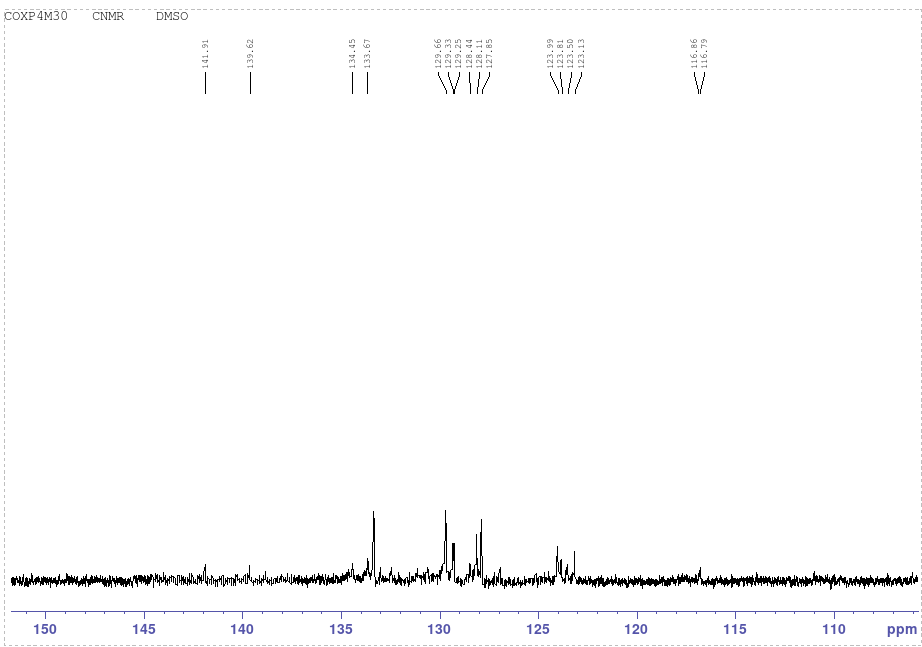
**

**Figure S21.** Zoom of the 105-150 ppm range of the ^13^C NMR spectrum of **9.**

**
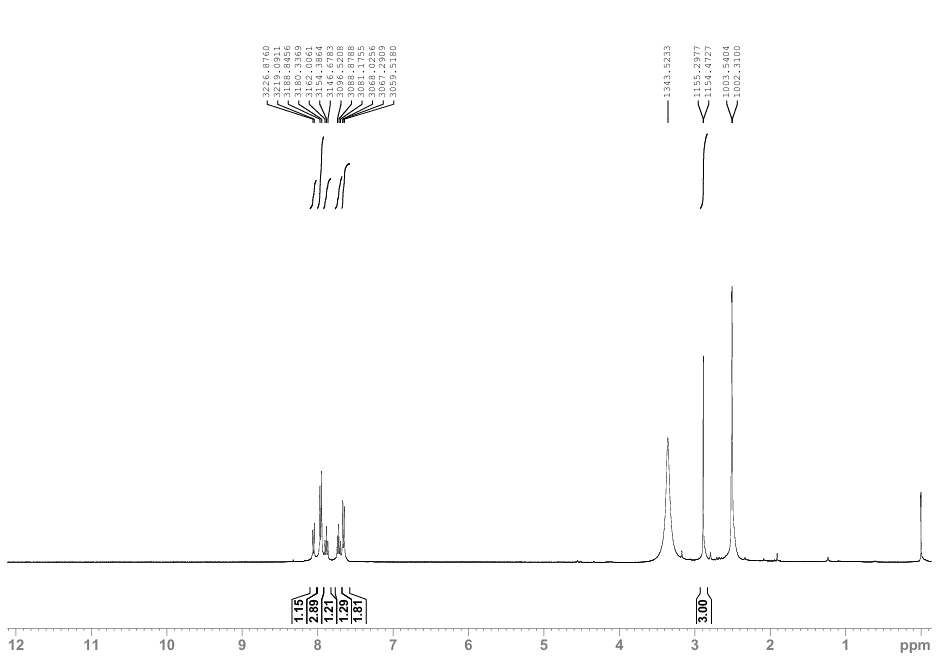
**

**Figure S22.** ^1^H NMR (400 MHz, DMSO-*d_6_*) spectrum of **10.**

**
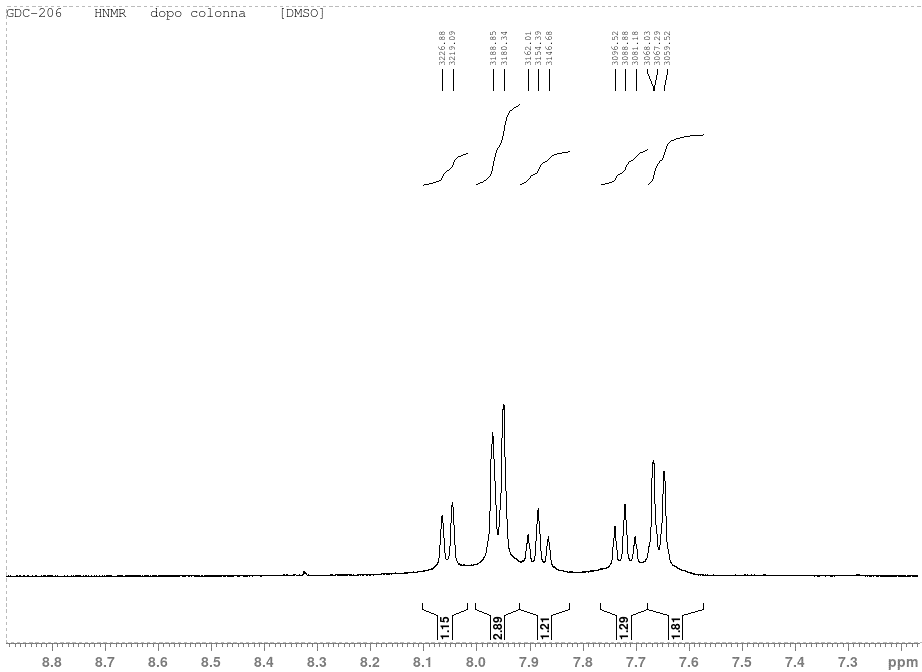
**

**Figure S23.** Zoom of the 7.2-8.8 ppm range of the ^1^H NMR spectrum of **10.**

**
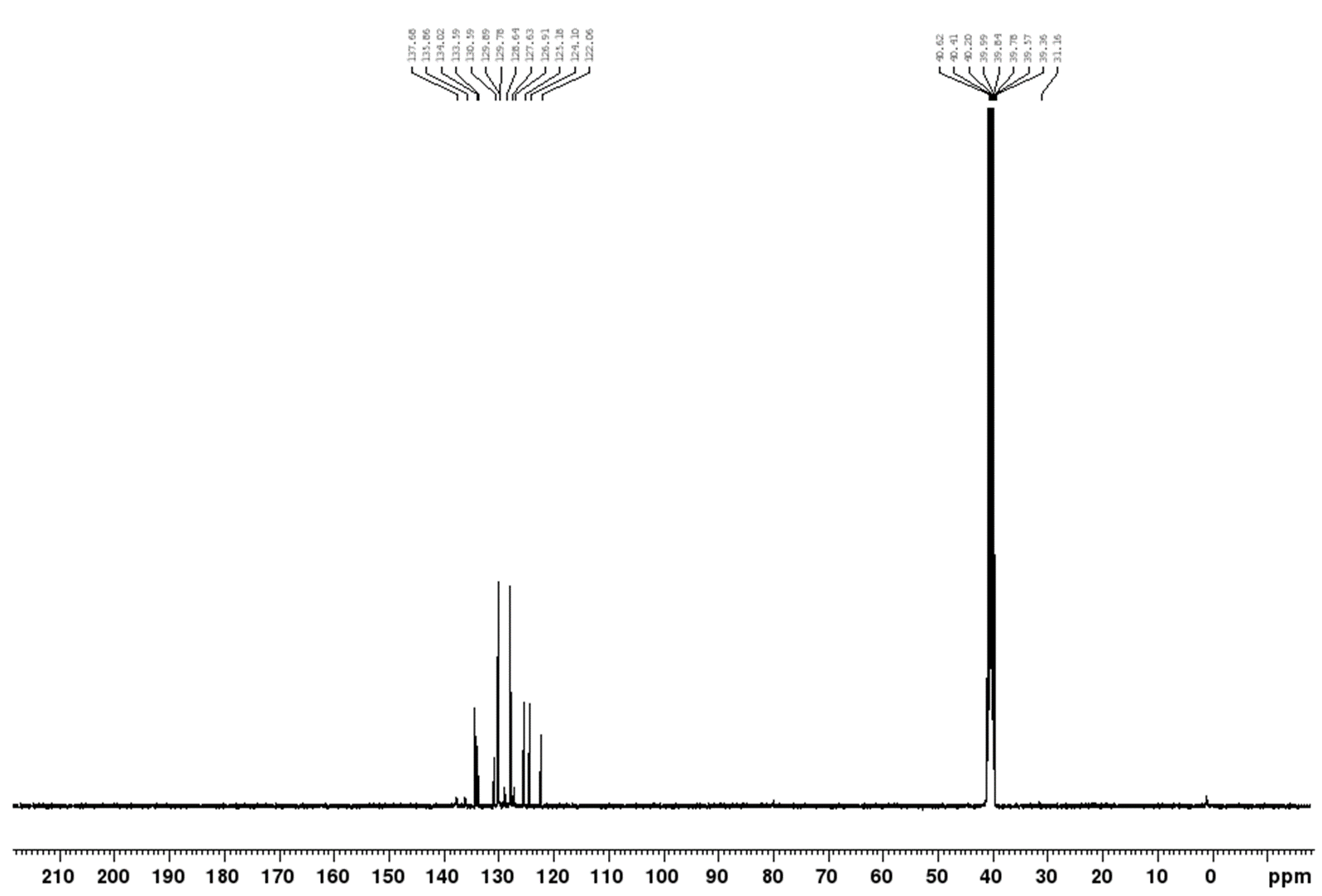
**

**Figure S24.** ^13^C NMR (101 MHz, DMSO-*d_6_*) spectrum of **10.**

**
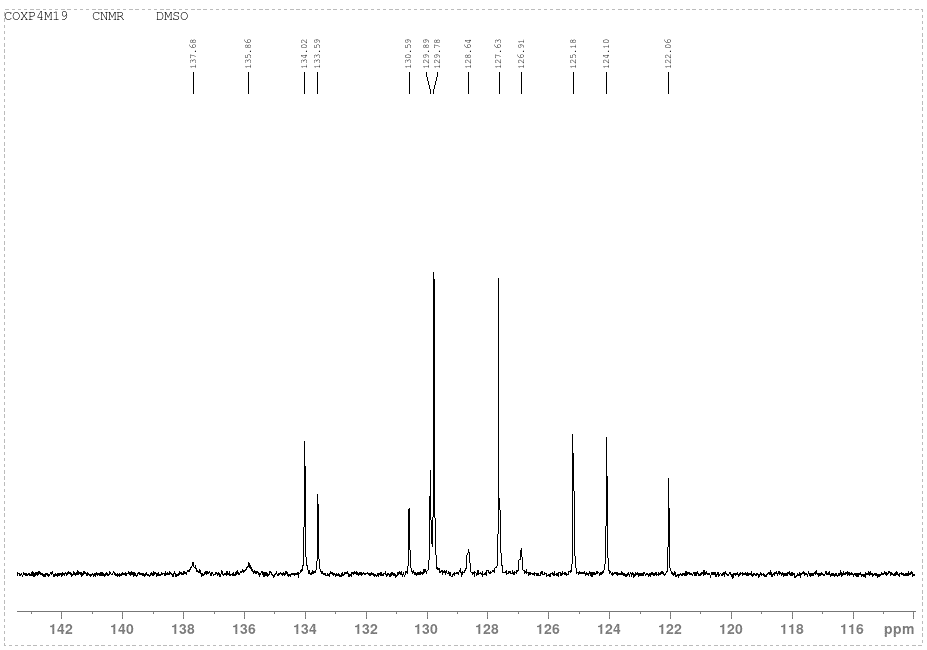
**

**Figure S25.** Zoom of the 114-144 ppm range of the ^13^C NMR spectrum of **10.**

**
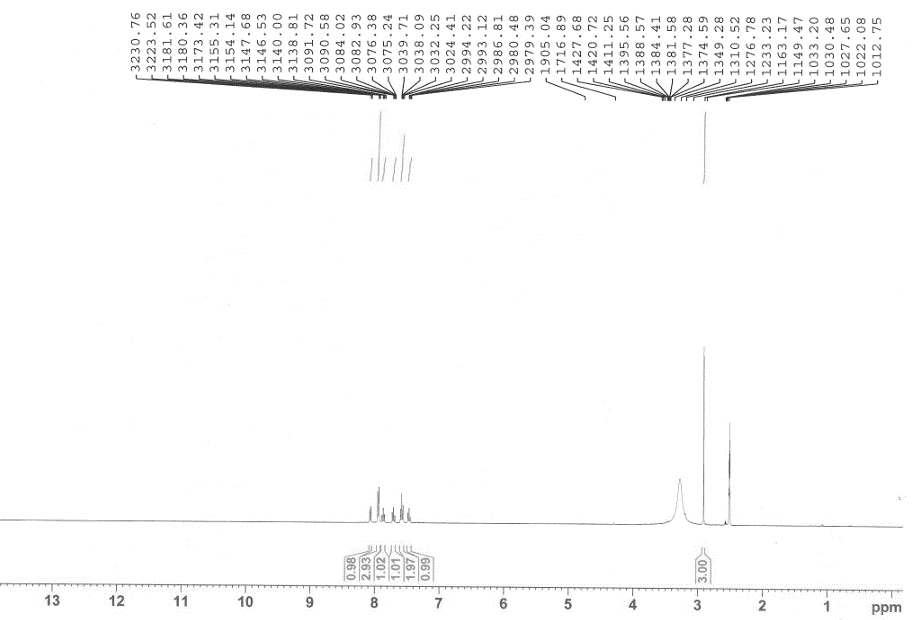
**

**Figure S26.** ^1^H NMR (400 MHz, DMSO-*d_6_*) spectrum of **11.**

**
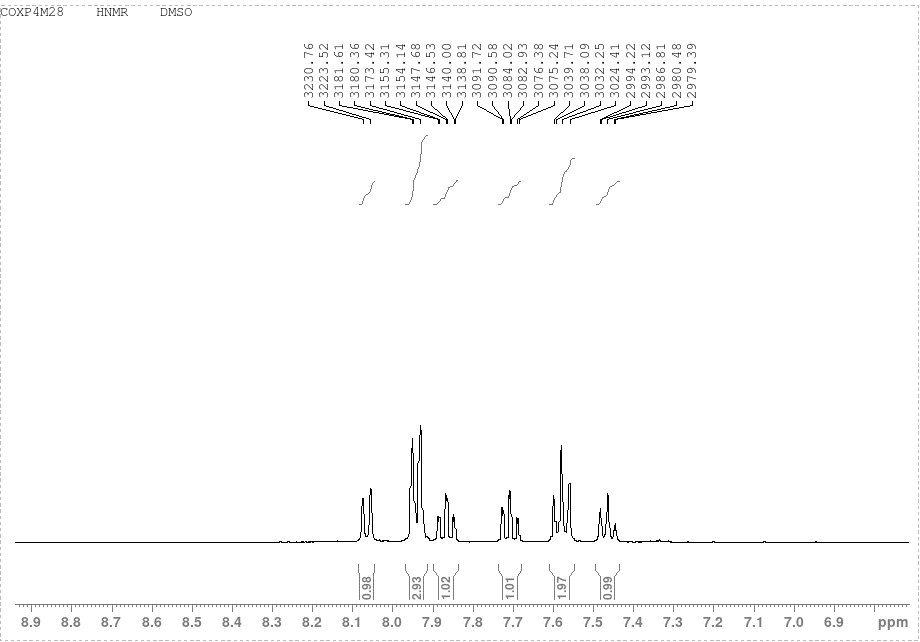
**

**Figure S27.** Zoom of the 6.7-8.9 ppm range of the ^1^H NMR spectrum of **11.**

**
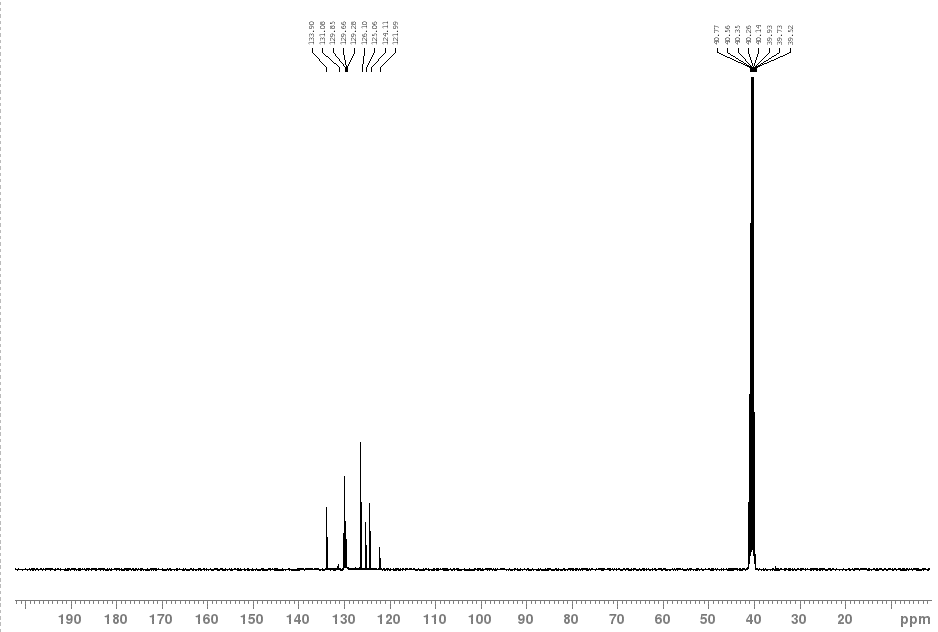
**

**Figure S28.** ^13^C NMR (101 MHz, DMSO-*d_6_*) spectrum of **11.**

**
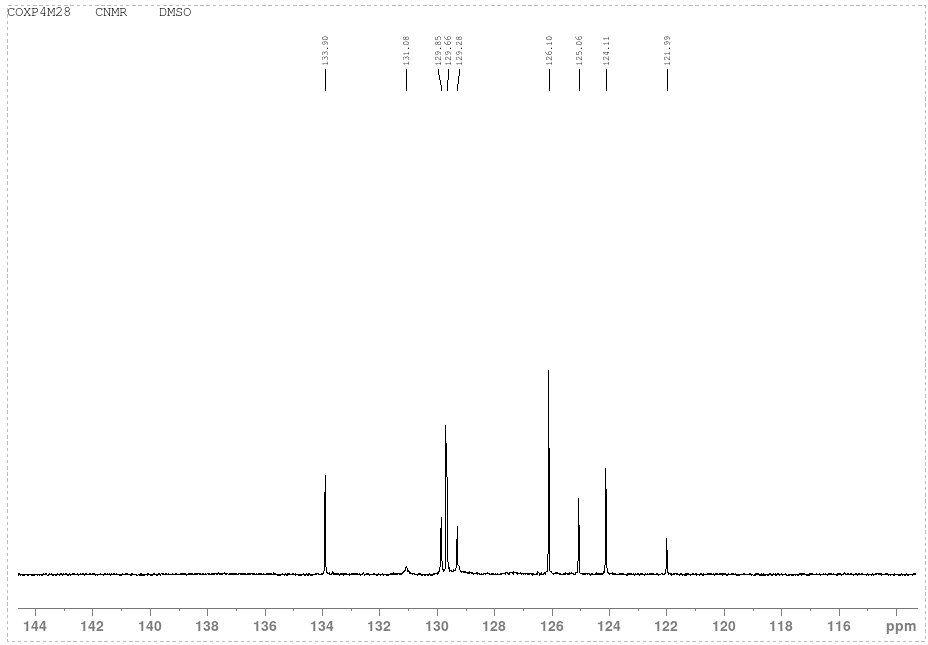
**

**Figure S29.** Zoom of the 114-144 ppm range of the ^13^C NMR spectrum of **11.**

**
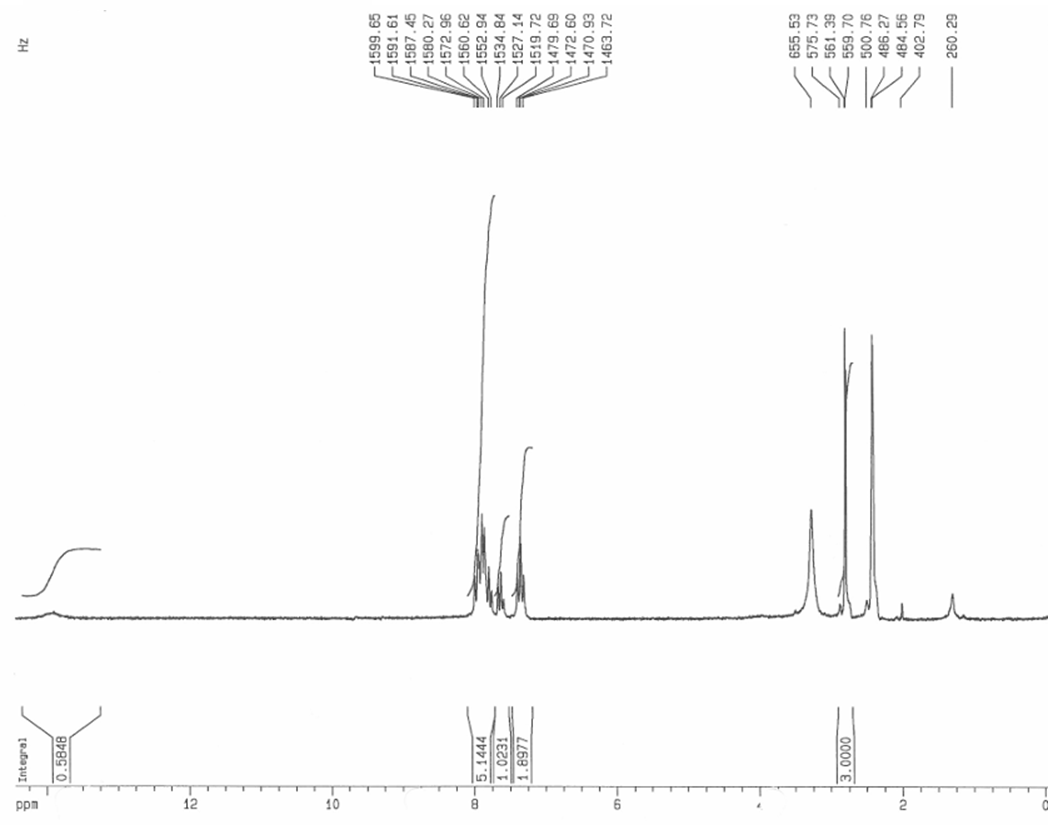
**

**Figure S30.** ^1^H NMR (200 MHz, DMSO-*d_6_*) spectrum of **12.**

**
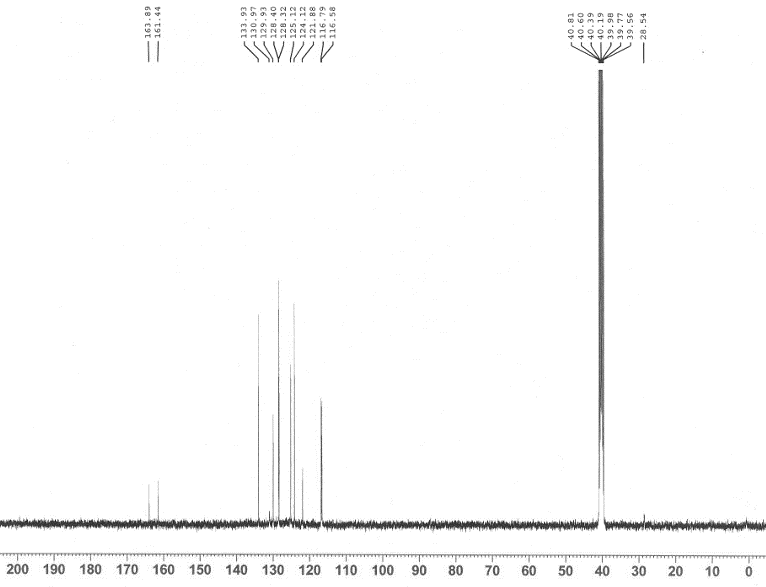
**

**Figure S31.** ^13^C NMR (101 MHz DMSO-*d_6_*) spectrum of **12.**

**
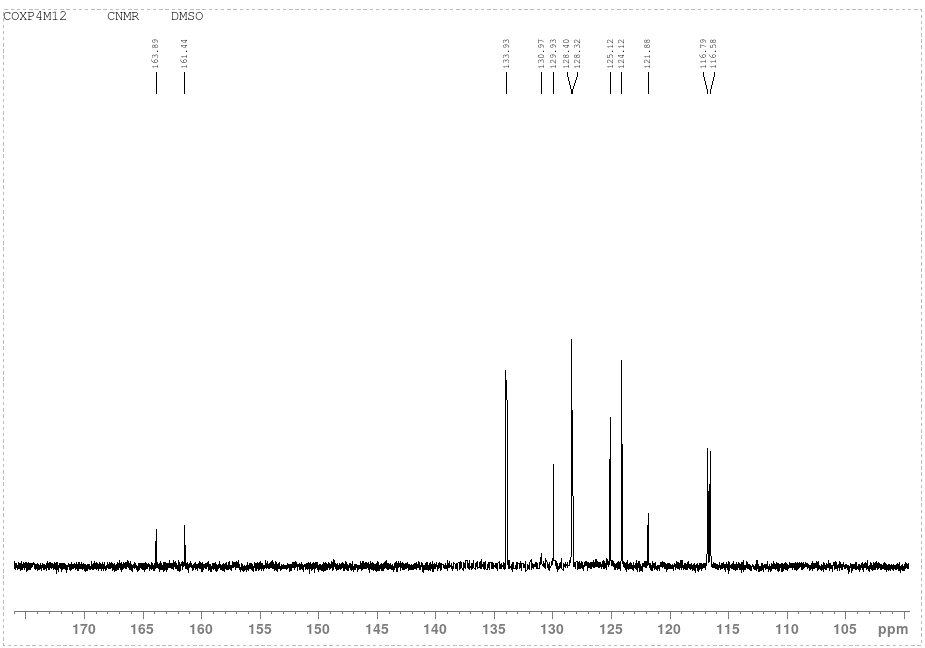
**

**Figure S32.** Zoom of the 100-170 ppm range of the ^13^C NMR spectrum of **12.**

**
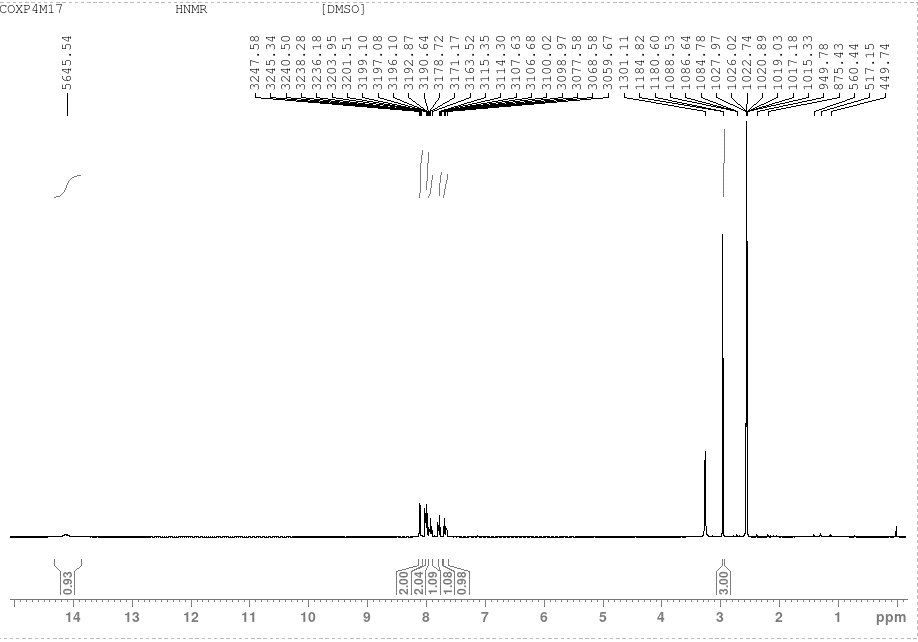
**

**Figure S33.** ^1^H NMR (400 MHz, Acetone) spectrum of **13.**

**
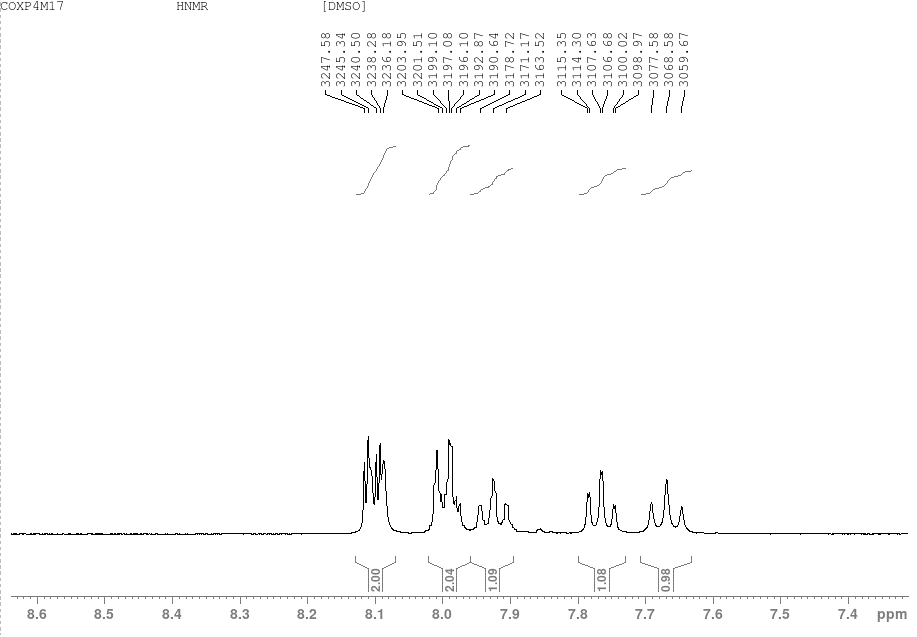
**

**Figure S34.** Zoom of the 7.3-8.8 ppm range of the ^1^H NMR spectrum of **13.**

**
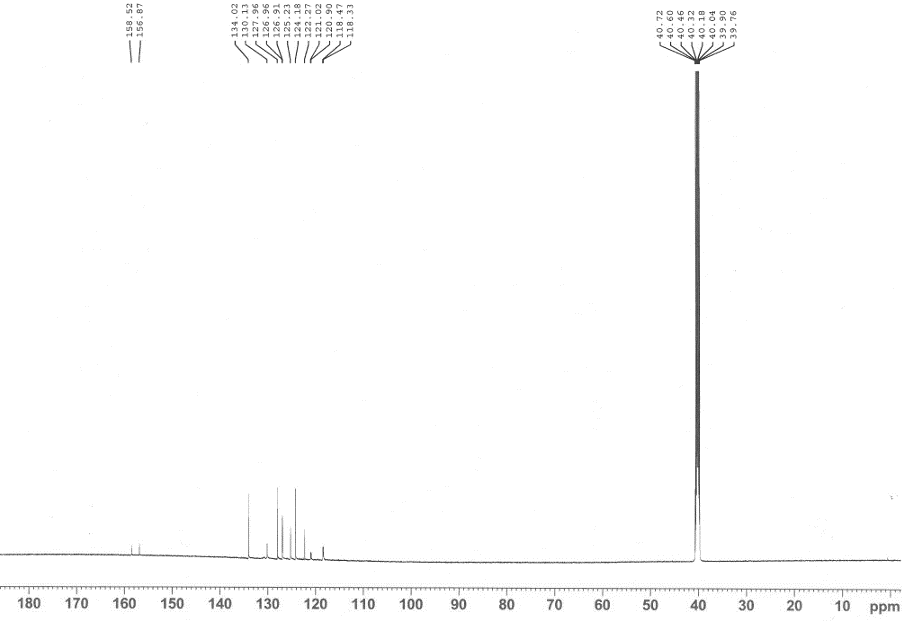
**

**Figure S35.** ^13^C NMR (101 MHz, Acetone) spectrum of **13.**

**
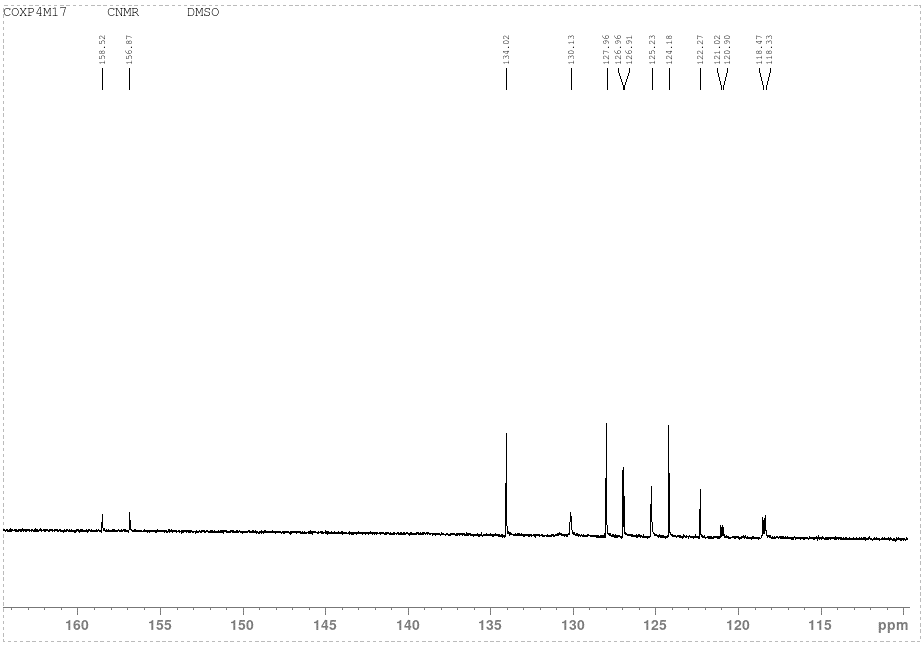
**

**Figure S36.** Zoom of the 110-165 ppm range of the ^13^C NMR spectrum of **13.**

**
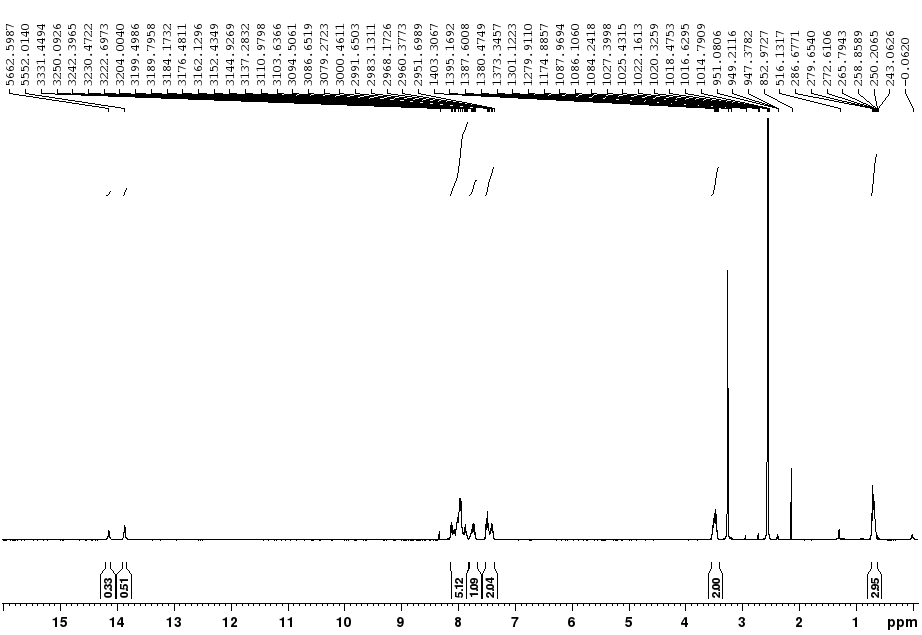
**

**Figure S37.** ^1^H NMR (400 MHz, DMSO-*d_6_*) spectrum of **14.**

**
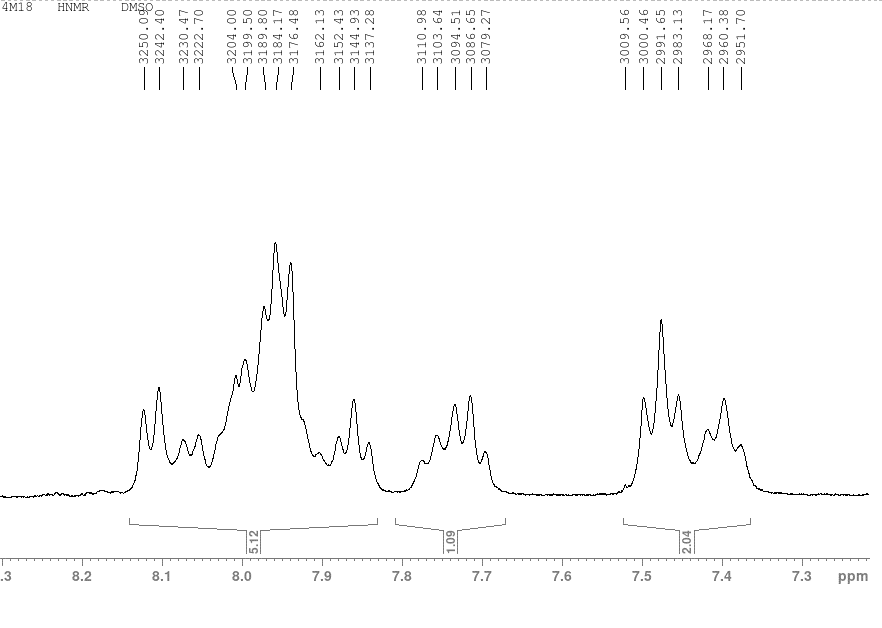
**

**Figure S38.** Zoom of the 7.2-8.3 ppm range of the ^1^H NMR spectrum of **14.**

**
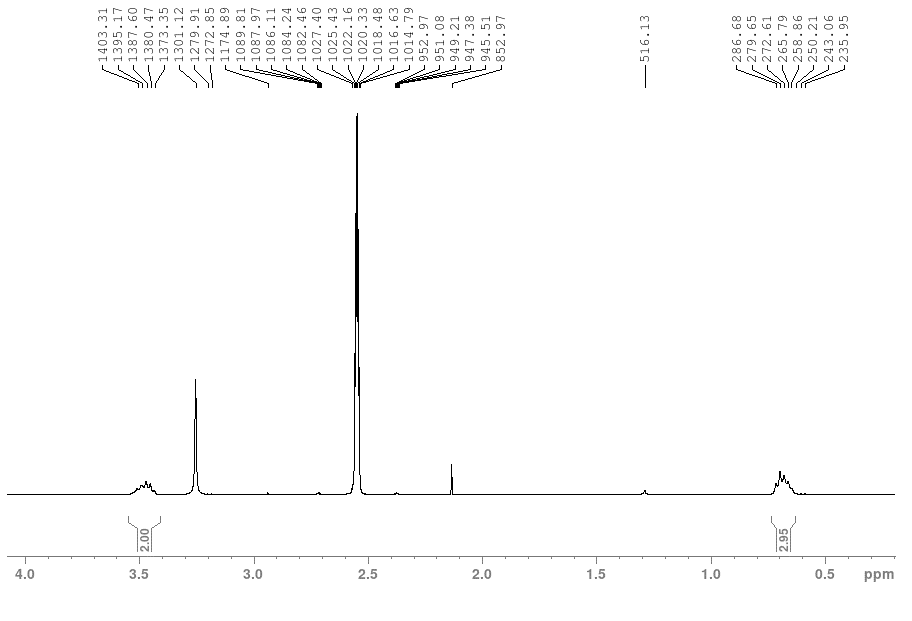
**

**Figure S39.** Zoom of the 0.1-4.1 ppm range of the ^1^H NMR spectrum of **14.**

**
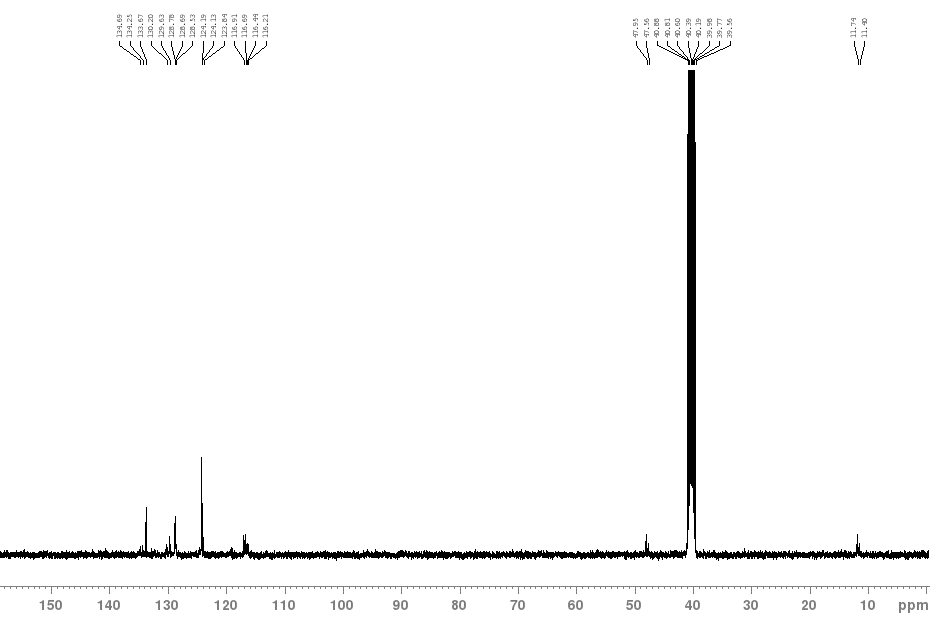
**

**Figure S40.** ^13^C NMR (101 MHz, DMSO-*d_6_*) spectrum of **14.**

**
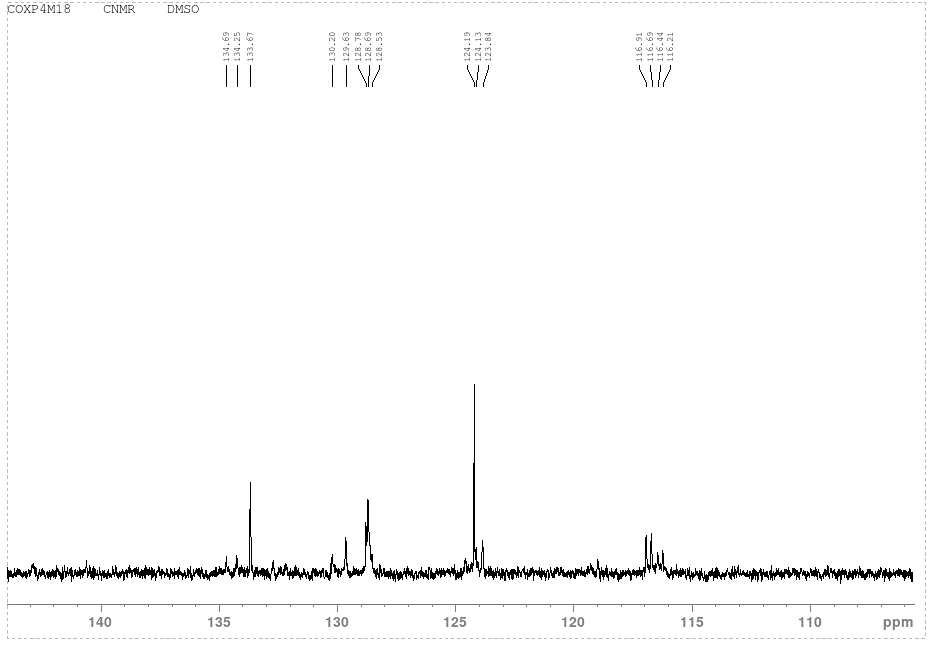
**

**Figure S41.** Zoom of the 105-145 ppm range of the ^13^C NMR spectrum of **14.**

**
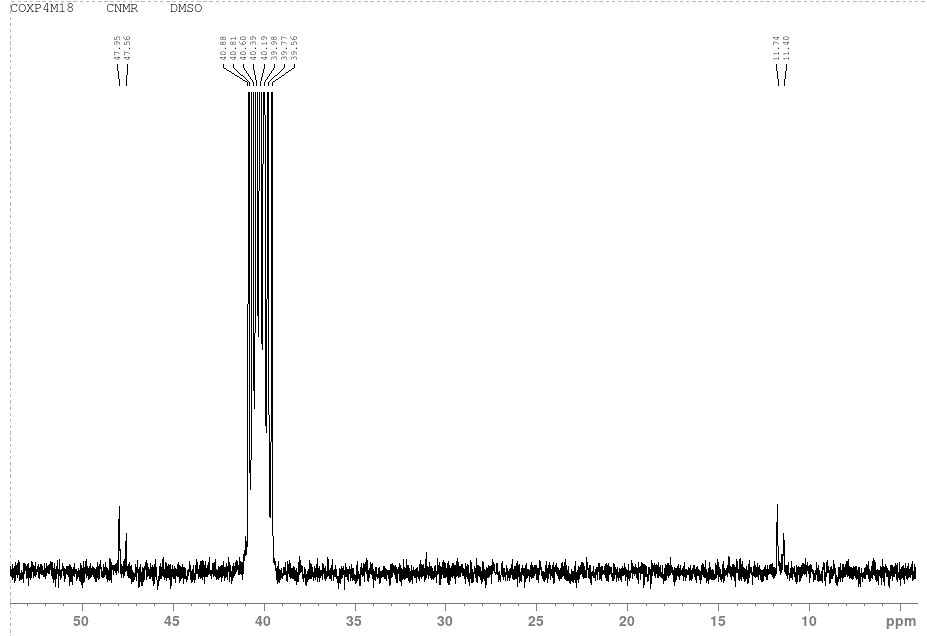
**

**Figure S42.** Zoom of the 5-55 ppm range of the ^13^C NMR spectrum of **14.**

**
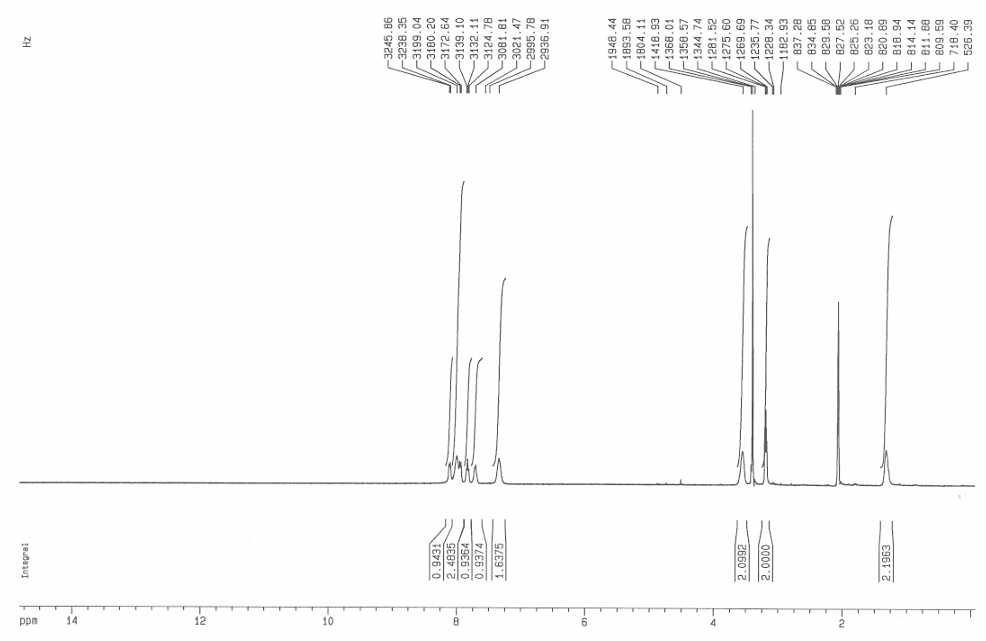
**

**Figure S43.** ^1^H NMR (400 MHz, Acetone) spectrum of **15.**

**
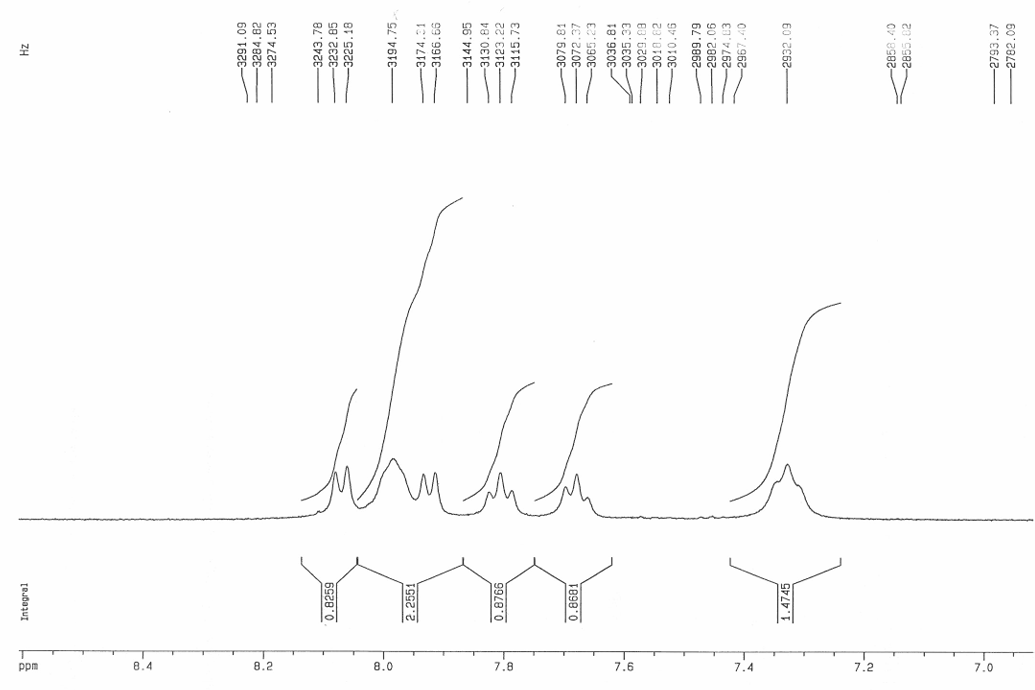
**

**Figure S44.** Zoom of the 7.0-8.6 ppm range of the ^1^H NMR spectrum of **15.**

**
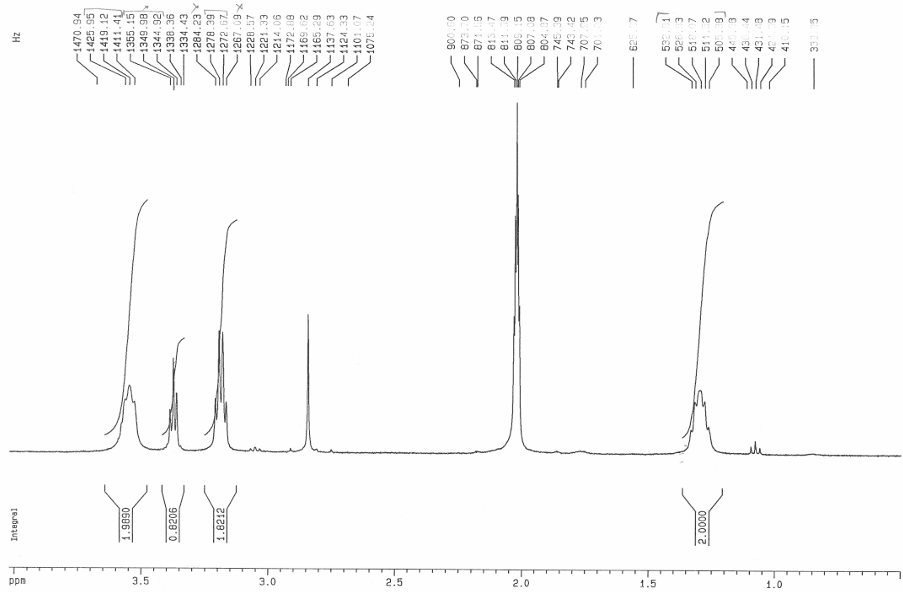
**

**Figure S45.** Zoom of the 0.1-4.0 ppm range of the ^1^H NMR spectrum of **15.**

**
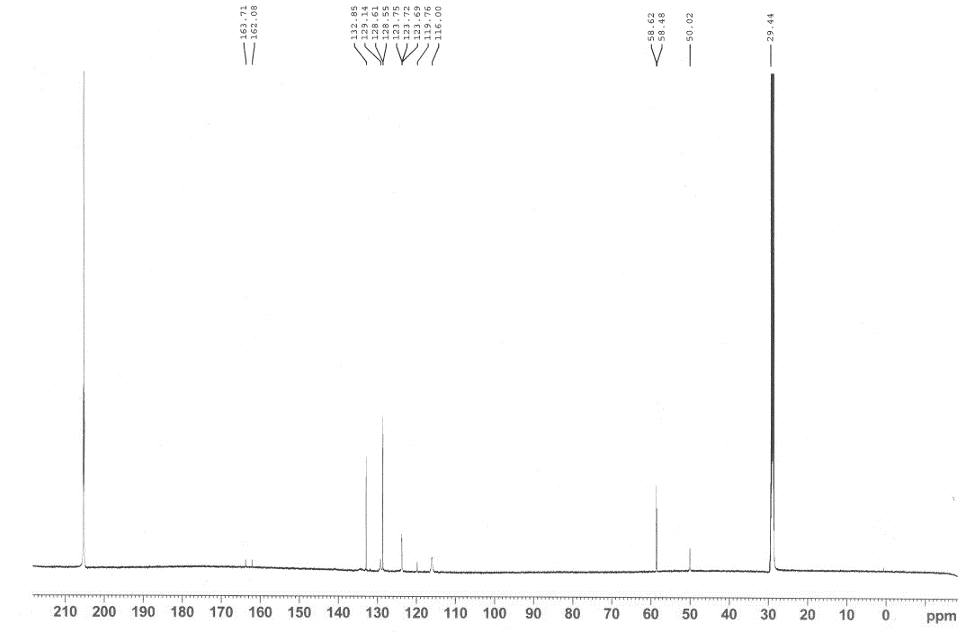
**

**Figure S46.** ^13^C NMR (150 MHz, Acetone) spectrum of **15.**

**
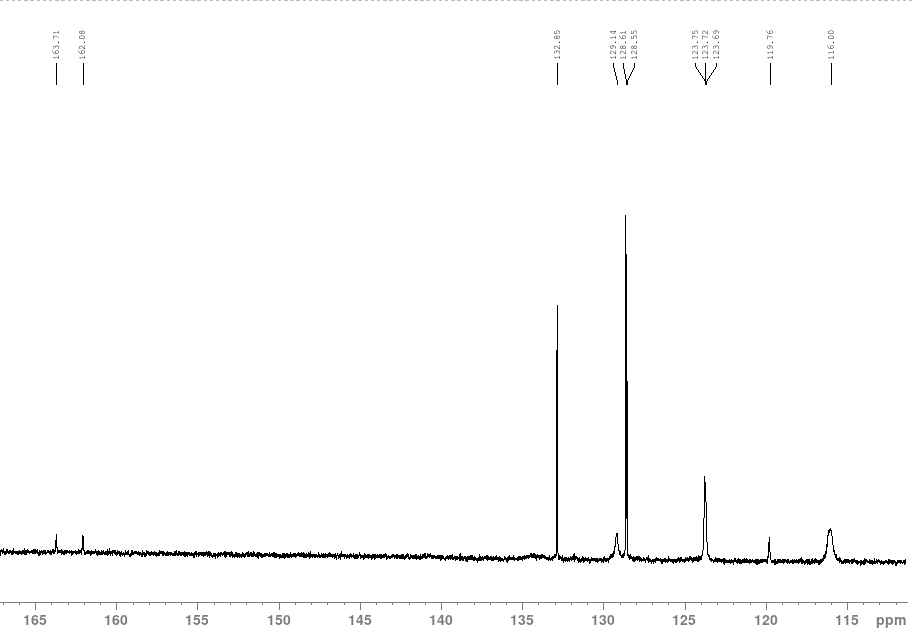
**

**Figure S47.** Zoom of the 110-166 ppm range of the ^13^C NMR spectrum of **15.**

**
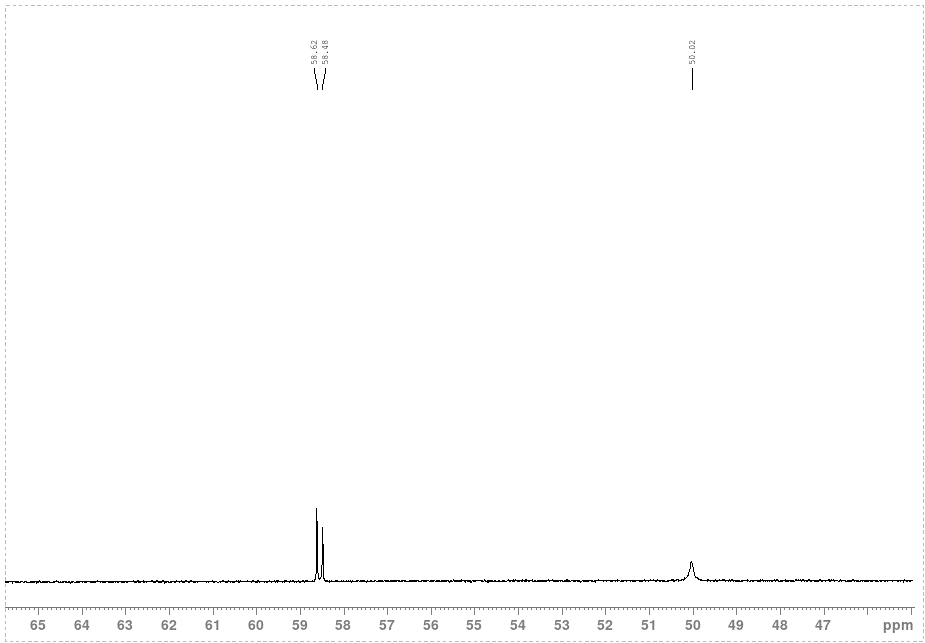
**

**Figure S48.** Zoom of the 45-65 ppm range of the ^13^C NMR spectrum of **15.**

**
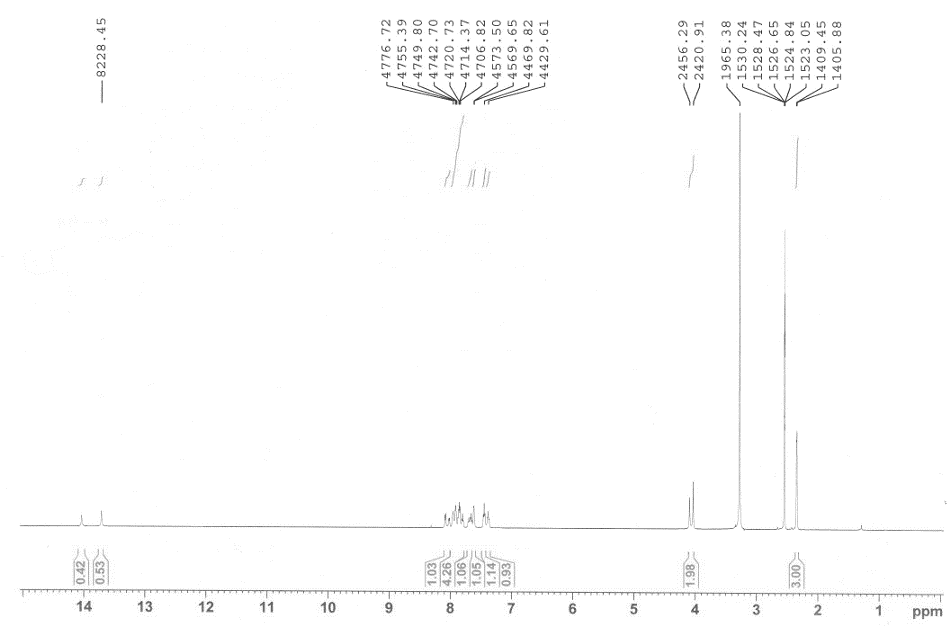
**

**Figure S49.** ^1^H NMR (600 MHz, DMSO-*d_6_*) spectrum of **16.**

**
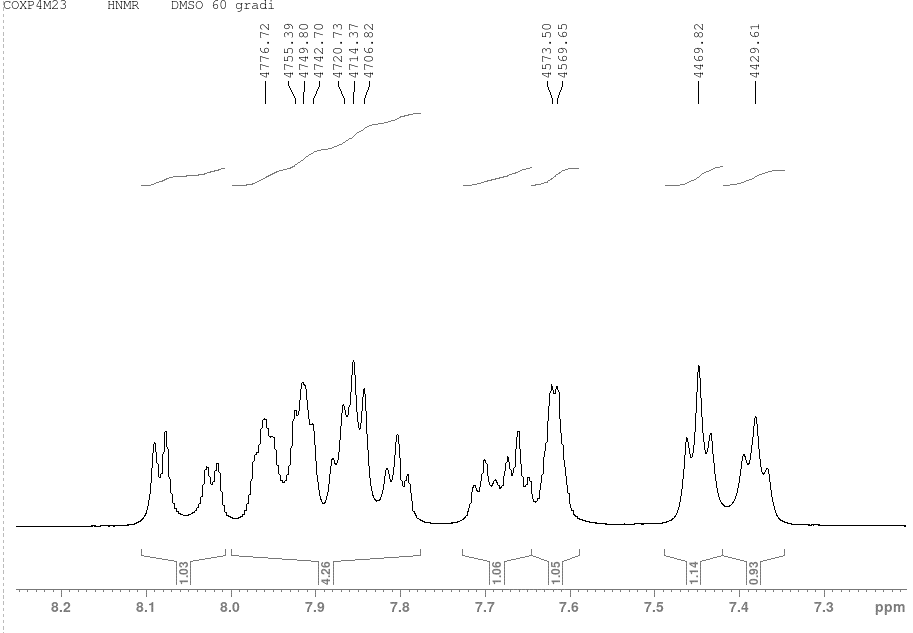
**

**Figure S50.** Zoom of the 7.2-8.2 ppm range of the ^1^H NMR spectrum of **16.**

**
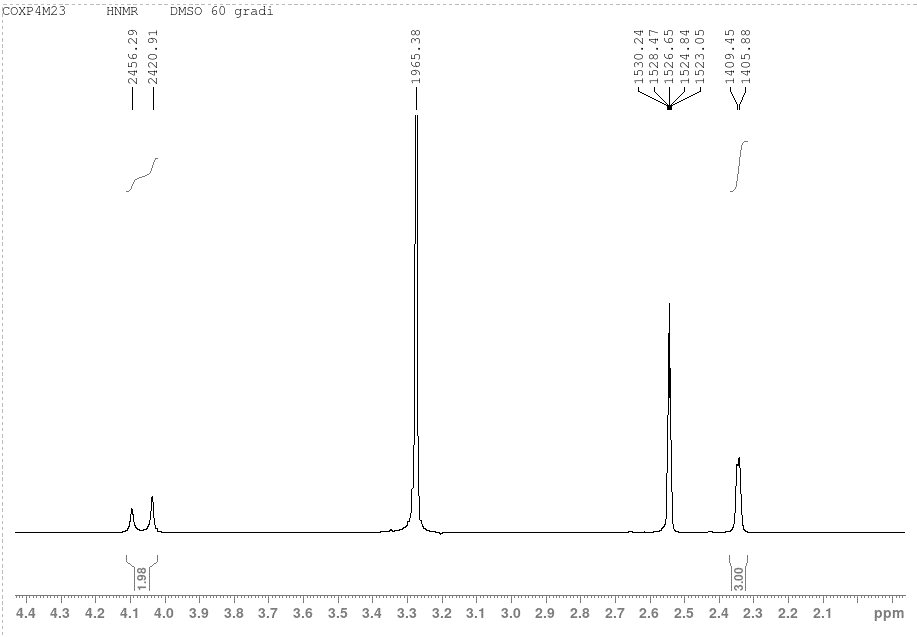
**

**Figure S51.** Zoom of the 1.9-4.4 ppm range of the ^1^H NMR spectrum of **16.**

**
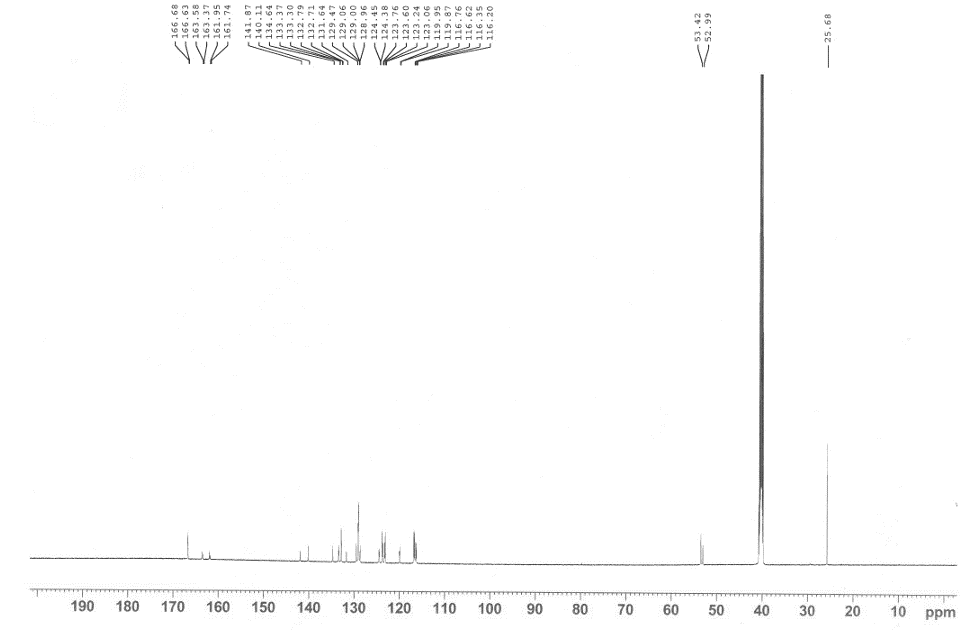
**

**Figure S52.** ^13^C NMR (150 MHz, DMSO-*d_6_*) spectrum of **16.**

**
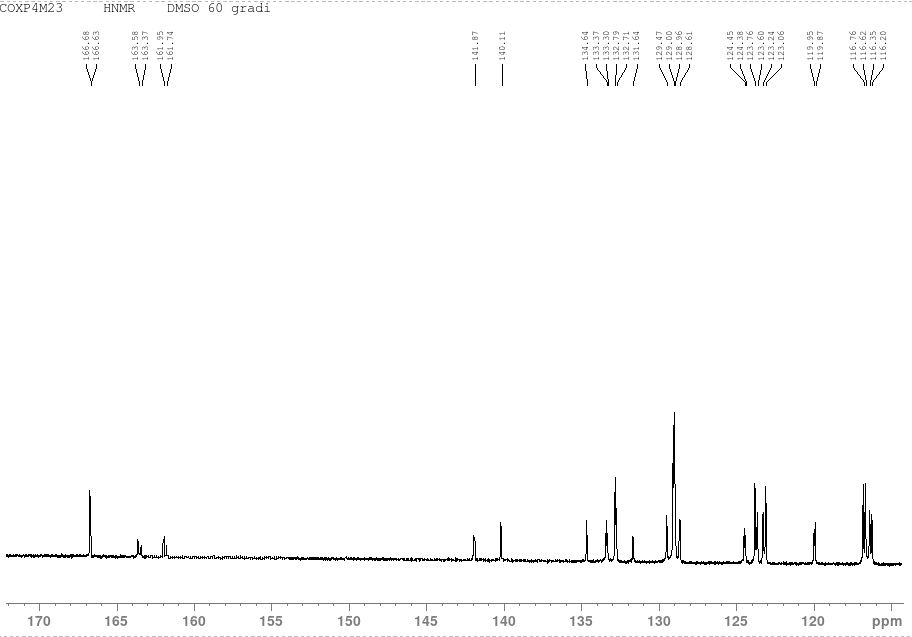
**

**Figure S53.** Zoom of the 115-170 ppm range of the ^13^C NMR spectrum of **16.**

**
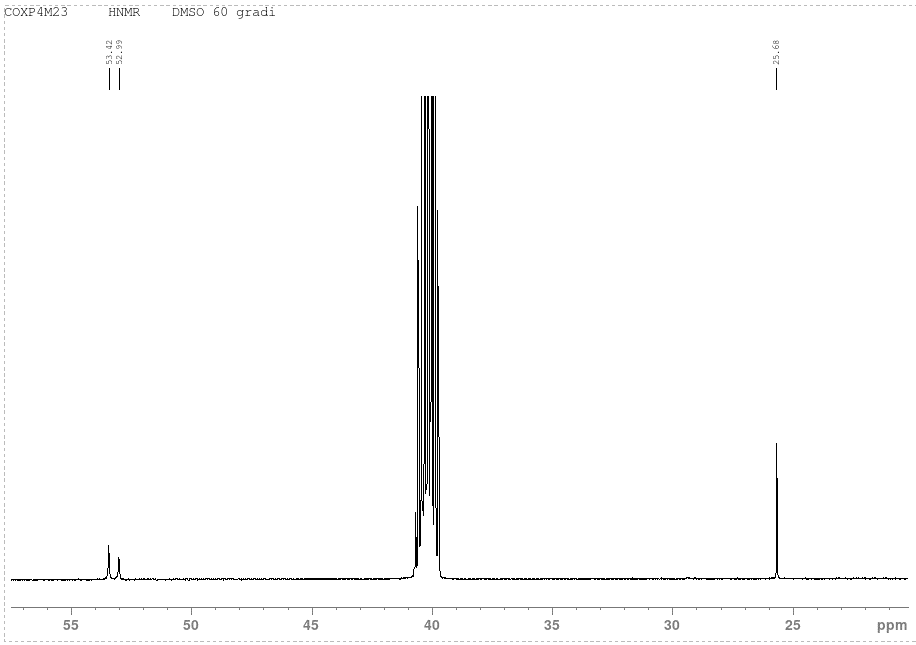
**

**Figure S54.** Zoom of the 20-58 ppm range of the ^13^C NMR spectrum of **16.**

**
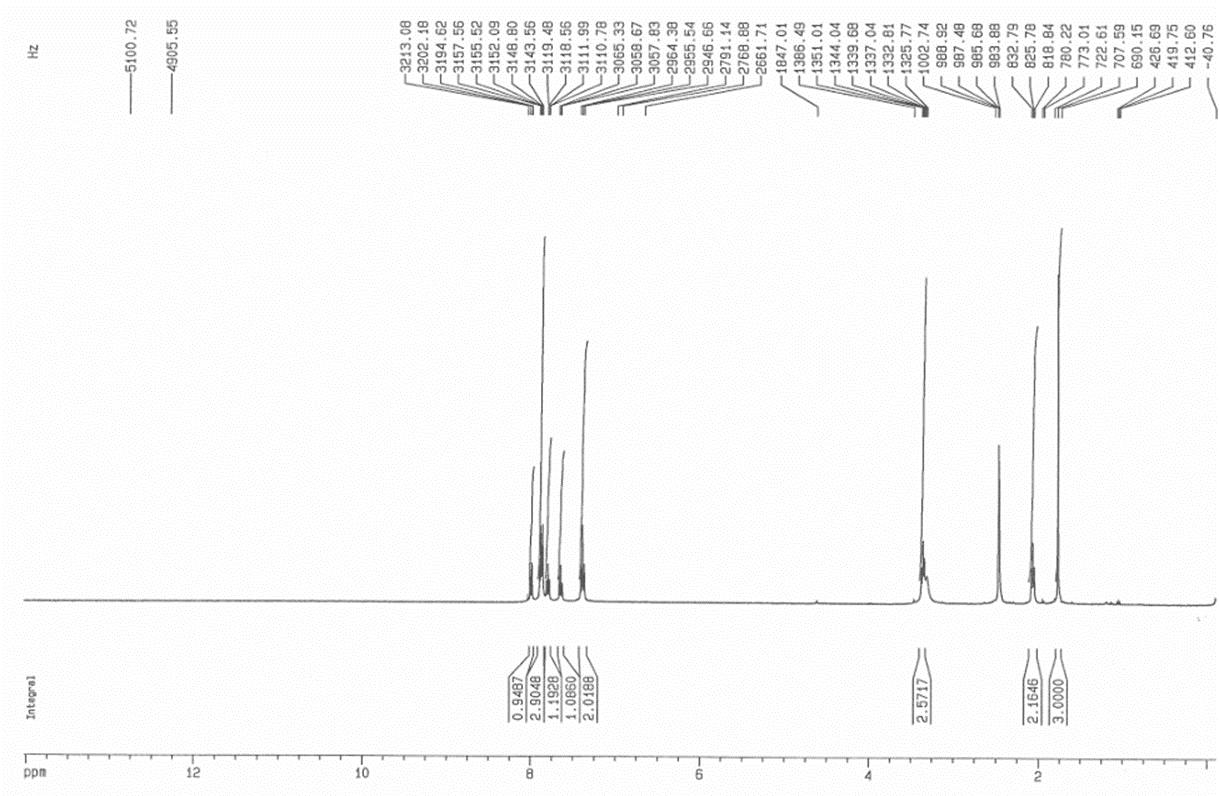
**

**Figure S55.** ^1^H NMR (400 MHz, DMSO-*d_6_*) spectrum of **17.**

**
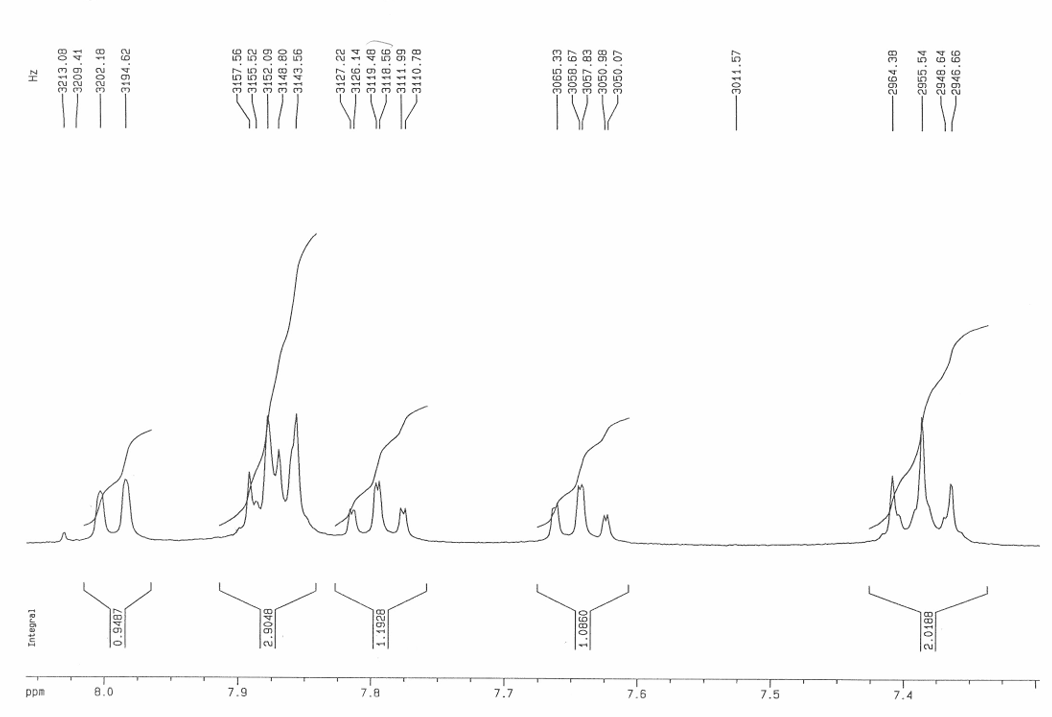
**

**Figure S56.** Zoom of the 7.3-8.1 ppm range of the ^1^H NMR spectrum of **17.**

**
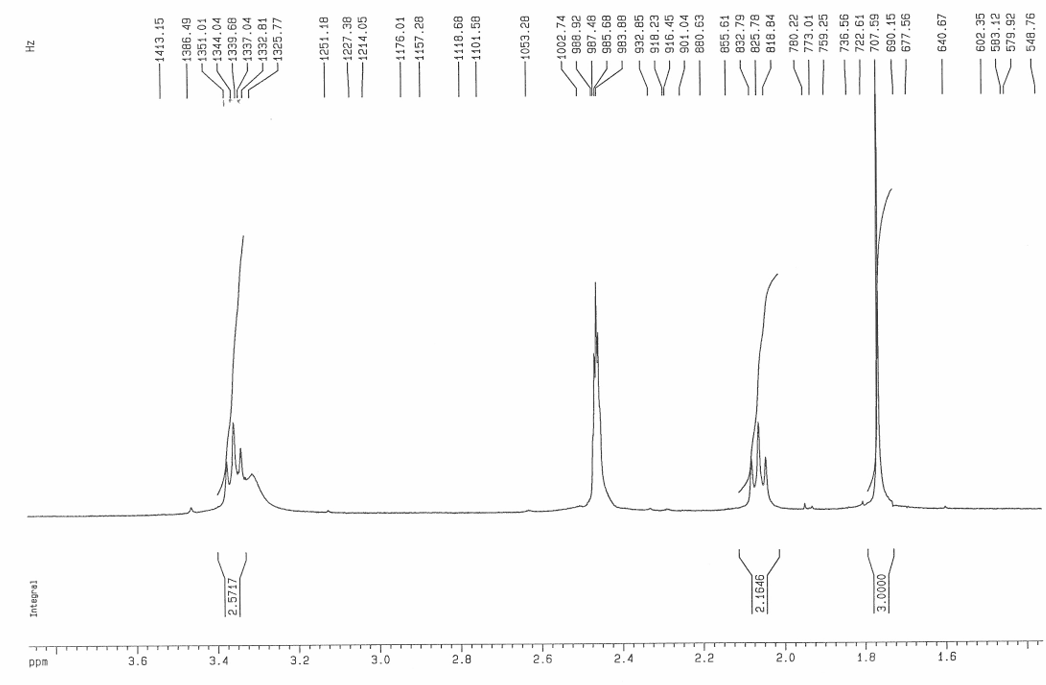
**

**Figure S57.** Zoom of the 1.5-3.7 ppm range of the ^1^H NMR spectrum of **17.**

**
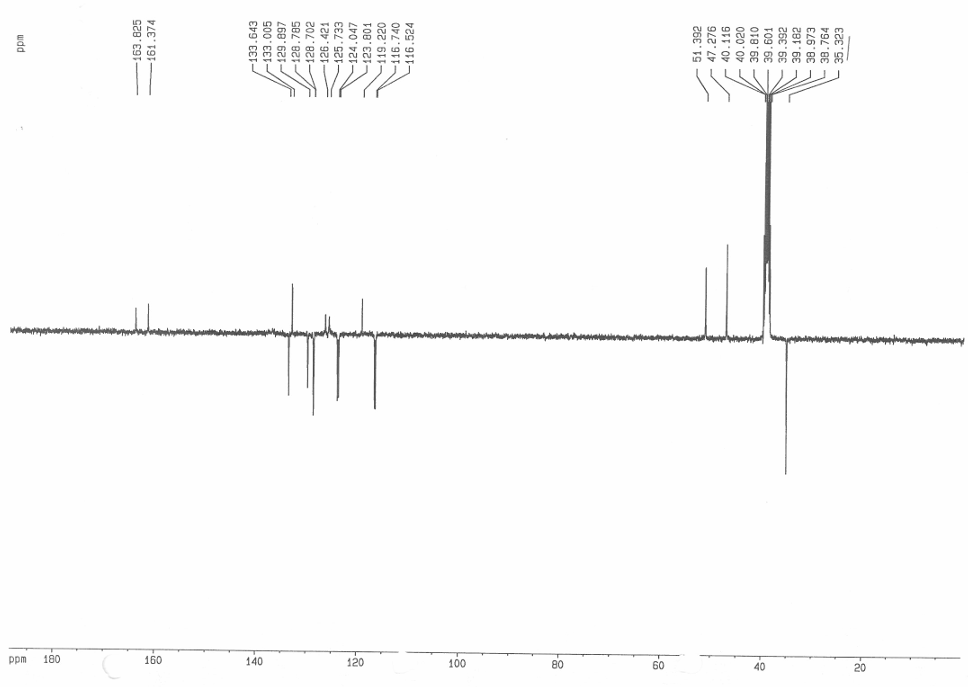
**

**Figure S58.** ^13^C NMR (101 MHz, DMSO-*d_6_*) spectrum of **17.**

**
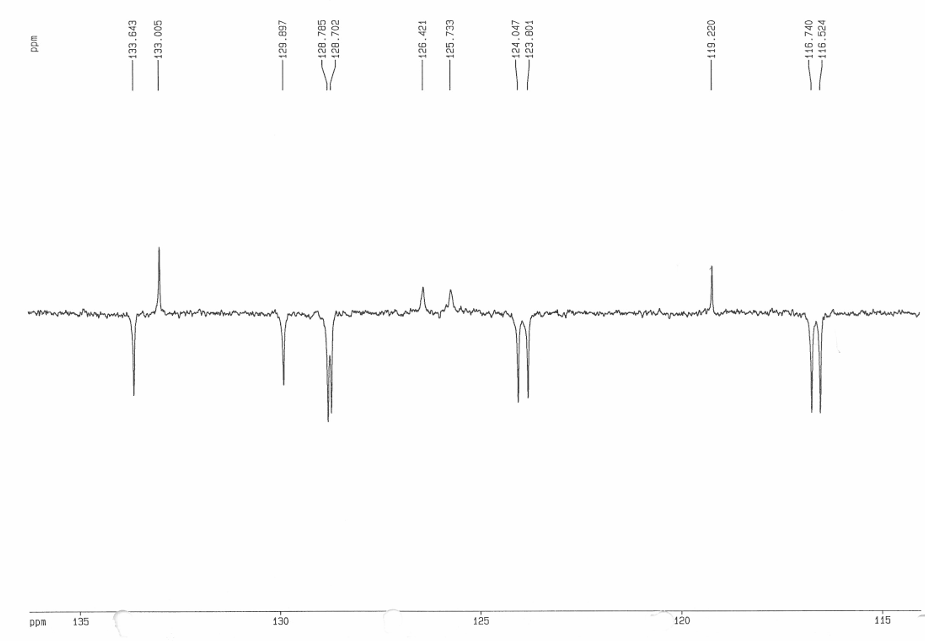
**

**Figure S59.** Zoom of the 115-135 ppm range of the ^13^C NMR spectrum of **17.**

**
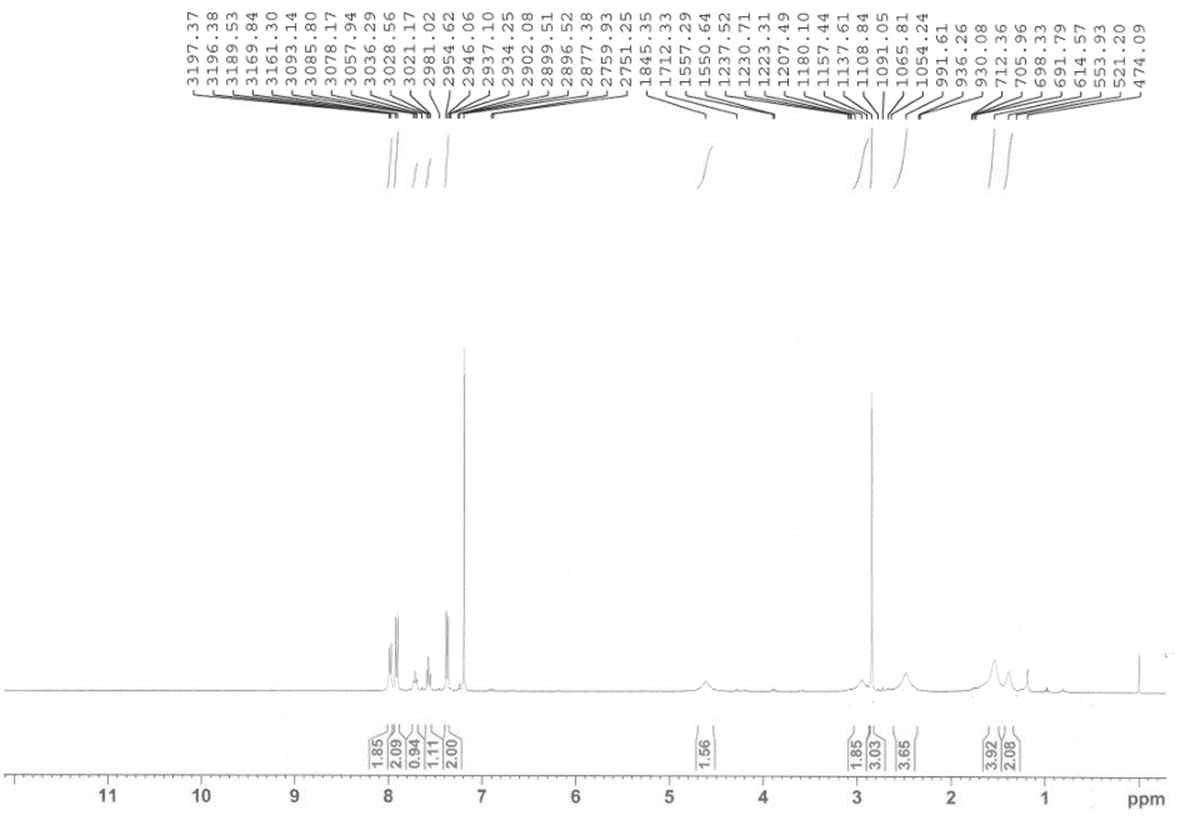
**

**Figure S60.** ^1^H NMR (400 MHz, CDCl_3_) spectrum of **18.**

**
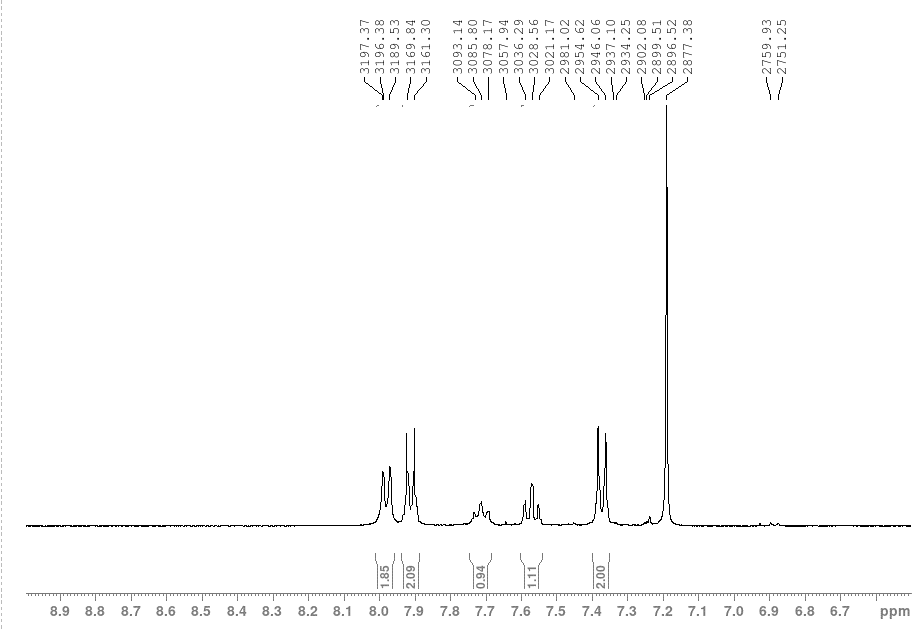
**

**Figure S61.** Zoom of the 6.5-9.0 ppm range of the ^1^H NMR spectrum of **18.**

**
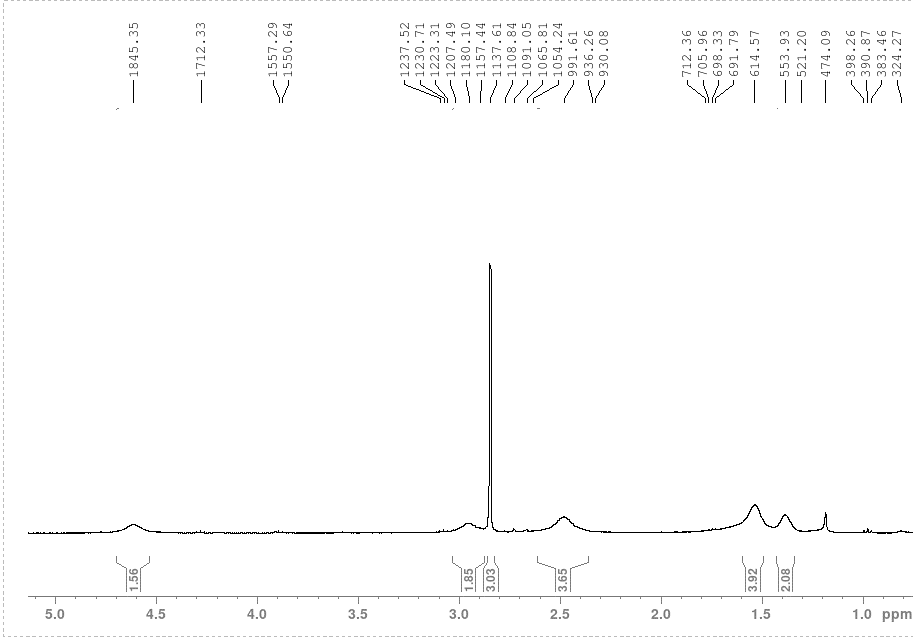
**

**Figure S62.** Zoom of the 0.8-5.1 ppm range of the ^1^H NMR spectrum of **18.**

**
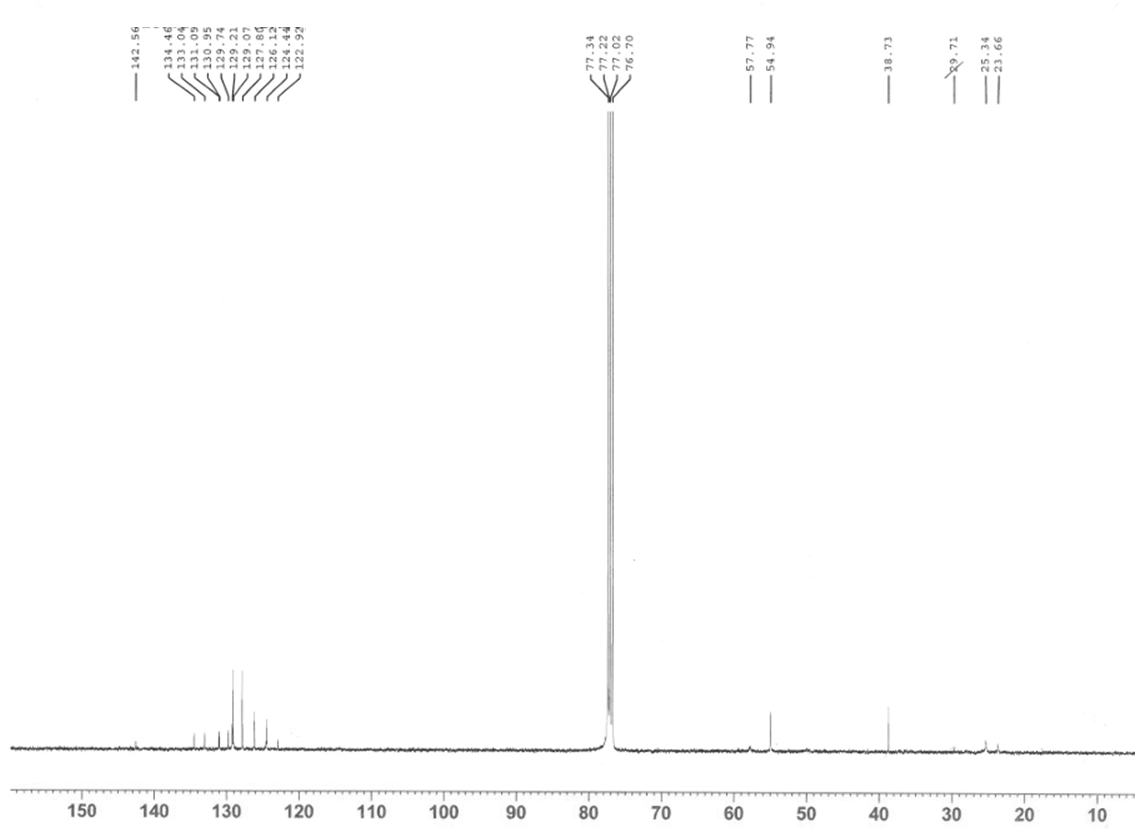
**

**Figure S63.** ^13^C NMR (101 MHz, CDCl_3_) spectrum of **18.**

**
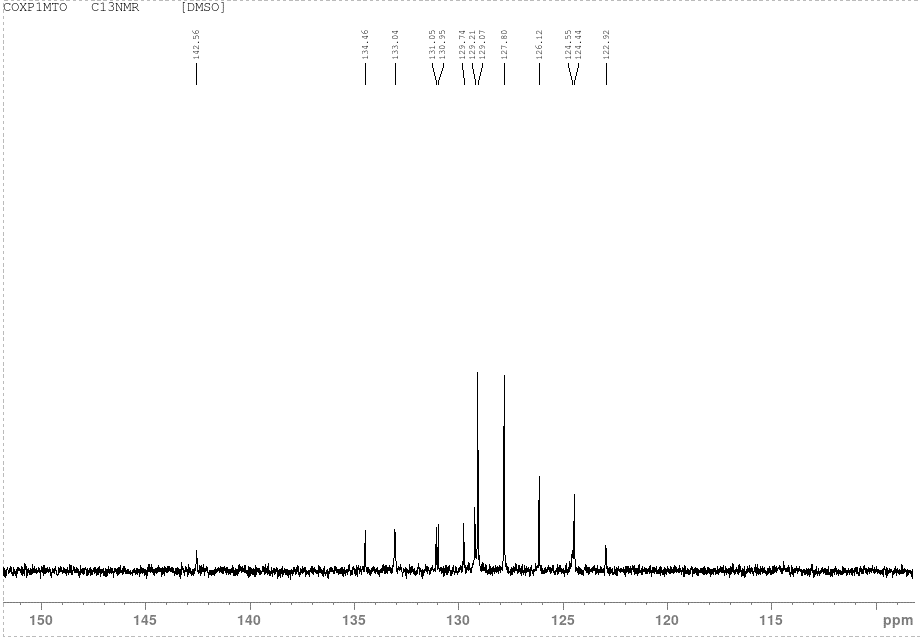
**

**Figure S64.** Zoom of the 108-150 ppm range of the ^13^C NMR spectrum of **18.**

**
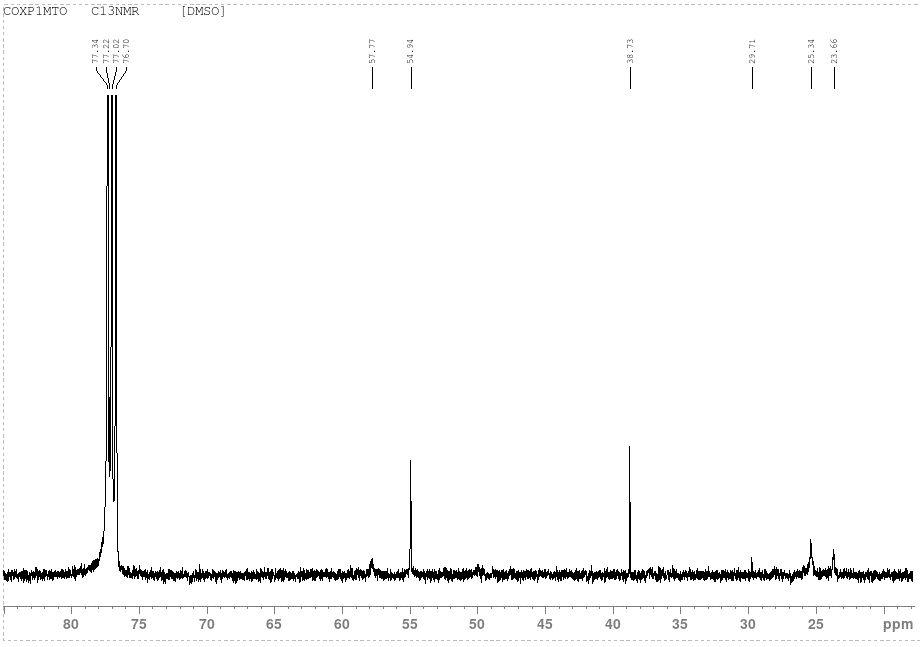
**

**Figure S65.** Zoom of the 18-85 ppm range of the ^13^C NMR spectrum of **18.**

**
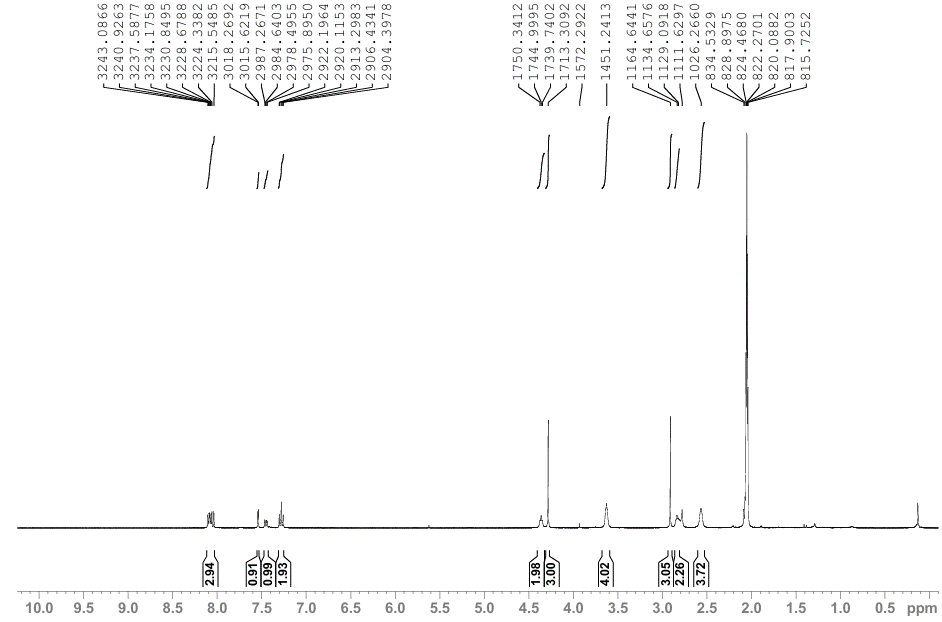
**

**Figure S66.** ^1^H NMR (400 MHz, Acetone) spectrum of **19.**

**
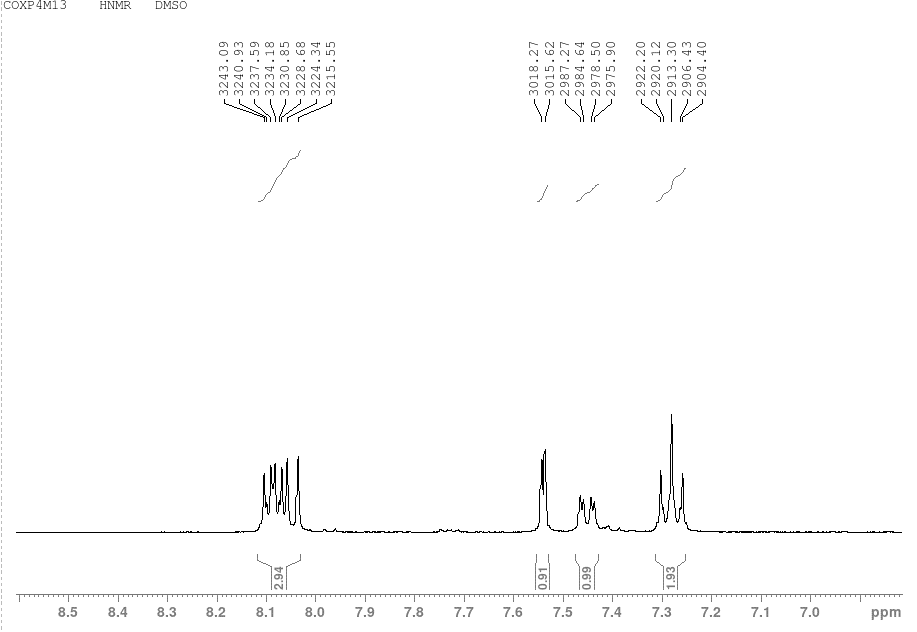
**

**Figure S67.** Zoom of the 6.8-8.5 ppm range of the ^1^H NMR spectrum of **19.**

**
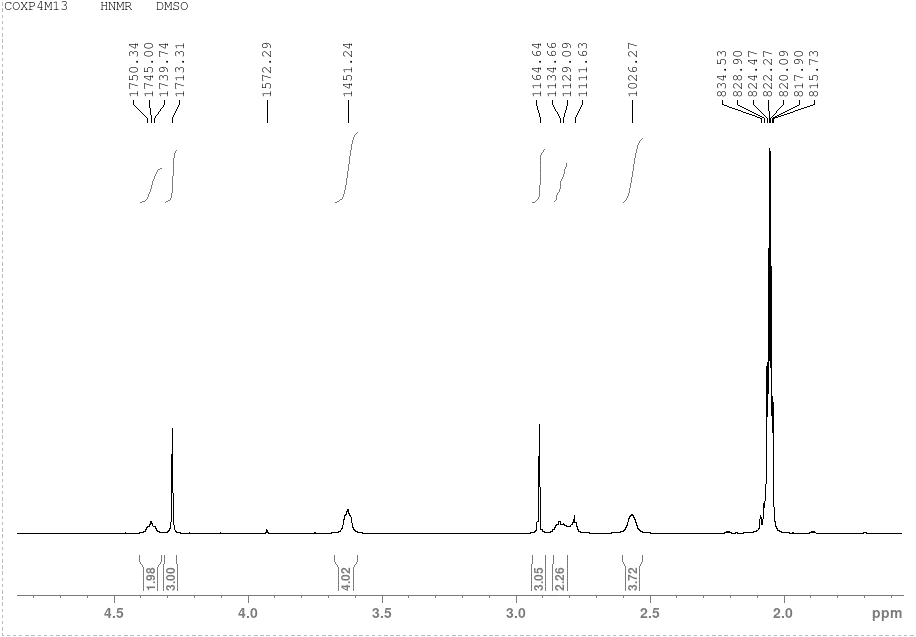
**

**Figure S68.** Zoom of the 1.5-5.0 ppm range of the ^1^H NMR spectrum of **19.**

**
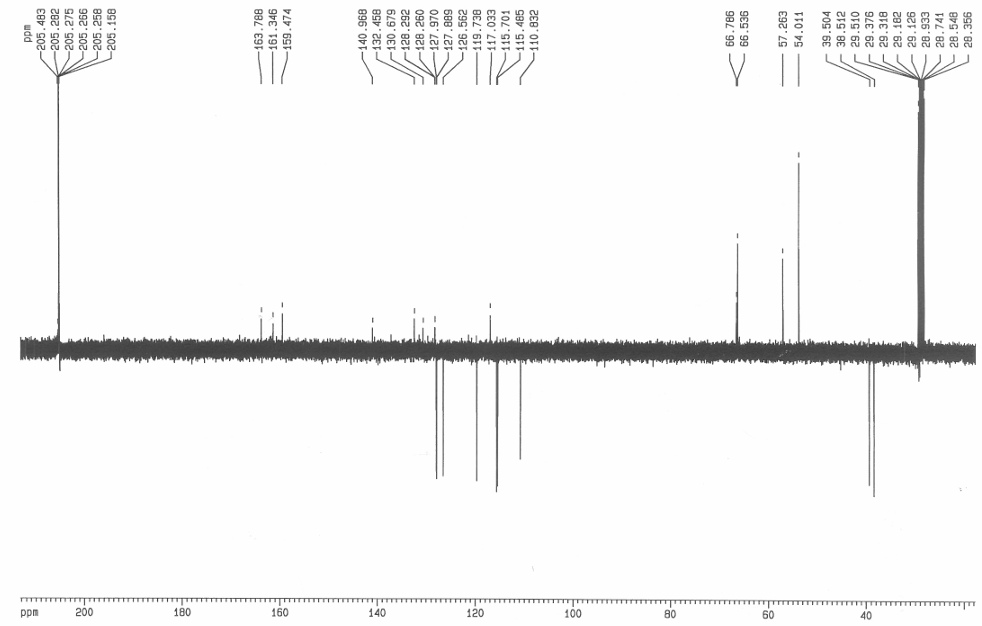
**

**Figure S69.** ^13^C NMR (101 MHz, Acetone) spectrum of **19.**

**
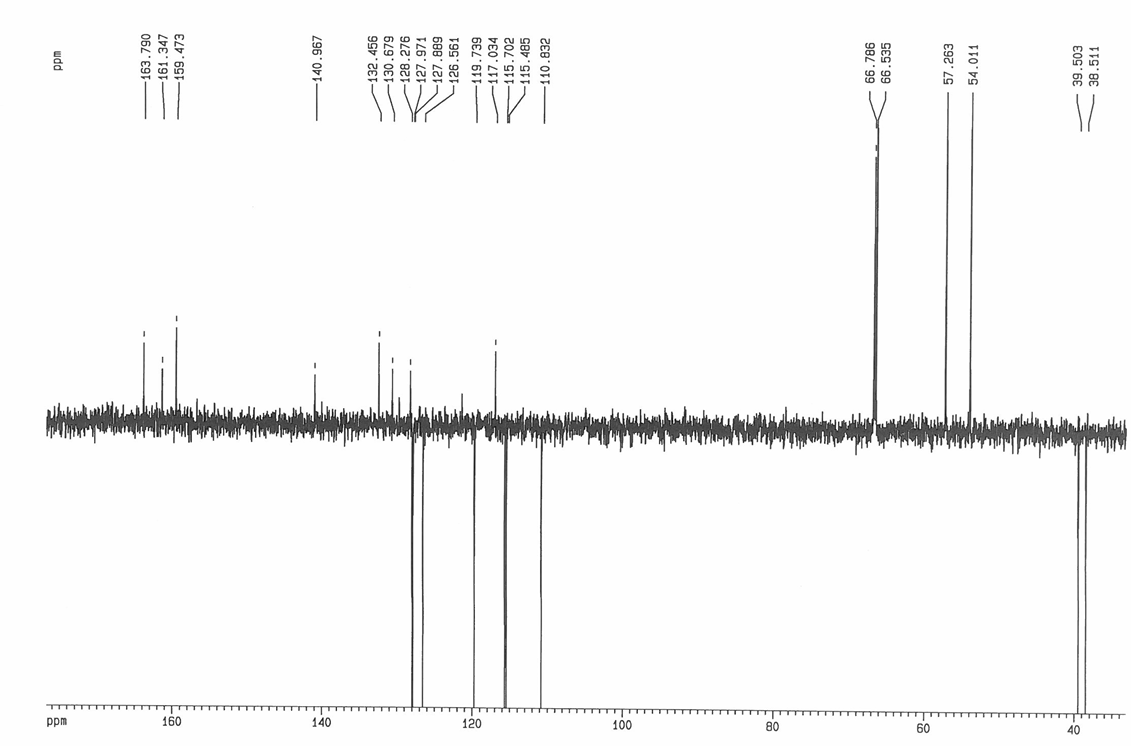
**

**Figure S70.** Zoom of the 35-175 ppm range of the ^13^C NMR spectrum of **19.**

**
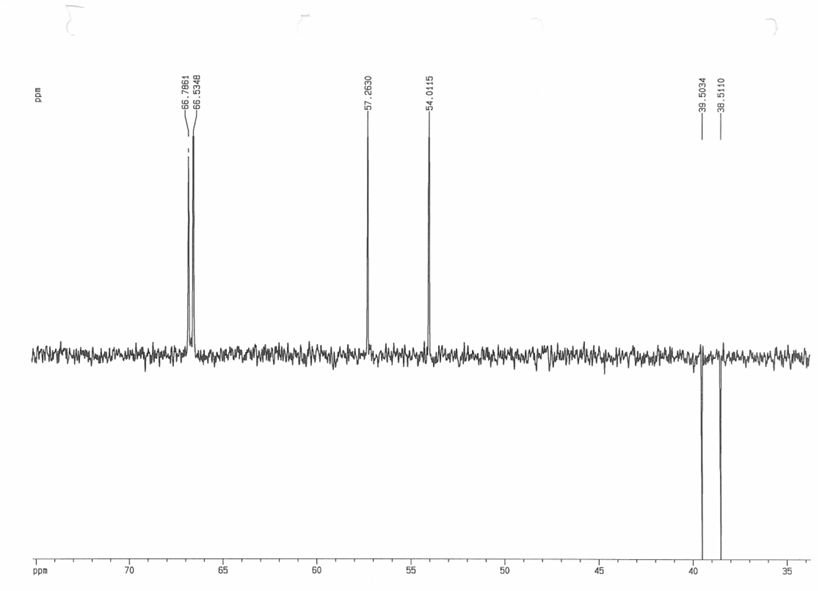
**

**Figure S71.** Zoom of the 35-80 ppm range of the ^13^C NMR spectrum of **19.**

**
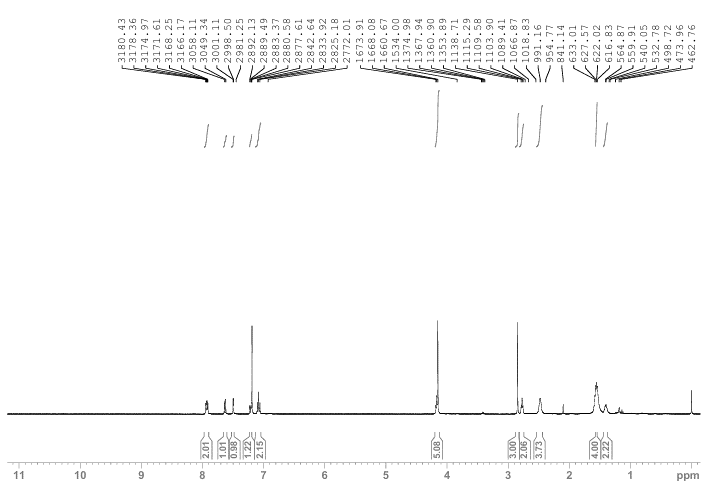
**

**Figure S72.** ^1^H NMR (400 MHz, CDCl_3_) spectrum of **20.**

**
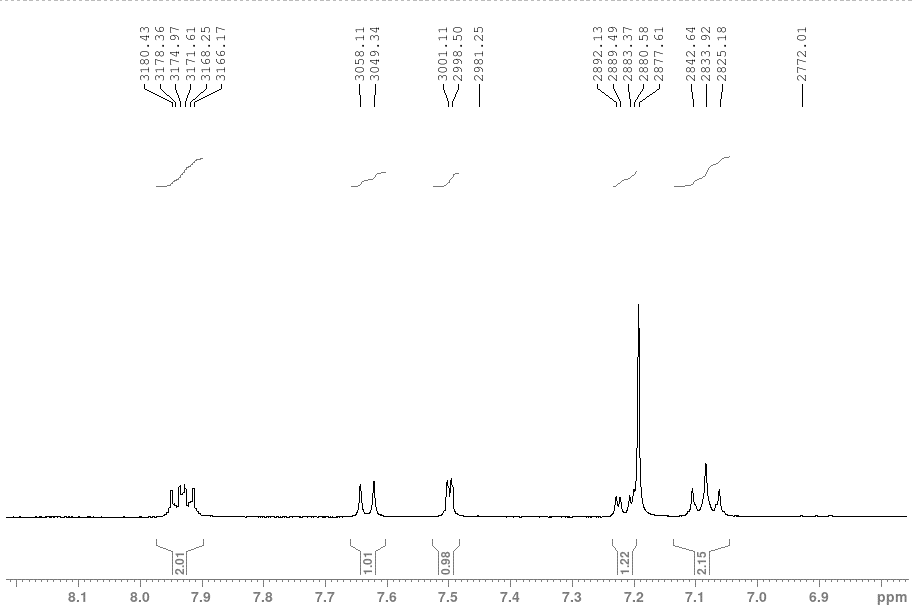
**

**Figure S73.** Zoom of the 6.8-8.2 ppm range of the ^1^H NMR spectrum of **20.**

**
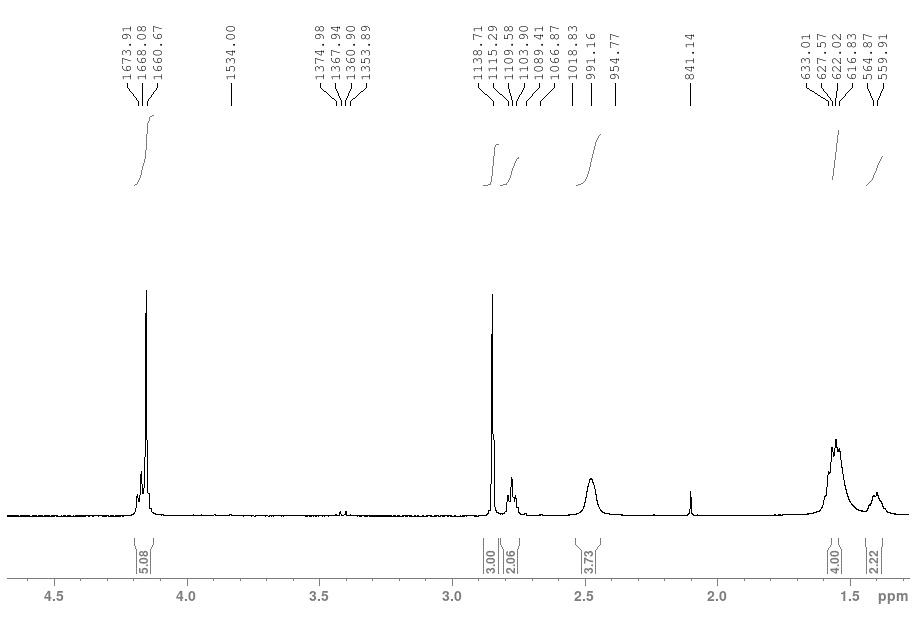
**

**Figure S74.** Zoom of the 1.3-4.5 ppm range of the ^1^H NMR spectrum of **20.**

**
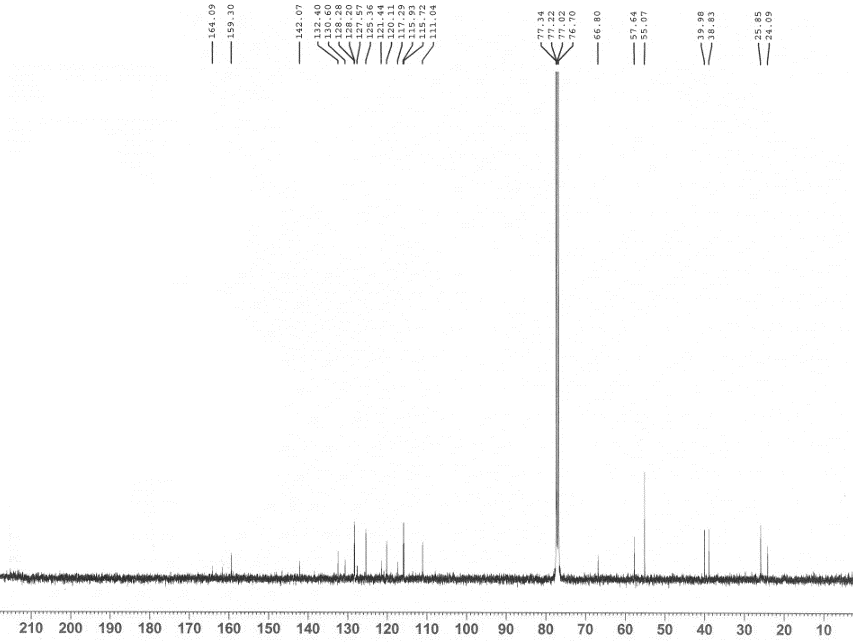
**

**Figure S75.** ^13^C NMR (101 MHz, CDCl_3_) spectrum of **20.**

**
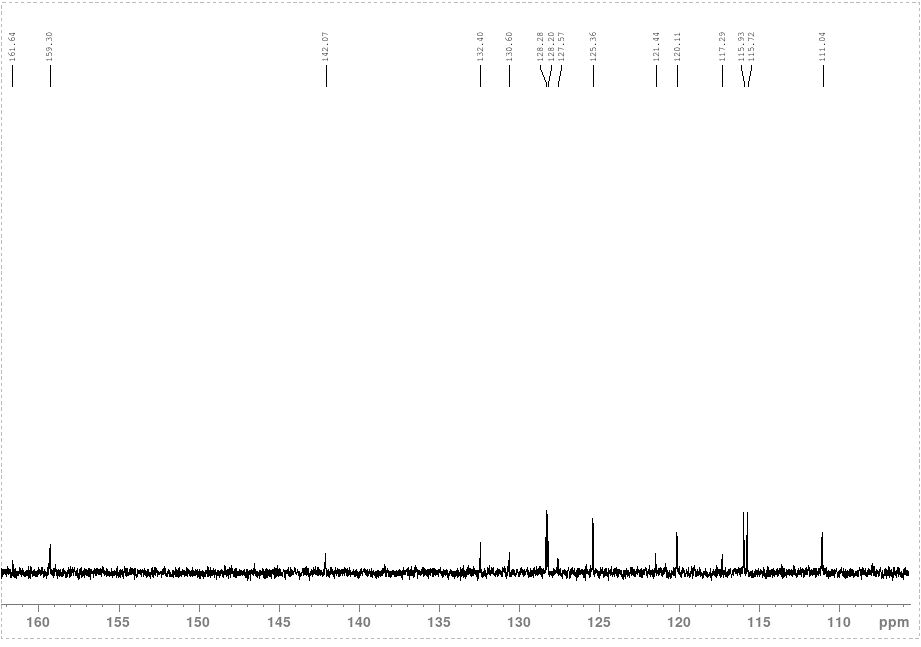
**

**Figure S76.** Zoom of the 105-160 ppm range of the ^13^C NMR spectrum of **20.**

**
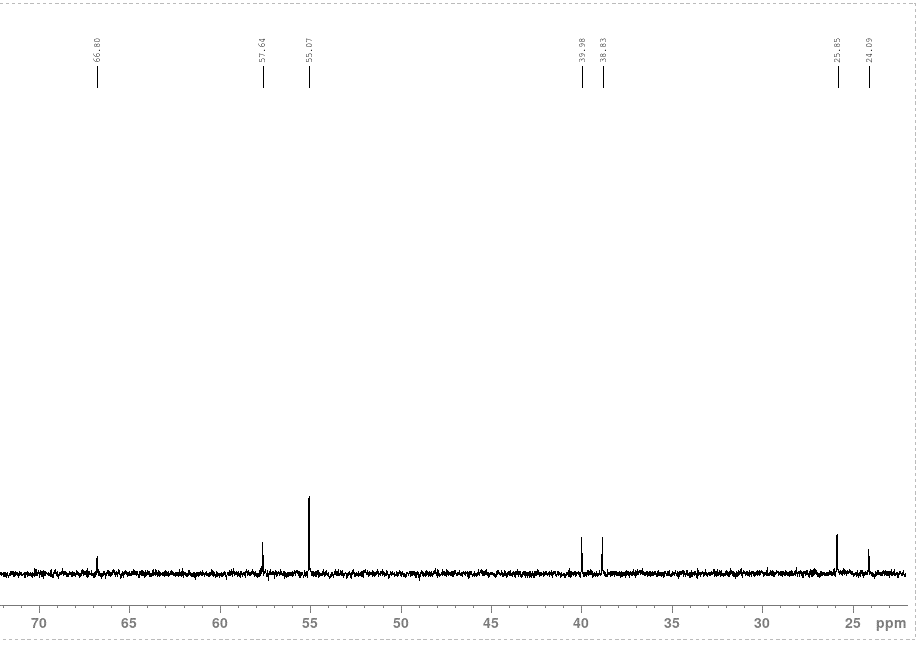
**

**Figure S77.** Zoom of the 20-70 ppm range of the ^13^C NMR spectrum of **20.**

**
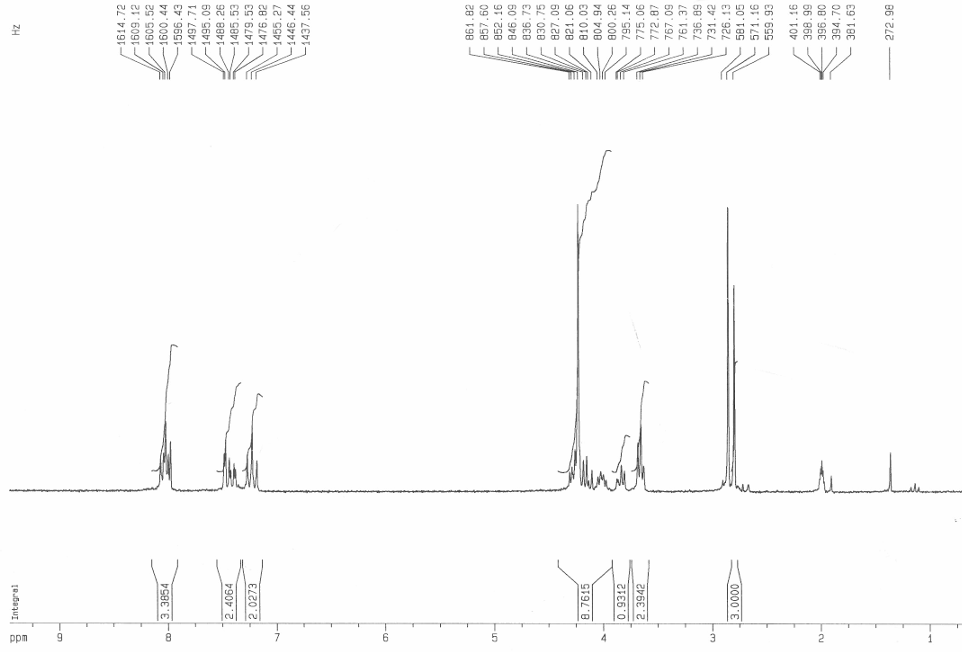
**

**Figure S78.** ^1^H NMR (200 MHz, Acetone) spectrum of **21.**

**
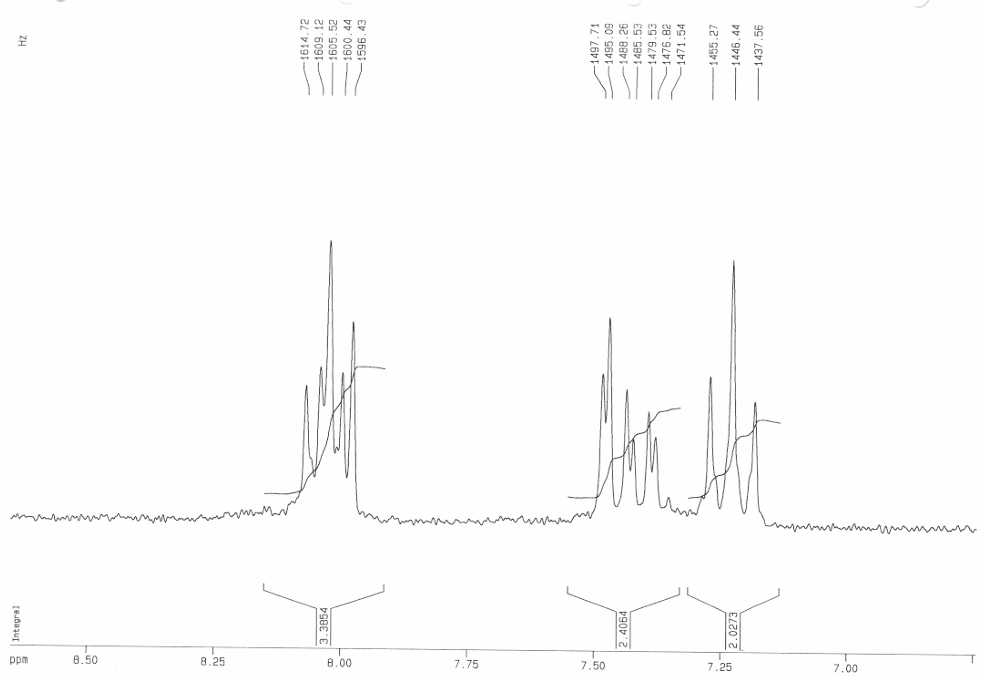
**

**Figure S79.** Zoom of the 6.5-8.5 ppm range of the ^1^H NMR spectrum of **21.**

**
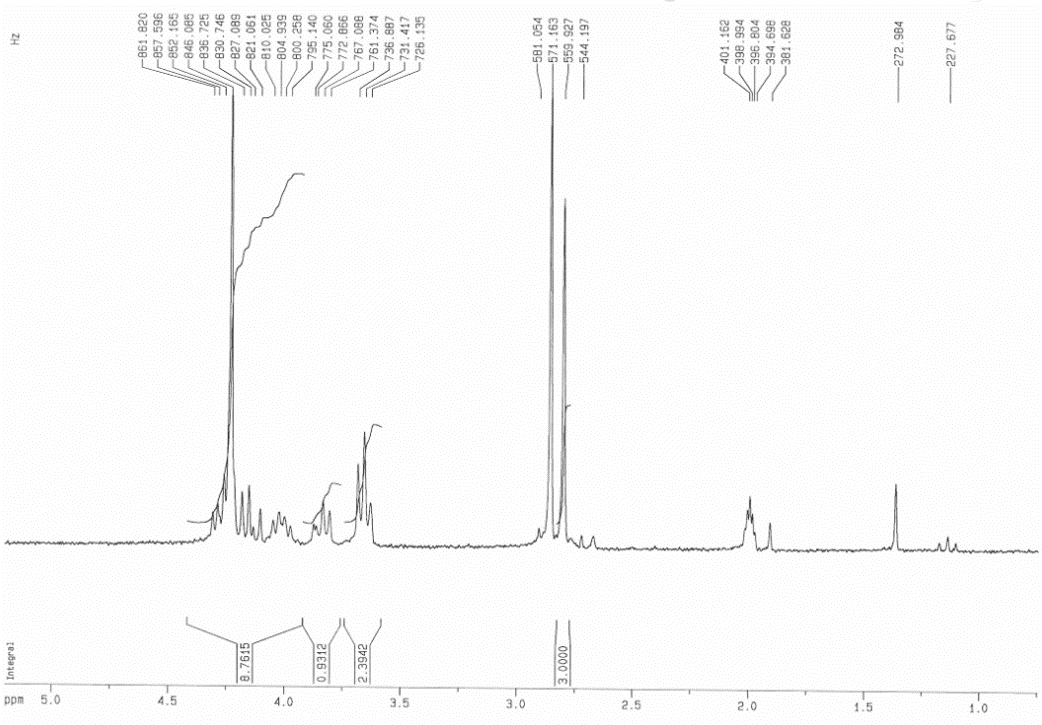
**

**Figure S80.** Zoom of the 0.5-5.0 ppm range of the ^1^H NMR spectrum of **21.**

**
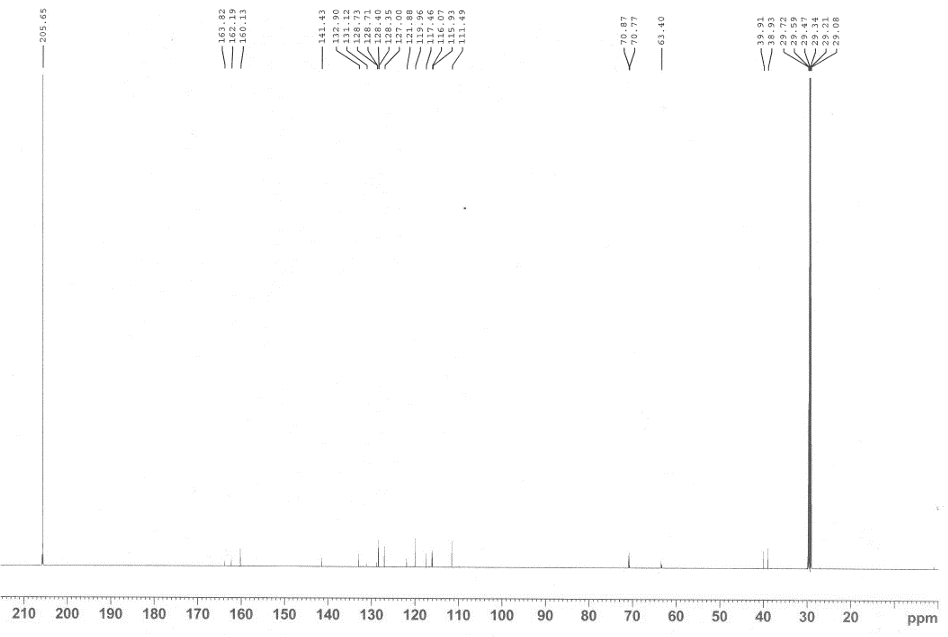
**

**Figure S81.** ^13^C NMR (101 MHz, Acetone) spectrum of **21.**

**
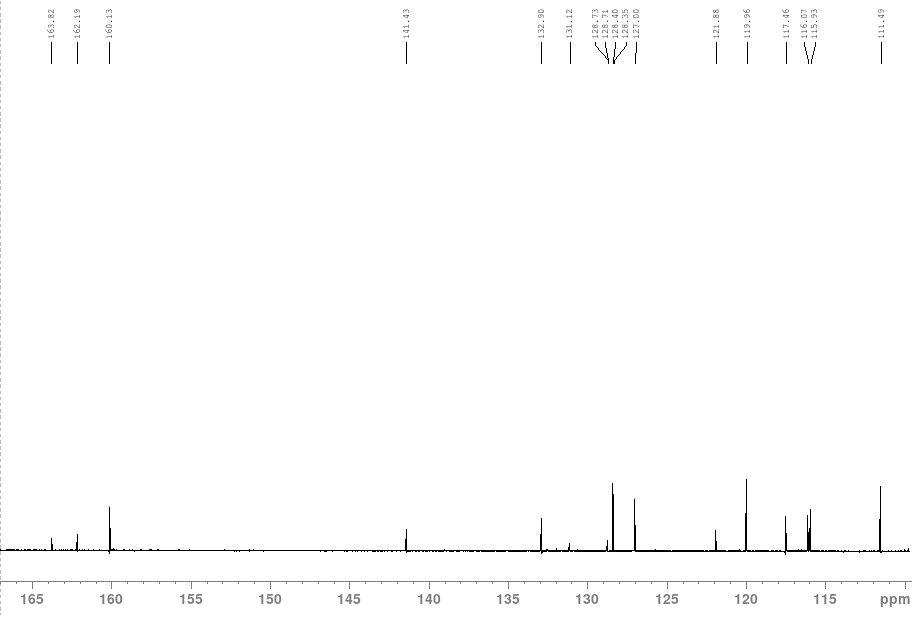
**

**Figure S82.** Zoom of the 110-165 ppm range of the ^13^C NMR spectrum of **21.**

**
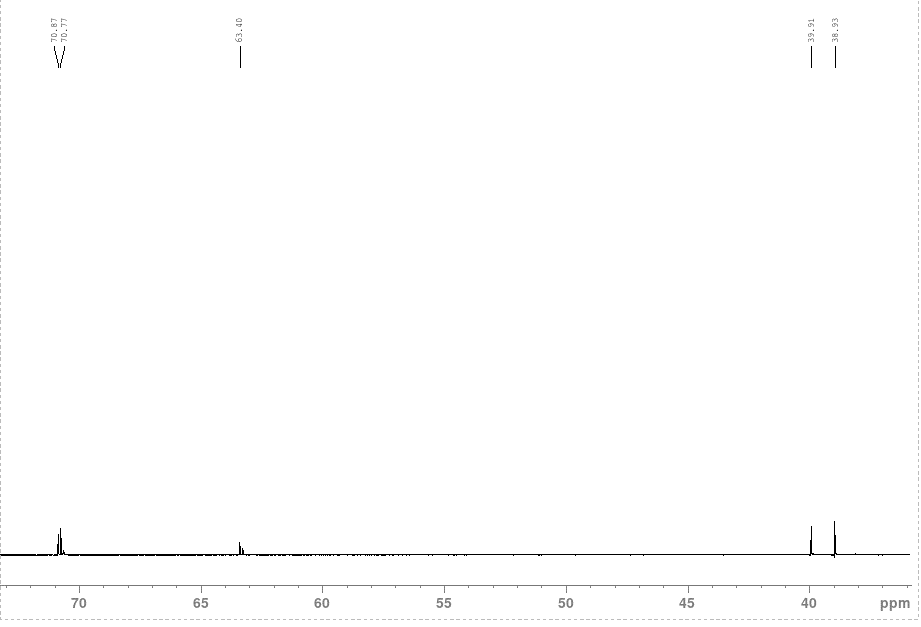
**

**Figure S83.** Zoom of the 35-75 ppm range of the ^13^C NMR spectrum of **21.**

**
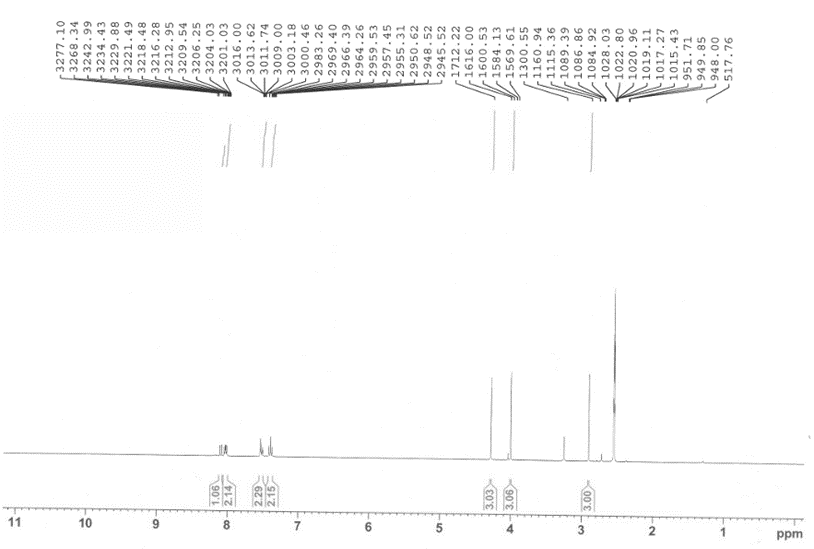
**

**Figure S84.** ^1^H NMR (400 MHz, DMSO-*_d6_*) spectrum of **22.**

**
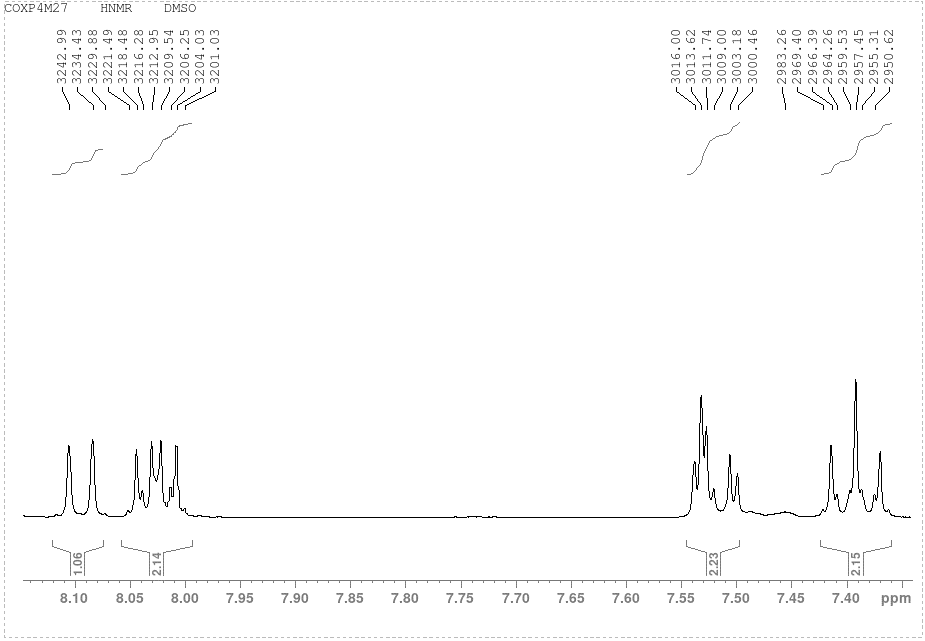
**

**Figure S85.** Zoom of the 7.3-8.2 ppm range of the ^1^H NMR spectrum of **22.**

**
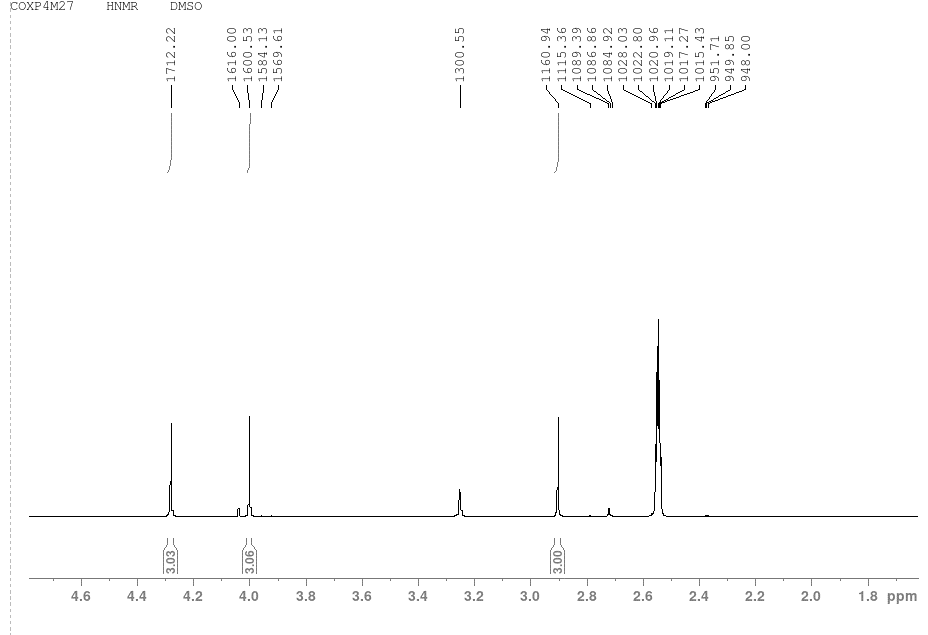
**

**Figure S86.** Zoom of the 1.6-4.8 ppm range of the ^1^H NMR spectrum of **22.
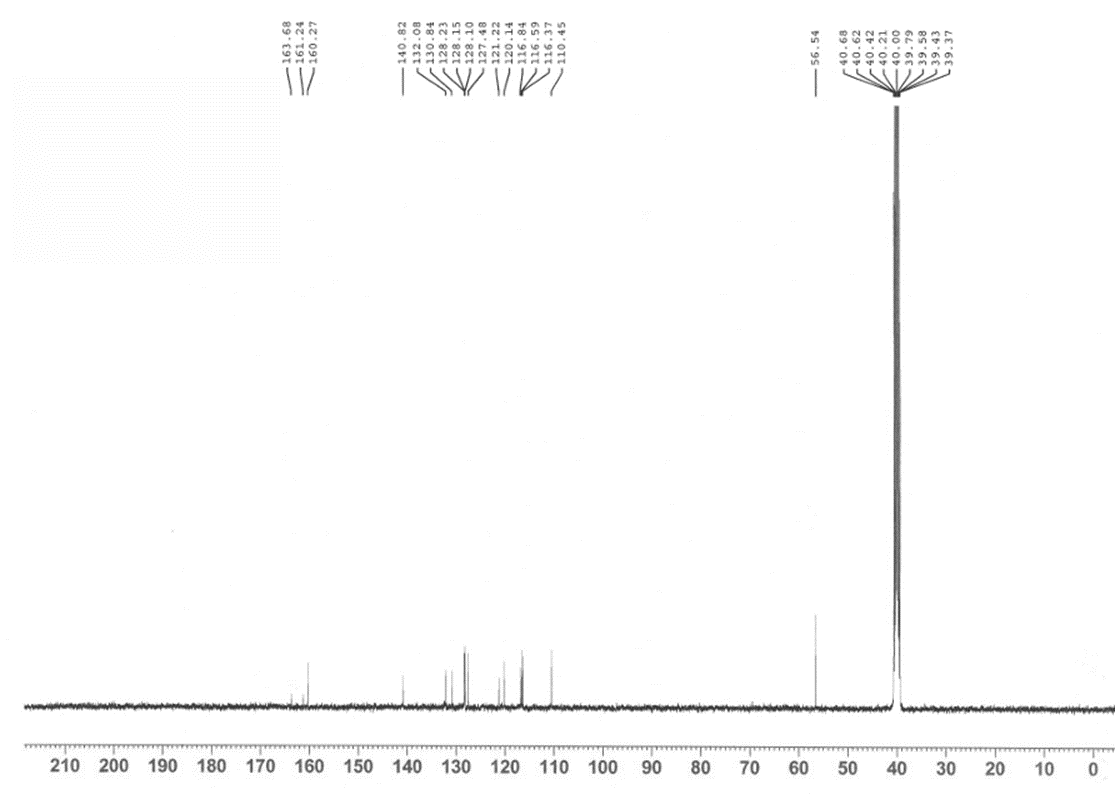
**

**Figure S87.** ^13^C NMR (101 MHz, DMSO-*_d6_*) spectrum of **22.**

**
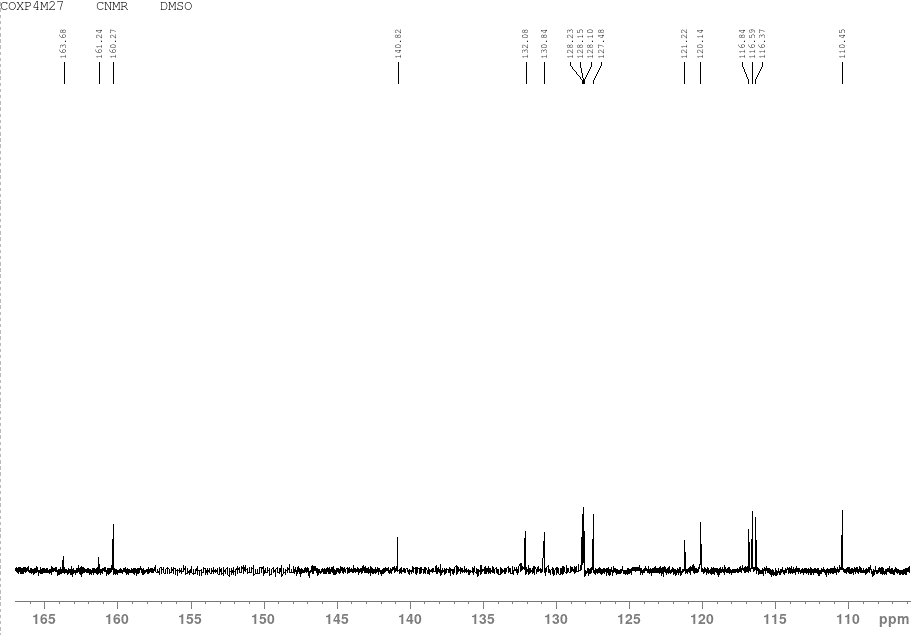
**

**Figure S88.** Zoom of the 100-165 ppm range of the ^13^C NMR spectrum of **22.**

**
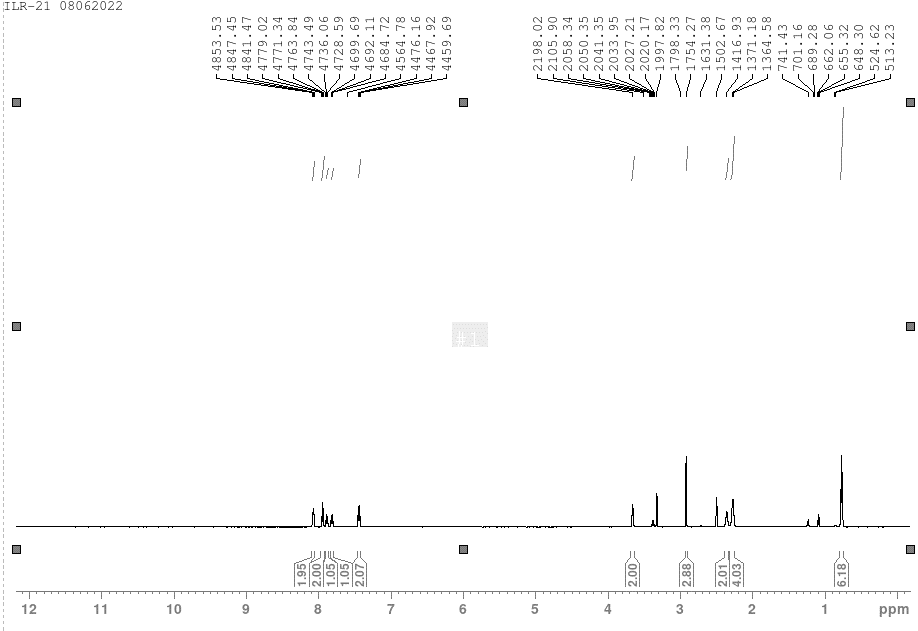
**

**Figure S89.** ^1^H NMR (600 MHz, DMSO-*_d6_*) spectrum of **23.**

**
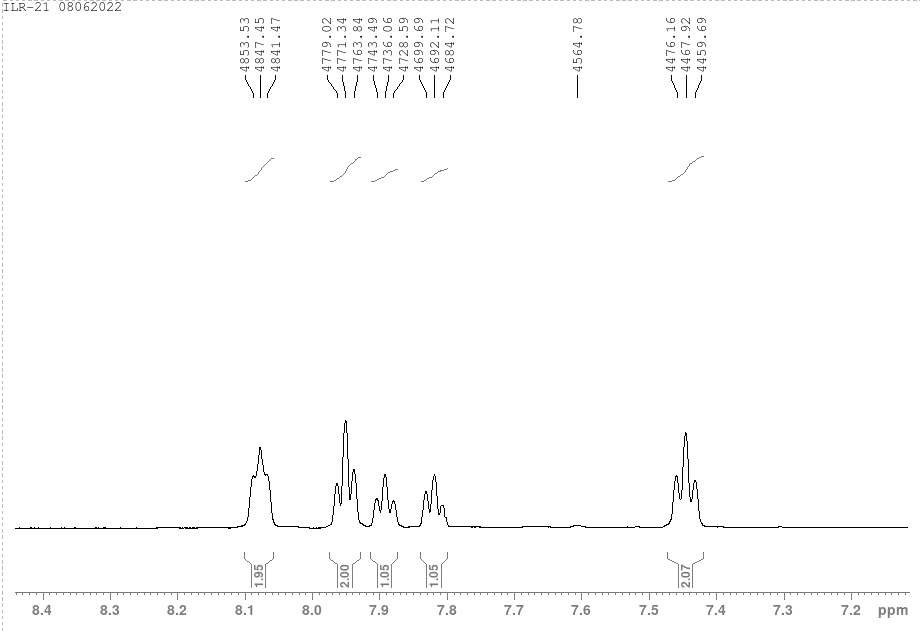
**

**Figure S90.** Zoom of the 7.1-8.4 ppm range of the ^1^H NMR spectrum of **23.**

**
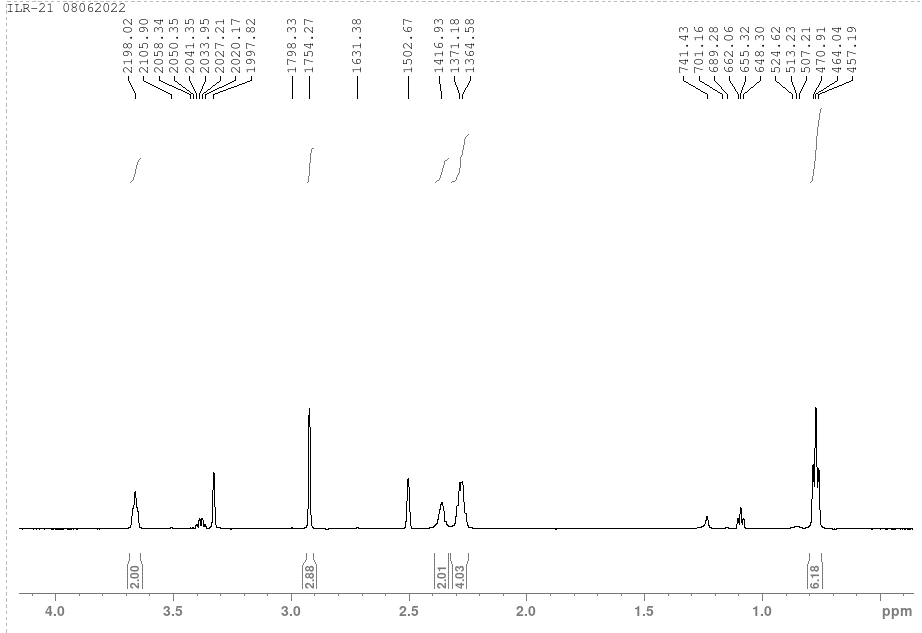
**

**Figure S91.** Zoom of the 0.5-4.0 ppm range of the ^1^H NMR spectrum of **23.**

**
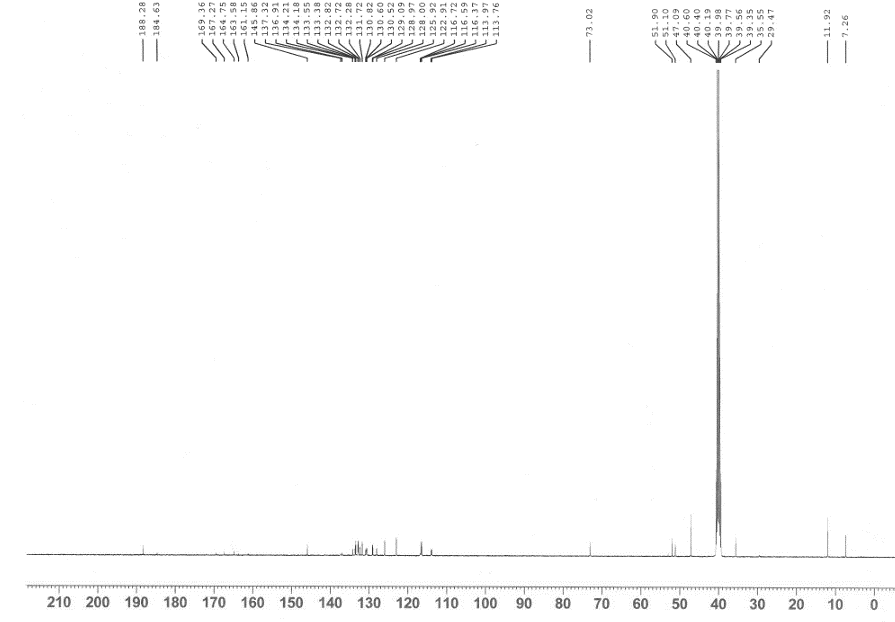
**

**Figure S92.** ^13^C NMR (101 MHz, DMSO-*_d6_*) spectrum of **23.**

**
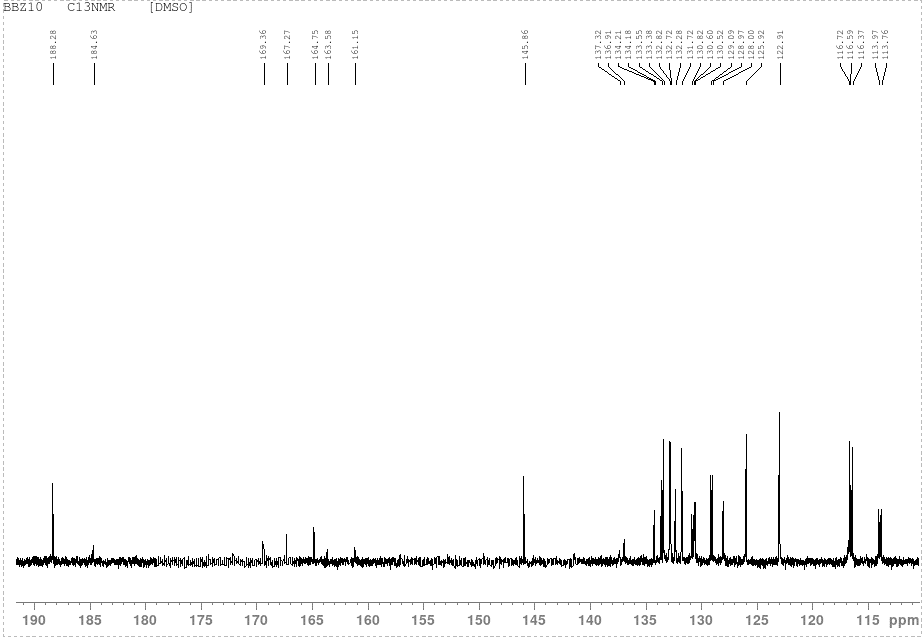
**

**Figure S93.** Zoom of the 110-190 ppm range of the ^13^C NMR spectrum of **23.**

**
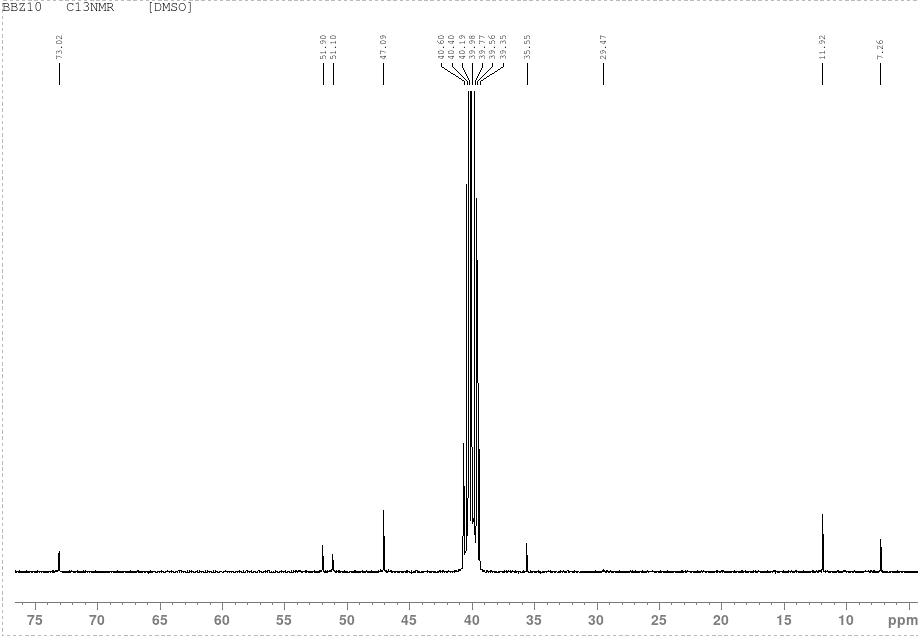
**

**Figure S94.** Zoom of the 5-75 ppm range of the ^13^C NMR spectrum of **23.**

**
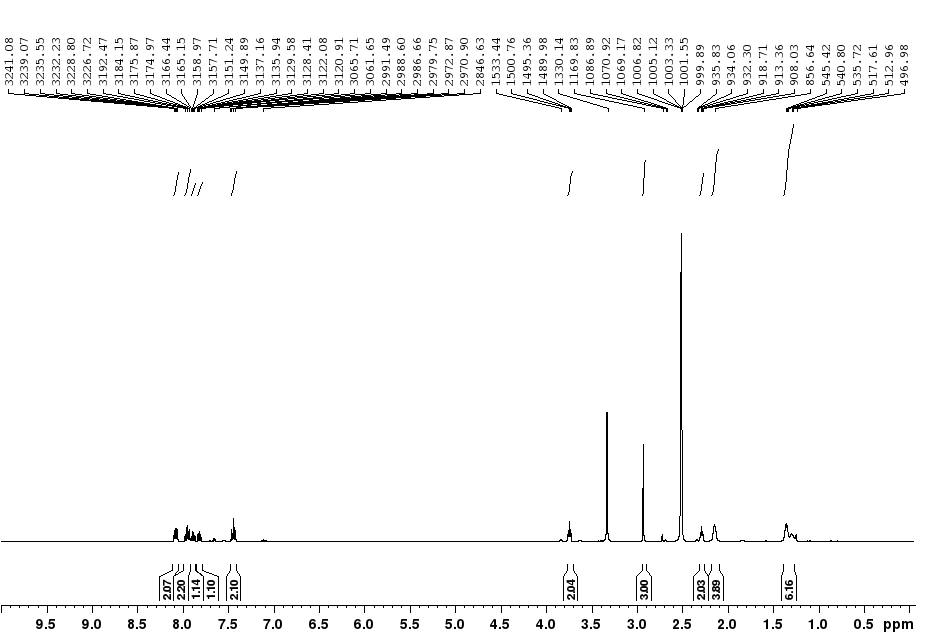
**

**Figure S95.** ^1^H NMR (600 MHz, DMSO-*_d6_*) spectrum of **24.**

**
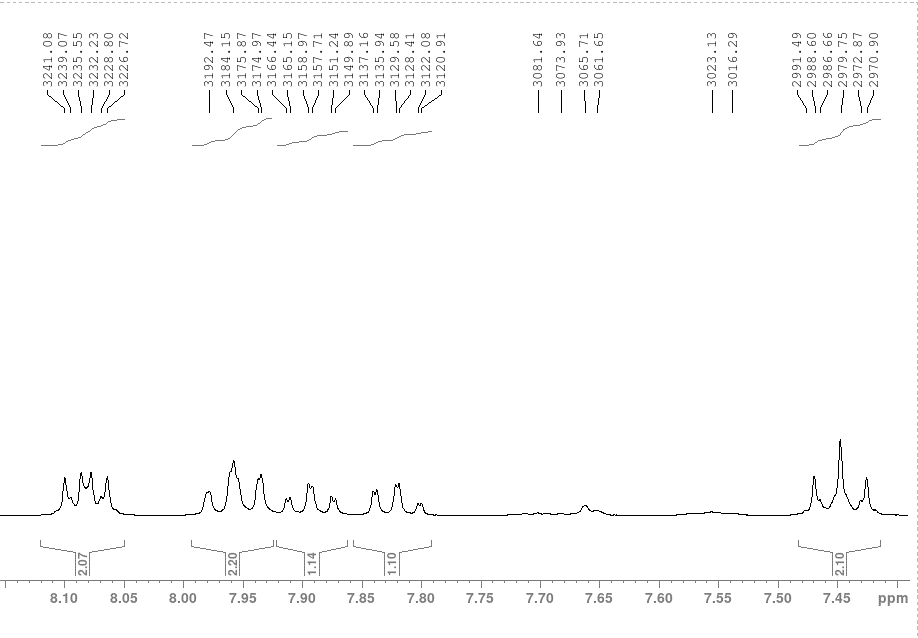
**

**Figure S96.** Zoom of the 7.4-8.2 ppm range of the ^1^H NMR spectrum of **24.**

**Figure S97.** Zoom of the 0.8-4.0 ppm range of the ^1^H NMR spectrum of **24.**

**Figure S98.** ^13^C NMR (101 MHz, DMSO-*_d6_*) spectrum of **24.**

**Figure S99.** Zoom of the 110-190 ppm range of the ^13^C NMR spectrum of **24.**

**Figure S100.** Zoom of the 20-75 ppm range of the ^13^C NMR spectrum of **24.**

**Figure S101.** HPLC of **5.**

**Figure S102.** HPLC of **7.**

**Figure S103.** HPLC of **8.**

**Figure S104.** HPLC of **9.**

**Figure S105.** HPLC of **10.**

**Figure S106.** HPLC of **11.**

**Figure S107.** HPLC of **12.**

**Figure S108.** HPLC of **13.**

**Figure S109.** HPLC of **14.**

**Figure S110.** HPLC of **15.**

**Figure S111.** HPLC of **16.**

**Figure S112.** HPLC of **17.**

**Figure S113.** HPLC of **18.**

**Figure S114.** HPLC of **19.**

**Figure S115.** HPLC of **20.**

**Figure S116.** HPLC of **21.**

**Figure S117.** HPLC of **22.**

**Figure S118.** HPLC of **23.**

**Figure S119.** HPLC of **24.**
