# Supplementary material for: NorA Efflux Pump Inhibitors: Expanding SAR Knowledge of Pyrazolo[4,3‐c][1,2]benzothiazine 5,5‐Dioxide Derivatives
Source: Arch Pharm (Weinheim). 2025 May 19;358(5):e70000. doi: 10.1002/ardp.70000 (PMC12089509; doi:10.1002/ardp.70000)
Supplement: Supplementary file 2 — ArchPharm_SupplMat_InChI. [file ARDP-358-e70000-s002.doc]

**Supplemental Material: Novel Compounds and Biological Screening Results**

**NorA Efflux Pump Inhibitors: Expanding SAR Knowledge of Pyrazolo[4,3-c][1,2]benzothiazine 5,5-dioxide Derivatives**

**Authors**

Giada Cernicchi^1^, Alessandra Di Gregorio^2^, Tommaso Felicetti^1,*^, Elisa Rampacci^3^, Giulia Casari^4^, Tatiana Armeni^4^, Brenda Romaldi^4^, Ermelinda Zefaj^1^, Fabrizio Passamonti^3^, Serena Massari^1^, Giuseppe Manfroni^1^, Maria Letizia Barreca^1^, Oriana Tabarrini^1^, Carla Vignaroli^2,*^, Stefano Sabatini^1^.

**Affilitation**

^1^Department of Pharmaceutical Sciences, Università degli Studi di Perugia, via del Liceo 1, 06123, Perugia, Italy

^2^Department of Life and Environmental Science, Università Politecnica delle Marche, via Brecce Bianche, 60131, Ancona, Italy

^3^Department of Veterinary Medicine, Università degli Studi di Perugia, via San Costanzo 4, 06126, Perugia, Italy

^4^Dipartimento di Scienze Cliniche Specialistiche ed Odontostomatologiche-Sez. Biochimica, Biologia e Fisica, Università Politecnica delle Marche, 60131 Ancona, Italy

*Corresponding author; E-mail addresses: tommaso.felicetti@unipg.it (T. Felicetti), c.vignaroli@univpm.it (C. Vignaroli).

| **Compound No.** | **InChI** | **Biological Activity (EtBr % inhibition at 50µM)^a^** |
| --- | --- | --- |
| **1** | InChI=1S/C21H13ClN4O4S/c22-14-7-5-13(6-8-14)19-20-21(17-3-1-2-4-18(17)31(29,30)24-20)25(23-19)15-9-11-16(12-10-15)26(27)28/h1-12,24H | 77 |
| **2** | InChI=1S/C22H15ClN4O4S/c1-25-22-20(14-6-8-15(23)9-7-14)24-26(16-10-12-17(13-11-16)27(28)29)21(22)18-4-2-3-5-19(18)32(25,30)31/h2-13H,1H3 | 0 |
| **3** | InChI=1S/C21H14ClN3O2S/c22-15-12-10-14(11-13-15)19-20-21(25(23-19)16-6-2-1-3-7-16)17-8-4-5-9-18(17)28(26,27)24-20/h1-13,24H | 7 |
| **4** | InChI=1S/C16H12N4O4S/c1-10-15-16(13-4-2-3-5-14(13)25(23,24)18-15)19(17-10)11-6-8-12(9-7-11)20(21)22/h2-9,18H,1H3 | 0 |
| **5** | InChI=1S/C16H12N4O4S/c1-10-16-15(13-4-2-3-5-14(13)18-25(16,23)24)19(17-10)11-6-8-12(9-7-11)20(21)22/h2-9,18H,1H3 | 0 |
| **6** | InChI=1S/C17H14N4O4S/c1-11-16-17(14-5-3-4-6-15(14)26(24,25)19(16)2)20(18-11)12-7-9-13(10-8-12)21(22)23/h3-10H,1-2H3 | 22 |
| **7** | InChI=1S/C17H14N4O4S/c1-11-17-16(14-5-3-4-6-15(14)19(2)26(17,24)25)20(18-11)12-7-9-13(10-8-12)21(22)23/h3-10H,1-2H3 | 3 |
| **8** | InChI=1S/C17H17N3O2S/c1-13-12-17(20(18-13)14-8-4-3-5-9-14)15-10-6-7-11-16(15)19-23(2,21)22/h3-12,19H,1-2H3 | 0 |
| **9** | InChI=1S/C15H10ClN3O2S/c16-10-7-5-9(6-8-10)13-15-14(18-17-13)11-3-1-2-4-12(11)22(20,21)19-15/h1-8,19H,(H,17,18) | 12 |
| **10** | InChI=1S/C16H12ClN3O2S/c1-20-16-14(10-6-8-11(17)9-7-10)18-19-15(16)12-4-2-3-5-13(12)23(20,21)22/h2-9H,1H3,(H,18,19) | 100 |
| **11** | InChI=1S/C16H13N3O2S/c1-19-16-14(11-7-3-2-4-8-11)17-18-15(16)12-9-5-6-10-13(12)22(19,20)21/h2-10H,1H3,(H,17,18) | 68 |
| **12** | InChI=1S/C16H12FN3O2S/c1-20-16-14(10-6-8-11(17)9-7-10)18-19-15(16)12-4-2-3-5-13(12)23(20,21)22/h2-9H,1H3,(H,18,19) | 87 |
| **13** | InChI=1S/C16H11ClFN3O2S/c1-21-16-14(9-6-7-12(18)11(17)8-9)19-20-15(16)10-4-2-3-5-13(10)24(21,22)23/h2-8H,1H3,(H,19,20) | 100 |
| **14** | InChI=1S/C17H14FN3O2S/c1-2-21-17-15(11-7-9-12(18)10-8-11)19-20-16(17)13-5-3-4-6-14(13)24(21,22)23/h3-10H,2H2,1H3,(H,19,20) | 57 |
| **15** | InChI=1S/C18H16FN3O3S/c19-13-8-6-12(7-9-13)16-18-17(21-20-16)14-4-1-2-5-15(14)26(24,25)22(18)10-3-11-23/h1-2,4-9,23H,3,10-11H2,(H,20,21) | 17 |
| **16** | InChI=1S/C18H15FN4O3S/c1-20-15(24)10-23-18-16(11-6-8-12(19)9-7-11)21-22-17(18)13-4-2-3-5-14(13)27(23,25)26/h2-9H,10H2,1H3,(H,20,24)(H,21,22) | 22 |
| **17** | InChI=1S/C18H17FN4O2S/c1-20-10-11-23-18-16(12-6-8-13(19)9-7-12)21-22-17(18)14-4-2-3-5-15(14)26(23,24)25/h2-9,20H,10-11H2,1H3,(H,21,22) | 57 |
| **18** | InChI=1S/C23H25ClN4O2S/c1-26-23-21(17-9-11-18(24)12-10-17)25-28(16-15-27-13-5-2-6-14-27)22(23)19-7-3-4-8-20(19)31(26,29)30/h3-4,7-12H,2,5-6,13-16H2,1H3 | 79 |
| **19** | InChI=1S/C23H25FN4O4S/c1-26-22-19-8-7-18(32-14-11-28-9-12-31-13-10-28)15-20(19)33(29,30)27(2)23(22)21(25-26)16-3-5-17(24)6-4-16/h3-8,15H,9-14H2,1-2H3 | 90 |
| **20** | InChI=1S/C24H27FN4O3S/c1-27-23-20-11-10-19(32-15-14-29-12-4-3-5-13-29)16-21(20)33(30,31)28(2)24(23)22(26-27)17-6-8-18(25)9-7-17/h6-11,16H,3-5,12-15H2,1-2H3 | 77 |
| **21** | InChI=1S/C20H20FN3O5S/c1-23-19-16-8-7-15(29-11-14(26)10-25)9-17(16)30(27,28)24(2)20(19)18(22-23)12-3-5-13(21)6-4-12/h3-9,14,25-26H,10-11H2,1-2H3 | 0 |
| **22** | InChI=1S/C18H16FN3O3S/c1-21-17-14-9-8-13(25-3)10-15(14)26(23,24)22(2)18(17)16(20-21)11-4-6-12(19)7-5-11/h4-10H,1-3H3 | 22 |
| **23** | InChI=1S/C22H25FN2O4S/c1-4-25(5-2)14-15-29-22-18-8-6-7-9-19(18)30(27,28)24(3)20(22)21(26)16-10-12-17(23)13-11-16/h6-13H,4-5,14-15H2,1-3H3 | 36 |
| **24** | InChI=1S/C23H25FN2O4S/c1-25-21(22(27)17-9-11-18(24)12-10-17)23(30-16-15-26-13-5-2-6-14-26)19-7-3-4-8-20(19)31(25,28)29/h3-4,7-12H,2,5-6,13-16H2,1H3 | 38 |

^a^ Experiments were performed in triplicate with two technical replicates per biological replicate. Efflux activity of SA-1199B was expressed as percent fluorescence decrease over a 5 min time course. Inhibition of this efflux by test compounds was determined using the equation [efflux in the absence] − [efflux in the presence of test [compound]/[efflux in the absence of test compound] × 100, giving the percent efflux inhibition observed.
